# Resistance to Sharka in Apricot: Comparison of Phase-Reconstructed Resistant and Susceptible Haplotypes of ‘Lito’ Chromosome 1 and Analysis of Candidate Genes

**DOI:** 10.3389/fpls.2019.01576

**Published:** 2019-12-04

**Authors:** Gloria De Mori, Rachele Falchi, Raffaele Testolin, Daniele Bassi, Federica Savazzini, Luca Dondini, Stefano Tartarini, Francesco Palmisano, Angelantonio Minafra, Alessandro Spadotto, Simone Scalabrin, Filippo Geuna

**Affiliations:** ^1^Department of Agricultural, Food, Environmental and Animal Sciences, University of Udine, Udine, Italy; ^2^Department of Agricultural and Environmental Sciences (DISAA), University of Milan, Milan, Italy; ^3^Department of Agricultural Sciences, University of Bologna, Bologna, Italy; ^4^Centro di Ricerca, Sperimentazione e Formazione in Agricoltura “Basile Caramia”, Locorotondo, Italy; ^5^National Research Council, Institute for Sustainable Plant Protection, Bari, Italy; ^6^IGA Technology Services, Science and Technology Park, ZIU, Udine, Italy

**Keywords:** *Plum Pox Virus*, Prunus armeniaca, R genes, genome sequencing, genome assembly, gene prediction, allele-specific expression

## Abstract

Sharka, a common disease among most stone fruit crops, is caused by the *Plum Pox Virus* (PPV). Resistant genotypes have been found in apricot (*Prunus armeniaca* L.), one of which—the cultivar ‘Lito’ heterozygous for the resistance—has been used to map a major quantitative trait locus (QTL) on linkage group 1, following a pseudo-test-cross mating design with 231 individuals. In addition, 19 SNP markers were selected from among the hundreds previously developed, which allowed the region to be limited to 236 kb on chromosome 1. A ‘Lito’ bacterial artificial chromosome (BAC) library was produced, screened with markers of the region, and positive BAC clones were sequenced. Resistant (R) and susceptible (S) haplotypes were assembled independently. To refine the assembly, the whole genome of ‘Lito’ was sequenced to high coverage (98×) using PacBio technology, enabling the development of a detailed assembly of the region that was able to predict and annotate the genes in the QTL region. The selected cultivar ‘Lito’ allowed not only to discriminate structural variants between the two haplotypic regions but also to distinguish specific allele expression, contributing towards mining the *PPVres* locus. In light of these findings, genes previously indicated (i.e., *MATHd* genes) to have a possible role in PPV resistance were further analyzed, and new candidates were discussed. Although the results are not conclusive, the accurate and independent assembly of R and S haplotypes of ‘Lito’ is a valuable resource to predict and test alternative transcription and regulation mechanisms underpinning PPV resistance.

## Introduction

Sharka is one of the most prominent viral diseases affecting apricot and other stone fruit crops and is caused by a potyvirus (*Plum Pox Virus*, PPV) transmitted by aphid vectors. Pesticides are ineffective against the virus and control of the vector is not fast enough to prevent the spread of the disease in orchards (Rimbaud et al., 2015). Genetic sources of resistance to Sharka have been identified in several apricot cultivars as well as in wild apricots originating in Central Asia (Kegler et al., 1998; Zhebentyayeva et al., 2008; Decroocq et al., 2016). Analysis of these genotypes may suggest possible ways of overcoming the severe impact of Sharka on the apricot industry.

Apricot resistance to Sharka is commonly assayed through phenotypic observations of plants challenged with the virus (Kegler et al., 1998; Moustafa et al., 2001; Soriano et al., 2008). Unfortunately, the phenotypic scoring of resistance is often problematic and conflicting reports and different genetic models of resistance were reported in the early literature (Guillet-Bellanger and Audergon, 2001; Moustafa et al., 2001; Rubio et al., 2007; Soriano et al., 2008; Dondini et al., 2011). The refinement of quantitative trait locus (QTL) analysis made the model based on a major determinant located in the subtelomeric region of chromosome 1 more consistent (Hurtado et al., 2002; Dondini et al., 2011; Vera Ruiz et al., 2011; Soriano et al., 2012). Additionally, weaker QTLs were identified in linkage groups (LGs) 3, 4, and 7 and upstream of the main QTL of LG 1 (Lambert et al., 2007; Marandel et al., 2009), but their role in PPV resistance has not yet been well defined, probably due to the limited size of the populations used or due to the difficulties encountered in phenotyping (Mariette et al., 2016).

Two different mechanisms of resistance have been proposed for potyviruses. The first is based on mutations of the eukaryotic translation initiation factors (*eIF* isoforms), which are proteins necessary for virus replication in the host (Ruffel et al., 2002; Marandel et al., 2009). However, the involvement of these genes would imply a recessive model of resistance (Diaz-Pendon et al., 2004), and this is not consistent with the apricot heritability model, which is dominance-based as indicated by evidence of segregation (Dondini et al., 2011). The second mechanism involves an active resistance response triggered by the host’s recognition of the virus; in this case, the plant response is carried out either by a hypersensitive response and programmed cell death (Neumüller and Hartmann, 2008), or by an RNA interference system allowing for the recognition and targeting of viral nucleic acids (Voinnet, 2005). On the other hand, several virus-encoded proteins are able to suppress the plant RNA silencing machinery, thus protecting virus replication and spread inside the infected tissues (Pumplin and Voinnet, 2013). Recently, a third model of resistance based on the restriction of long-distance virus movement has been reported for several unrelated potyviruses (Cosson et al., 2010), but including *Plum Pox Virus* (Decroocq et al., 2006). This model suggests that Restricted TEV Movement (RTM) proteins, belonging to an undescribed protein family with a meprin and TRAF homology (MATH) domain in its amino-terminal region and a coiled-coil domain at its carboxy-terminal end, might form a multiprotein complex that would block the long-distance movement of potyviruses. In recent studies, the *PPVres* locus was narrowed to a region of approximately 196 kb, and some 68 variants within 23 predicted transcripts were identified according to the peach genome annotation taken as the closest reference genome. Combining the sequence variants and the gene-predicted functions, several authors indicated members of a cluster of meprin and TRAF-C homology domain (MATHd)-containing proteins as the most likely candidates for PPV resistance in apricot (Zuriaga et al., 2013; Zuriaga et al., 2018).

RNA-seq analysis of two full-sib apricot genotypes, ‘Rojo Pasión’ and Z506-7, resistant and susceptible to PPV, respectively, inoculated with a PPV-D strain was carried out by Rubio and co-workers (2015). They found 49 genes in the QTL region of LG 1 potentially involved in the mechanism of resistance, including several MATH domain-containing genes. The genetic diversity of 72 apricot accessions was explored through a genome-wide association study (GWAS) and next-generation genotyping using the peach genome as a reference for the single nucleotide polymorphism (SNP) positions (Mariette et al., 2016). Utilizing the differential response of each cultivar to the PPV-M strain and SNP markers association to the phenotypic observations, the authors listed 10 and 14 genes dealing with the resistance to *PPV* for each of the two regions and theorized that the two candidate genes have an epistatic interaction, as previously suggested on the base of a QTL meta-analysis (Marandel et al., 2009; Decroocq et al., 2014). However, to date, the genetic control of PPV resistance in apricot still remains controversial owing to the lack of a complete and accurate sequencing of the apricot genome and little knowledge of virus–plant interaction mechanisms.

In the present paper, we report improvements to the genetic linkage map of the PPV resistance QTL, which allowed us to confirm and restrict its genetic location. Moreover, a bacterial artificial chromosome (BAC)-based physical map and a genomic assembly of the resistant and susceptible haplotypes of ‘Lito’ chromosome 1 (heterozygous for the PPV resistance) were produced. Finally, based on the phased assembly, we predicted and annotated the genes at the PPV locus and computed allele-specific expression of the resistant and susceptible haplotypes. Candidate genes were identified and discussed with special emphasis on the emerging knowledge on virus resistance mechanisms.

## Materials and Methods

### Plant Material

A pseudo-test-cross apricot (*Prunus armeniaca* L.) F1 population (‘Lito’ × BO81604311), with ‘Lito’ heterozygous for the resistance to PPV and BO81604311 susceptible) previously employed for mapping the resistance to both D and M strains of *PPV* (Dondini et al., 2011) was used. The progeny we used consisted of original L × B and extended L × B crosses, with 118 seedlings and 231 seedlings, respectively.

### Saturation of the Linkage Map of the Region of Interest With New Markers

For the ‘Lito’ parent, a paired-end library was constructed following the ThruPLEX^®^ DNA-seq Quick Protocol, Dual Indexes. Whole-genome sequencing was carried out on an Illumina GAIIx machine in paired-end mode with 100-bp-long reads. Reads were aligned on the peach genome sequence v1.0 with CLC Genomics Workbench (https://www.qiagenbioinformatics.com/products/clc-genomics-workbench/) with similarity 0.92, length fraction 0.9, insertion and deletion cost 3, and mismatch cost 2. SNPs were called with CLC Genomics Workbench with minimum coverage 6, maximum coverage 50, and minimum variant frequency 30%. SNPs and surrounding 300 bp were visually inspected using Tablet (Milne et al., 2010) to detect only sound alignments. Three hundred base pairs of sequence surrounding reliable selected SNPs were used to design primers with MassARRAY Assay Design v3.1 software (Ellis and Ong, 2017) (**Supplementary Table 1**). SNPs were genotyped on the ‘Lito’ × BO81604311 progeny as well as on the BAC pools (see below) by using a Sequenom platform (Gabriel et al., 2009).

The final genetic map was obtained by using a subpopulation of 250 seedlings that included the initial progeny of 118 seedlings and 132 further individuals of the extended cross population (Dondini et al., 2011) that were informative for analysis of the Lito’s LG1 recombinations. The updated map was constructed using Joinmap 4.1 and the Kosambi’s mapping function with a LOD score of 10 for grouping (Van Ooijen, 2006). SNP markers with the segregation type < abxab> were not used in the map construction, but only for screening the BAC library (see below).

Moreover, the UDAp441 and pchcms4 markers, located upstream and downstream of the QTL, respectively, and covering approximately a map distance of 5 cM, were projected onto the peach genome sequence v 1.0 (Verde et al., 2013), and the corresponding peach sequence was extracted. SSR and SCAR markers were identified *in silico* with a modified version of Abajian’s Sputnik software (http://abajian.net/sputnik/index.html, website no longer available; Scalabrin S., personal communication). Unique primers extracted from the peach sequence were designed with Primer software v. 3 (Untergasser et al., 2012) and used for the BAC library screening (**Supplementary Table 2**). Further seven markers were recovered from the literature (Soriano et al., 2012; Zuriaga et al., 2013; Decroocq et al., 2014).

### Phenotypic Analysis of Recombinants

Recombinants detected in the QTL region were grafted onto the ‘GF305’ rootstock and phenotyped for resistance to PPV (M strain, isolate GR0019) according to the protocol previously described (Amenduni et al., 2004; Dondini et al., 2011), using a three-class score: 0 = resistant (no symptoms, ELISA-negative), 1 = tolerant (no symptoms, ELISA-positive), and 2 = susceptible (presence of symptoms, ELISA-positive). Plants were observed for three subsequent growing seasons and those that were ELISA-negative further tested by RT-qPCR (Olmos et al., 2005).

### BAC Library Screening in the PPV Resistance Locus

A BAC library of ‘Lito’ cultivar was commissioned from Lucigen (Middleton WI, USA). Fragments obtained by random shearing were cloned into pSMART BAC Vector (30,336 clones, 110- to 130-kb insert size, approx. 10× coverage). The BAC library was screened with the markers described above (**Supplementary Table 2**) using a tri-dimensional pooling strategy (79 plate pools, 16 row pools, and 24 column pools for a total of 119 pools), followed by a resolution of conflicts. The PCR mix (0.125 U Taq (Thermo Fisher), 2 mM MgCl_2_, 500 nM each SSR or SCAR primer, and 100 µM dNTPs) was amplified in an Applied Biosystems 2720 thermal cycler according to the protocol described in Savazzini et al. (2017).

### BAC Extraction, Digestion, and End Sequencing

BAC clones that provided marker amplification were individually extracted and digested as described in Savazzini et al. (2017). Briefly, *Escherichia coli* was grown overnight on Luria–Bertani (LB) medium supplemented with chloramphenicol, then BAC clones were purified using a modified alkaline lysis protocol.

Twenty micrograms of each colony was digested overnight with the *Eco*RI enzyme at 37°C for BAC restriction analysis. Samples were loaded on 1% agarose gel and run overnight.

Nine microliters (200–300 ng/µl of DNA) was used as template for BAC-end sequencing. The PCR mix contained 1× Sequencing Buffer (Applied Biosystems), BigDye^®^ Terminator v.3.1 (Applied Biosystems) and specific primers of the pSMART BAC vector (SL1: 5′-CAGTCCAGTTACGCTGGAGTC-3′; SR4: 5′-TTGACCATGTTGGTATGATTT-3′). The PCR thermal conditions were 96°C for 10 s, 50°C for 5 s, 60°C for 4 min, for 99 cycles. BAC ends were sequenced using Sanger technology on ABI3730xl sequencers. Resulting sequences were aligned against the peach reference genome using BLAST algorithm (http://services.appliedgenomics.org/blast/prunus/). New primers were developed from the BAC-ends and used to screen again the BAC library to extend contigs. The list of primers is reported in **Supplementary Table 2**.

### Sequencing of Positive BACs

BAC clones representing a minimal tiling path of the region associated to the PPV locus were sequenced. Clones were purified and quantified as previously described. Paired-end libraries were constructed following the ThruPLEX^®^ DNA-seq Quick Protocol Dual Indexes and run on an Illumina MiSeq; mate-pair libraries of resistant and susceptible BAC clone pools were constructed using Nextera Mate Pair Gel-Free Sample Preparation protocol and run on an Illumina Hiseq2000 sequencer. Average reads length was 300 bp.

### BAC Assembly and Ordering Within PPV Resistance Locus

Adapter sequences were trimmed on both pair-end and mate-pair raw reads using cutadapt (Martin, 2011). ERNE-FILTER (Del Fabbro et al., 2013) was used to trim low-quality bases and to filter reads matching either pSMART BAC Vector, *E. coli*, or the *Prunus persica* (peach) chloroplast sequence. Reads from each BAC clone were then assembled separately with CLC Genomics Workbench v.3 with default parameters.

The relative position of the molecular markers developed within the peach genome v.1.0 (www.rosaceae.org) and the LG1 peach sequence v.2.0 from 6.6 to 8.8 Mbp assisted the general ordering of the BAC clones within the putative *PPVres* locus. BAC contigs were aligned to the peach genome v.2.0 using BLAST and Genome evolution analysis CoGe-GEvo (www.genomevolution.org). The peach genome served initially as a good proxy, but contigs order was not solved in apricot regions lacking collinearity with the peach genome. Indeed, in many cases, the BAC contigs alignment against the peach genome provided multiple matches. For this reason, in a second time, the order of the BAC contigs was solved by aligning one BAC clone against each other. Contigs were manually oriented and in some cases fused after dotplot inspection with Dotter (Barson and Griffiths, 2016): overlapping sequences were assembled with iAssembler v1.3.2e (Zheng et al., 2011) by setting the minimum overlap length to 100 bp and the minimum identity to 99%. Mate-pair reads were aligned against the supercontigs and the correct order and orientation were verified by observing whether they aligned at the expected distances and orientation. Gaps (stretches of Ns) were added between adjacent contigs based on the aforementioned alignment on the peach genome. Assemblies were kept separate between the R and S haplotypes.

### ‘Lito’ Whole-Genome Sequencing With PacBio Technology

A total of 20 g of young ‘Lito’ leaf tissue was provided to Amplicon Express (Pullman, WA, USA) for sequencing using the Single Molecule Real-Time (SMRT) technology of Pacific Biosciences (PacBio). Briefly, high-molecular-weight DNA was extracted using the CTAB isolation method of Doyle and Doyle modified by R. Meilan (unpublished, http://ampliconexpress.com/ngs-grade-dna-extraction-services/). Eleven SMRTcell libraries were constructed with the SMRTcell™ Template Prep Kit 1.0 following the manufacturer’s instructions using 5 µg of DNA. Fragments <2 kb were removed and the size range of the libraries validated by the Agilent 2100 Bioanalyzer. Six libraries were sequenced using the PacBio^®^ RS II System and an additional five using the Sequel™ System.

### Assembly of Susceptible and Resistant Haplotypic Regions of ‘Lito’

BAC assemblies were evaluated and optionally extended either by aligning the PacBio reads to BAC supercontigs or by comparing supercontigs to an independent assembly of PacBio reads.

Alignment of the PacBio reads against the combined BAC assembly of the region for the two R and S haplotypes was carried out with BLASR (https://github.com/PacificBiosciences/blasr) with -bestn 1. In order to validate possible joins between adjacent BAC supercontigs, the genome viewer IGV3 (http://software.broadinstitute.org/software/igv/igv3.0) was used to visualize and validate PacBio reads alignments.

PacBio reads of ‘Lito’ were independently assembled using Canu software (Koren et al., 2017), setting corrected ErrorRate = 0.045 and corMax EvidenceErate = 0.2. Statistics for the assembly were obtained using QUAST (http://quast.sourceforge.net/quast). 

Canu contigs covering the region of interest for the PPV resistance were chosen from the *de novo* assembly of ‘Lito’ using the Basic Local Alignment Search Tool (ftp://ftp.ncbi.nlm.nih.gov/blast/executables/blast+) which finds regions of local similarity between sequences. This program allowed to compare nucleotide sequences from BAC supercontig assembly with the *de novo* contig sequences obtained with Canu software. The e-value was set to 1e−200. This parameter gives a measure of the similarity of sequences: lower e-values indicate higher congruity between query and retrieved sequence. Selected contig sequences were first aligned against BAC supercontigs using NUCMer (NUCleotide MUMer, http://mummer.sourceforge.net/) tool, setting the program options –mum, −l 30, −c 100. This permitted to assign the contigs, at macro – level, to the susceptible and resistant haplotypes. Canu split haplotypes into separate contigs wherever the allelic divergence is greater than the post-correction overlap error rate. As a result, two mostly redundant contigs covering the same region were obtained. The haplotype with more reads was often reconstructed in a large contig spanning the locus, while the haplotype with fewer reads was just the variant. Less diverged regions were collapsed (**Supplementary Figure 4A**). At a later time, contigs were aligned against BAC supercontigs using Dotter (Barson and Griffiths, 2016) which permitted to verify the concordance between the nucleotide sequences, to verify the contig order inside the BAC supercontigs, to close gaps, and, where possible, to extend the assembly scaffolding a unique sequence (**Supplementary Figure 4B**). Finally, the BAC Illumina reads were aligned against the Canu assembly using the Burrow–Wheeler Aligner (Li and Durbin, 2009) to verify the accuracy of the sequences and to correct possible errors. SNPs and small INDELs were called using default parameters of the Unified Genotyper of GATK (McKenna et al., 2010), manually checked and corrected with seqtk (https://github.com/lh3/seqtk). 

The assembled sequences of the resistant haplotype were aligned against those of the susceptible haplotype to help visual comparisons of the two haplotypes using NUCMer (NUCleotide MUMer, http://mummer.sourceforge.net/) tool, setting the program options –mum, −l 30, −c 100. The same analysis was performed aligning the assembled sequences for both R and S ‘Lito’ haplotypes onto the recently released peach genome v.2.0 (www.rosaceae.org/species/prunus_persica/genome_v2.0.a1). 

A reference ‘Lito’ whole genome was created by joining all ‘Lito’ contigs assembled by Canu in a multifasta file. The contigs covering the LG1 region of interest were removed from this file and replaced with the BAC assembled and curated sequences.

### Gene Prediction and Annotation of ‘Lito’ Whole Genome

The software MAKER (http://www.yandell-lab.org/software/maker.html) was used to predict genes on the assemblies of ‘Lito’ whole genome. Input information for MAKER was represented by:

- SwissProt data files containing protein sequences from *Prunus*, *Arabidopsis*, *Solanum*, and *Nicotiana* species obtained from UniProt (www.uniprot.org);
- EST sequences of the same species extracted from GenBank and downloaded from the NCBI EST database (www.ncbi.nlm.nih.gov);
- CDS sequence files of *Pinus sibirica* and *Prunus mandshurica*, two species closely related to apricot available in NCBI resources (www.ncbi.nlm.nih.gov);
- Files of repetitive elements, primary transcripts, predicted gene transcripts, and predicted gene peptides of *P. persica* obtained from the Peach Genome Browser of the Institute of Applied Genomics (http://services.appliedgenomics.org/projects/prunus_persica_v2/prunus_persica_v2/gbrowse/index.html);
- Three RNA-seq datasets from different *P. armeniaca* tissues (SRX1186946, SRX1186893, SRX2538214), and two RNA-seq data sets from different *Prunus mume* (DRX012518, SRX1187101), the Japanese apricot, downloaded from the NCBI Sequence Read Archive.

RNA-seq data files were quality trimmed with ERNE-FILTER (Del Fabbro et al., 2013) with default parameters.

’Lito’ whole genome reference file was indexed with Bowtie2 (Langmead and Salzberg, 2012). Reads from each RNA-seq data file were aligned against the reference file using TopHat (Kim et al., 2013). Individual transcripts were assembled and quantified from RNA-seq reads using Cufflinks (Trapnell et al., 2010). One of the files produced by Cufflinks, transcripts.gtf, after conversion in gff3 format, was passed as input to MAKER.

General criteria used to annotate the genes are described in the MAKER software (Cantarel et al., 2007). MAKER also provided support for functional annotation (i.e., for identifying the putative gene function) using a protocol based on NCBI BLAST+ and the well-curated UniProt/Swiss-Prot set of proteins to assign putative function to newly annotated genes.

### Manual Curation of Gene Models and Allele-Specific Expression in the PPV Resistance Region

The gene prediction from MAKER, in the restricted Sharka QTL region, was manually refined exploiting RNA-seq data from three different cultivars (Zuriaga et al., 2018): ‘Stella’ homozygous for *PPV* resistance, ‘Canino’ homozygous for *PPV* susceptibility, and ‘Goldrich’ heterozygous and *PPV*-resistant. Reads were quality trimmed with ERNE-FILTER (Del Fabbro et al., 2013) with default parameters. Trimmed reads were aligned with STAR (Dobin et al., 2013) using option ’–quantMode GeneCounts’. Aligned reads were counted with HTSeq-count (Lykke-Andersen and Jensen, 2015). After visual inspection of alignments on IGV3 (http://software.broadinstitute.org/software/igv/igv3.0), some exons were modified in order to avoid stop codons, elongating coding sequences and untranslated regions wherever possible and splitting adjacent genes when fused by the automatic gene prediction.

BLAST algorithm was used to align predicted genes to relate nucleotide sequences from other organisms and to validate their prediction and functionality.

For the curated 48 genes at the PPV locus, a Log_2_ ratio was computed for the read counted in the haplotype S of ‘Canino’ and those counted in the haplotype R of ‘Stella’ (ChrS/ChrR), provided that both cultivars were homozygous in the QTL region. A threshold of 1 or −1 was set to consider genes with doubled or halved coverage.

Both curated coding sequences (CDSs) and related encoded proteins from the R haplotype were aligned and compared with their homologs in the S haplotype using the pairwise sequence alignment tool EMBOSS Needle (https://www.ebi.ac.uk/Tools/psa/emboss_needle/) with default parameters.

### SNP Calling and Filtering

A SNP survey was carried out in the region of the *PPVres* (1,131,617–1,399,894 bp of the ‘Lito’ R sequence and 1,135,005–1,412,063 bp of the ‘Lito’ S sequence) by comparing the apricot cultivars ‘Lito’ (resistant, heterozygous at the *PPVres*), ‘Stella’ (resistant and reported as homozygous at the *PPVres*), ‘Canino’ (reported as homozygous for PPV susceptibility), and ‘Goldrich (resistant and reported heterozygous at the *PPVres*). Information about the cultivars other than ‘Lito’ are from Zuriaga et al. (2018).

SNPs were called according to GATK best practices for RNA-seq data (https://software.broadinstitute.org/gatk/), including filtering aligned reads by mapping quality 10, removing duplicated reads with MarkDuplicates, and splitting reads aligned over introns with SplitNtrim. SNPs were called using GATK HaplotypeCaller version 4.0.10.0 with parameters - dont-use-soft-clipped-bases -stand-call-conf 20.0. SNPs were selected and filtered by QD < 2.0 or FS > 30.0. Finally, a position was called when at least two cultivars were covered by eight or more reads and the minimum allele frequency (MAF) to call a site heterozygote in a cultivar ≥0.25.

## Results

### Saturation of the Linkage Map and Analysis of Recombinants

**Figure 1:**
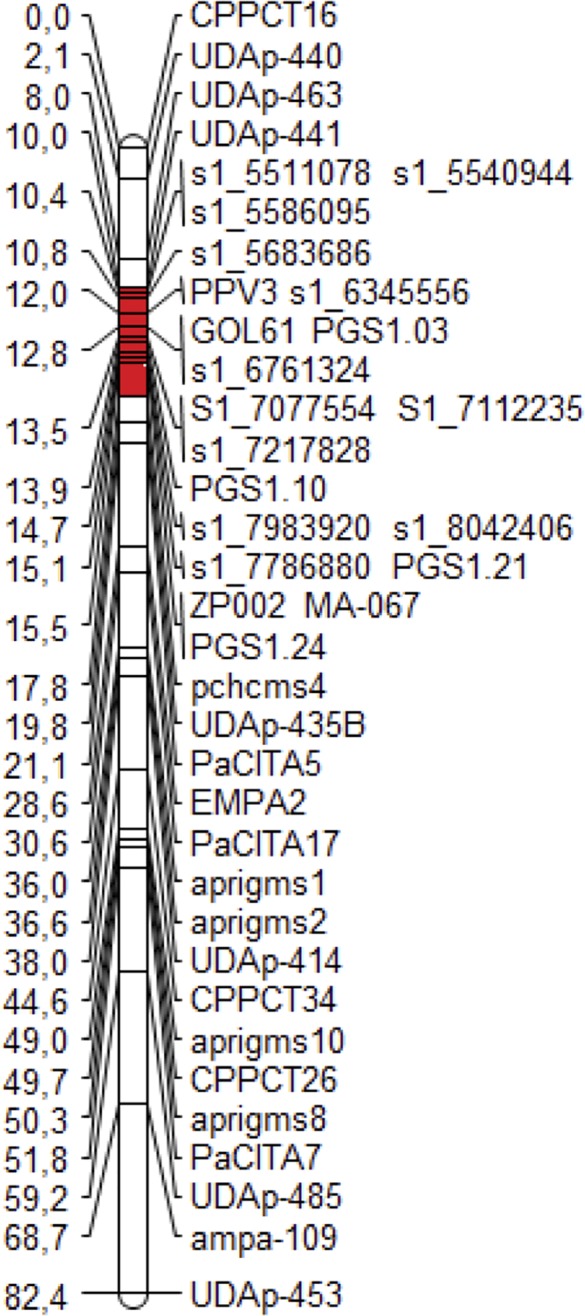
The new map of the ‘Lito’ resistant cross parent obtained with the additional markers developed in the present work. The region harboring the resistance to Sharka spanning from the marker UDAp-441 to the marker pchcms4 is marked in red.

The ‘Lito’ Illumina whole-genome sequencing produced 39.37× coverage (based on the peach genome size), of which 18.67× aligned on the peach genome v1.0. From the alignment, 2,780,615 SNPs were identified. Nineteen new markers, 12 SNPs from ‘Lito’ and seven markers from the literature, were positioned in the new map of ‘Lito’ linkage group 1 (LG1) (**Figure 1**).

**Figure 2:**
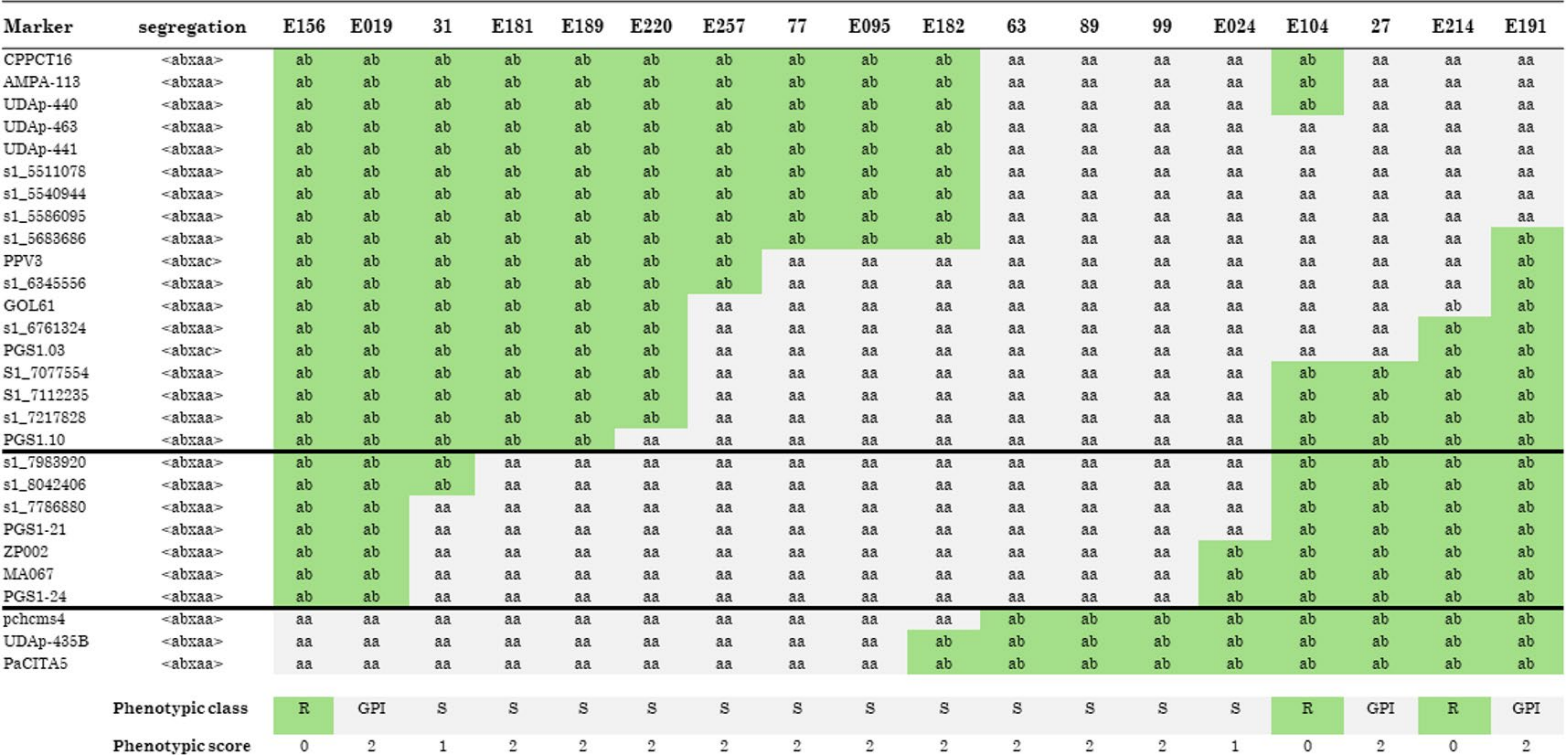
Recombinants in the “putative” region of resistance to Sharka. Molecular markers (*in rows*) are analyzed in all recombinants (*in columns*). Alleles associated with the resistant haplotype are scored as *R* in a *gray background* and those carrying the allele associated with the susceptible haplotype are scored as *S* in a *white background*. GPI indicates a genotype–phenotype incongruence. The phenotypic classes have been scored as follows: R (resistant, score 0), S (susceptible, score 2), and tolerant (score 1). Recombinants phenotypically scored as tolerant were not considered in the analysis. *Black horizontal lines*, between the marker s1_7983920 and PGS1-24 included, restrict the area of the QTL. See the text for details.

The increase in marker density allowed for the identification of 18 recombinants that were grafted onto GF305 and challenged with the PPV for 2 years. The analysis of recombinants (**Figure 2**) was carried out in a conservative way: only individuals having a phenotypic score of 0 (resistant) and 2 (susceptible) were considered, while two recombinants (31 and E024) with a phenotypic score of 1 (tolerant) were not considered in this analysis. This allowed us to restrict the region of interest between the markers S1_7983920 (top) and PGS1.24 (bottom), corresponding to the interval 8,433,190–8,668,808 bp (236 kb) on chromosome 1 of peach genome v 2.0 (**Figure 2** and **Supplementary Table 2**). In that region, 10 recombinants carried the susceptible allele (S) of ‘Lito’ and were phenotypically susceptible, and six recombinants carried the resistant allele (R) of ‘Lito’. However, among these, three were scored as phenotypically resistant and the other three were susceptible (GPI).

### The BAC-Based Physical Map in the Major PPV Resistance Locus

Approximately 100 BAC clones that were positive at the marker’s amplification were assigned to either the resistant (R) or the susceptible (S) haplotype of ‘Lito’ according to the coupling/repulsion phase of alleles. Of these, a set of minimum-tiling path clones (25 and 28 for resistant and susceptible haplotypes, respectively) was then selected for whole sequencing (**Supplementary Table 3**).

The two-tier assembly, first BAC by BAC and then merging overlapping BAC sequences, allowed us to reconstruct five ordered and oriented supercontigs for the resistant haplotype and six for the susceptible one, encompassing 1.4 and 1.6 Mb, respectively. Gaps between adjacent supercontigs were filled with 500 Ns.

The sequences were not complete, being hampered either by the presence of repetitive DNA or by the lack of perfect collinearity of apricot with the peach genome (**Supplementary Figures 1** and **2**). Both reasons hampered the design of new specific primers on the supercontig ends, which could have permitted the identification of further BAC clones to fill gaps, and suggested that a different approach was required.

### Pacbio Sequencing and Refinement of the Assembly of the Region of Interest

The ‘Lito’ genome was sequenced with PacBio technology, which produces long sequences to enable gap filling. The high error rate of PacBio technology (10–15% for a single read) was counteracted by the use of the more reliable Illumina reads of the BAC clones. Three rounds of PacBio sequencing produced a total of 3,011,677 reads, corresponding to a coverage of 98×, considering the estimated apricot genome size of 240 Mb (https://www.rosaceae.org/organism/Prunus/armeniaca). Relevant metrics of PacBio sequencing are reported in **Supplementary Table 3**.

**Figure 3:**
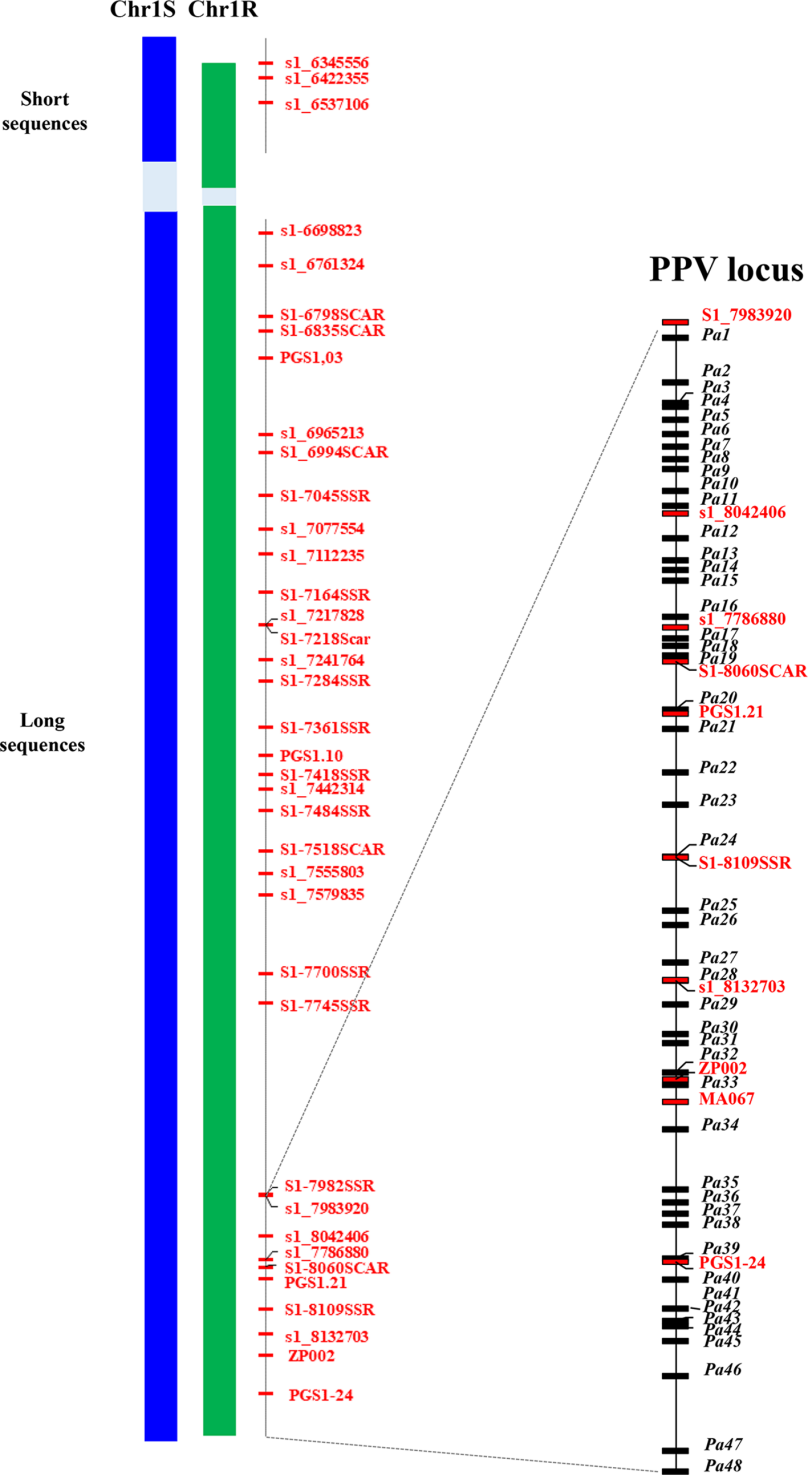
Schematic representation of the assembled haplotypic regions of the susceptible (Chr1S) and resistant (Chr1R) chromosome 1 of ‘Lito’ apricot flanked by the position of markers in the peach genome v 2.0. Both regions are split into two sequences (short and long) separated by a gap (in light blue background color) of unknown length containing repeated motifs that hint the assembly in the gap. Markers (in red) position corresponds to the relative position of the forward primer in the assembly. Markers coded as s1_n are SNP markers and n is their absolute position in the peach sequence v 1.0 (the last three digits are missing). Markers ending with SSR or SCAR are microsatellite or SCAR markers. On the right, the delimited region of the PPV locus. Markers (*in red*) and the annotated genes (in black) are reported with their relative position within the region.

The projection of PacBio contigs to the Illumina BAC assemblies allowed us to a) extract the PacBio sequences of the region of interest; b) refine the separation of R and S haplotypes of PacBio sequences; and c) correct the errors in the PacBio sequences. As a result, for each haplotype of the PPV resistance region, two supercontigs were obtained. The R haplotype included two sequences: one short sequence (Rshort) of 264,556 bp and a long one (Rlong) of 1,419,143 bp, the two sequences totalling 1,683,699 bp. The S haplotype included two sequences as well: the short one (Sshort) of 321,950 bp and the long one (Slong) of 1,413,486 bp, the two totalling 1,735,436 bp. In both haplotypes, a gap made up of repetitive sequences was left; the size of the gap could not be estimated because of the lack of collinearity with the peach sequence. Thirty-eight and 39 markers found their position in the sequence of the R and S haplotypes, respectively (**Figure 3**).

### Comparison of Peach and Apricot Genomes in the Region of Resistance

The QTL region of ‘Lito’ aligns on the peach chromosome 1 between 6.60 and 8.85 Mbp (Verde et al., 2017).

The comparison of sequences confirms a large macro-collinearity between the two species, with a similarity at sequence level ranging between 80% and 95%. However, this appreciable similarity did not appear sufficient to allow a reliable apricot fine genome assembly based uniquely on the peach genome sequence.

Indeed, some BAC contigs could not be aligned uniquely on the peach genome. In particular, a gap between 7.6 and 8.0 Mbp in the peach genome could be covered in apricot only by assembling the ‘Lito’ sequences disregarding the peach sequence. Moreover, a large inversion of 12,691 bp was found in apricot compared to the peach position 8,248,039–8,260,730 bp (**Supplementary Figure 2**).

### Differences Between the ‘Lito’ Resistant and Susceptible Haplotypes

According to the results of the NUCMer tool (NUCleotide MUMer, http://mummer.sourceforge.net/), the two haplotypes share a similarity close to 100%, with some insertions within the sequences of the resistant haplotype that are missing in the susceptible haplotype and vice versa (**Supplementary Figure 3**). A sequence of 11,558 bp in the susceptible contig Sshort is apparently duplicated in the resistant one (Rshort) in positions 221,476 and 241,881, respectively (**Supplementary Figure 3**).

### Gene Prediction and Annotation in the PPV Resistance Locus

MAKER predicted 388 genes in the region of the major QTL for PPV resistance in ‘Lito’: 199 genes in the resistant haplotype and 189 in the susceptible haplotype. We obtained a cumulative distribution of the annotated transcripts based on the Annotation Edit Distance (AED) score, a concise way of showing the quality of the annotated transcripts based on the relative distance to the information passed in input (e.g., this value is lower and the distance is smaller). The curve shows that approximately 80% of the annotations have AED scores lower than 0.4, i.e., 310 of annotated genes are highly supported by the evidence considered to make the prediction (**Supplementary Figure 5**).

The list of genes and relative annotation is reported in **Supplementary Table 4**.

### Allele-Specific Expression of Annotated Genes in PPV Locus

The 236-kb restricted region of PPV resistance was found to contain 48 predicted and annotated genes. In **Table 1**, all transcripts were identified by the ’*Pa*’ tag followed by a progressive number.

**Table 1:** List of the 48 genes predicted and annotated in the region of the QTL of resistance to PPV, separated for each haplotype R (resistant) and S (susceptible) of ‘Lito’.

Tag identifier	Susceptible haplotype			Gene function	Resistant haplotype			R vs. S		
	Start	End	Gene ID		Gene ID	Start	End	% Identity transcripts	% Identity proteins	Differential expression
Pa1	1135005	1136653	Par.chr1S_long.5.83	Late embryogenesis abundant protein D-29	Par.chr1R_long.5.91	1131617	1133432	100.0	100	
Pa2	1145892	1150557	Par.chr1S_long.5.84	CBL-interacting serine/threonine-protein kinase 1 isoform X1	Par.chr1R_long.5.117	1140929	1145650	99.9	100	>R
Pa3				Hypothetical protein Pyn_20424	Par.chr1R_long.5.107	1145953	1146486			
Pa4	1151829	1154369	Par.chr1S_long.5.32	Probable GTP diphosphokinase CRSH, chloroplastic	Par.chr1R_long.5.108	1146968	1149422	98.2	97.40	>R
Pa5	1155002	1158187	Par.chr1S_long.5.86_A	50S ribosomal protein L3-2, chloroplastic	Par.chr1R_long.5.95_A	1150023	1153228	98.8	98.50	>R
Pa6	1158480	1159884	Par.chr1S_long.5.86_B	Putative transcription factor C2H2 family	Par.chr1R_long.5.95_B	1153526	1154832	99.4	100	>R
Pa7	1161539	1163509	Par.chr1S_long.5.98	Pectinesterase-like	Par.chr1R_long.5.109	1156572	1158542	99.6	100	
**Pa8**	**1164587**	**1166585**	**Par.chr1S_long.5.87_A**	**Pentatricopeptide repeat-containing protein At5g66520-like**	**Par.chr1R_long.5.96_A**	**1159661**	**1161684**	**99.6**	**99.20**	**>R**
Pa9	1167012	1171766	Par.chr1S_long.5.87_B	Putative transcription factor/chromatin remodeling BED-type(Zn) family	Par.chr1R_long.5.96_B	1162056	1166802	99.6	74.80	>R
Pa10	1172307	1174945	Par.chr1S_long.5.87_C	rRNA-processing protein fcf2-like	Par.chr1R_long.5.96_C	1167278	1170007	97.1	97.50	>R
**Pa11**	**1175932**	**1179241**	**Par.chr1S_long.5.99**	**Pyruvate dehydrogenase (acetyl-transferring) kinase, mitochondrial**	**Par.chr1R_long.5.110**	**1170443**	**1173780**	**99.5**	**99.70**	**>R**
Pa12	1183833	1188661	Par.chr1S_long.5.88	Novel plant SNARE 13	Par.chr1R_long.5.97	1178351	1183193	100.0	100	>S
Pa13	1189215	1190729	Par.chr1S_long.6.134	Structure-specific endonuclease subunit SLX1	Par.chr1R_long.5.98	1183756	1185569	97.0	Early stop codon in R	>R
Pa14	1191566	1192624	Par.chr1S_long.6.11	NAC domain-containing protein 83-like	Par.chr1R_long.5.38	1186082	1187140	99.6	100	
Pa15	1194184	1202013	Par.chr1S_long.6.115_A	SWI/SNF-related matrix-associated actin-dependent regulator of chromatin subfamily A-like protein 1 isoform X1	Par.chr1R_long.6.110_A	1188993	1196536	99.7	99.90	>S
Pa16	1202950	1204177	Par.chr1S_long.6.115_B	Cytidine/deoxycytidylate deaminase family protein	Par.chr1R_long.6.110_B	1197681	1198907	99.7	99.20	
Pa17	1208240	1209689	Par.chr1S_long.6.13	Structure-specific endonuclease subunit SLX1	Par.chr1R_long.6.6	1202864	1204313	99.8	99.50	
Pa18	1210055	1211113	Par.chr1S_long.6.28	NAC domain-containing protein 83-like	Par.chr1R_long.6.19	1204679	1205737	100.0	100	
Pa19	1212408	1217246	Par.chr1S_long.6.116	Probable sugar phosphate/phosphate translocator At3g17430	Par.chr1R_long.6.111	1207468	1212275	100.0	100	
**Pa20**	**1225551**	**1229790**	**Par.chr1S_long.6.105**	**Probable receptor-like protein kinase At5g18500**	**Par.chr1R_long.6.99**	**1220979**	**1223864**	**100.0**	**100**	**>S**
**Pa21**	**1230285**	**1234174**	**Par.chr1S_long.6.30**	**Pto-interacting protein 1-like**	**Par.chr1R_long.6.21**	**1226262**	**1227800**	**46,6%**	**Early stop codon in R**	**>S**
Pa22	1240861	1248025	Par.chr1S_long.6.107_A	Squamosa promoter-binding-like protein 12	Par.chr1R_long.6.101_A	1234639	1241977	100.0	100	
**Pa23**	**1248728**	**1255029**	**Par.chr1S_long.6.107_B**	**Squamosa promoter-binding-like protein 1**	**Par.chr1R_long.6.101_B**	**1242656**	**1249045**	**99.2**	**99.10**	**>R**
Pa24	1261425	1263177	Par.chr1S_long.6.31	2-Keto-3-deoxy--rhamnonate aldolase-like	Par.chr1R_long.6.22	1255377	1257129	99.5	99.20	
**Pa25**	**1274528**	**1276849**	**Par.chr1S_long.6.108**	**Pentatricopeptide repeat-containing protein At3g06430, chloroplastic**	**Par.chr1R_long.6.102**	**1261520**	**1263456**	**99.7**	**99.60**	**>S**
Pa26	1278010	1285801	Par.chr1S_long.6.109	Pleiotropic drug resistance protein 3-like	Par.chr1R_long.6.103	1264938	1272745	97.6	97.60	
Pa27	1287184	1294523	Par.chr1S_long.6.119	Pleiotropic drug resistance protein 3-like	Par.chr1R_long.6.113_A	1274263	1277959	51.3	Non-functional protein in R	>S
Pa28					Par.chr1R_long.6.113_B	1278552	1281482	36.7	Non-functional protein in R	
Pa29	1297365	1299476	Par.chr1S_long.6.153	S-adenosylmethionine synthase 2	Par.chr1R_long.6.24	1284329	1286364	98.8	100	>R
Pa30	1304574	1306456	Par.chr1S_long.6.110	Autophagy-related protein 8i	Par.chr1R_long.6.104	1293233	1295137	97.4	100	>R
**Pa31**	**1306781**	**1308738**	**Par.chr1S_long.6.121_A**	**Probable inactive serine/threonine-protein kinase fnkC - MATH**	**Par.chr1R_long.6.147**	**1295506**	**1297481**	**91.6**	**84.30**	**>R**
**Pa32**	**1313963**	**1316024**	**Par.chr1S_long.6.121_B**	**BTB/POZ and MATH domain-containing protein 3-like**	**Par.chr1R_long.6.150_A**	**1306184**	**1308286**	**95.6**	**Early stop codon in R**	**>S**
**Pa33**	**1316981**	**1318698**	**Par.chr1S_long.6.121_C**	**BTB/POZ and MATH domain-containing protein 3-like**	**Par.chr1R_long.6.150_B**	**1309312**	**1311005**	**95.3**	**93.60**	**>R**
Pa34	1327813	1331483	Par.chr1S_long.6.21	Probable inactive serine/threonine-protein kinase fnkC - MATH	Par.chr1R_long.6.135	1313970	1317385	98.3	Early stop codon in S	>R
Pa35	1342468	1343741	Par.chr1S_long.6.157_A	MATH domain and coiled-coil domain-containing protein At3g58210	Par.chr1R_long.6.149_A	1327019	1328292	98.8	Non-functional both in R and S	
Pa36	1345619	1347629	Par.chr1S_long.6.157_B	MATH domain and coiled-coil domain-containing protein At3g58210	Par.chr1R_long.6.149_B	1330217	1332179	99.4	98.70	>R
Pa37	1348313	1349955	Par.chr1S_long.6.39	Proteasome assembly chaperone 4	Par.chr1R_long.6.31	1333386	1334506	99.8	99.40	>S
Pa38	1351004	1358411	Par.chr1S_long.6.40	Protein GFS12	Par.chr1R_long.6.117	1335414	1342967	97.2	97.20	>R
Pa39	1359272	1362511	Par.chr1S_long.6.161	ATP-citrate synthase alpha chain protein 2	Par.chr1R_long.6.152	1343793	1347071	100.0	100	>R
Pa40	1364384	1365469	Par.chr1S_long.6.22	NAC domain-containing protein 83-like	Par.chr1R_long.6.14	1350553	1351611	89.9	88.40	
Pa41	1371430	1372823	Par.chr1S_long.6.42	Zinc finger MYM-type protein 1-like						
Pa42				Structure-specific endonuclease subunit SLX1	Par.chr1R_long.6.34	1352210	1353323			
Pa43	1374487	1375678	Par.chr1S_long.6.96	Mediator of RNA polymerase II transcription subunit 21-like	Par.chr1R_long.6.120	1361959	1363418	61.5	Early stop codon in R	>R
Pa44	1375746	1377783	Par.chr1S_long.6.112	Methyltransferase-like protein 13	Par.chr1R_long.6.106	1363435	1365489	95.8	95.70	>R
Pa45	1379384	1387440	Par.chr1S_long.6.146	Calmodulin-binding transcription activator 3-like isoform X1	Par.chr1R_long.6.137	1367360	1375234	99.9	99.90	>R
Pa46	1387896	1399728	Par.chr1S_long.6.131	Kinesin-like protein KIN-12C	Par.chr1R_long.6.121	1375791	1387524	99.4	Early stop codon in S	>R
Pa47	1406092	1407285	Par.chr1S_long.6.165	GUB_WAK_bind domain-containing protein/WAK_assoc domain-containing protein	Par.chr1R_long.6.158	1393895	1395089	98.9	98.80	
Pa48	1411138	1412063	Par.chr1S_long.6.133	1-Cys peroxiredoxin	Par.chr1R_long.6.124	1398969	1399894	99.7	99.50	

**Figure 4:**
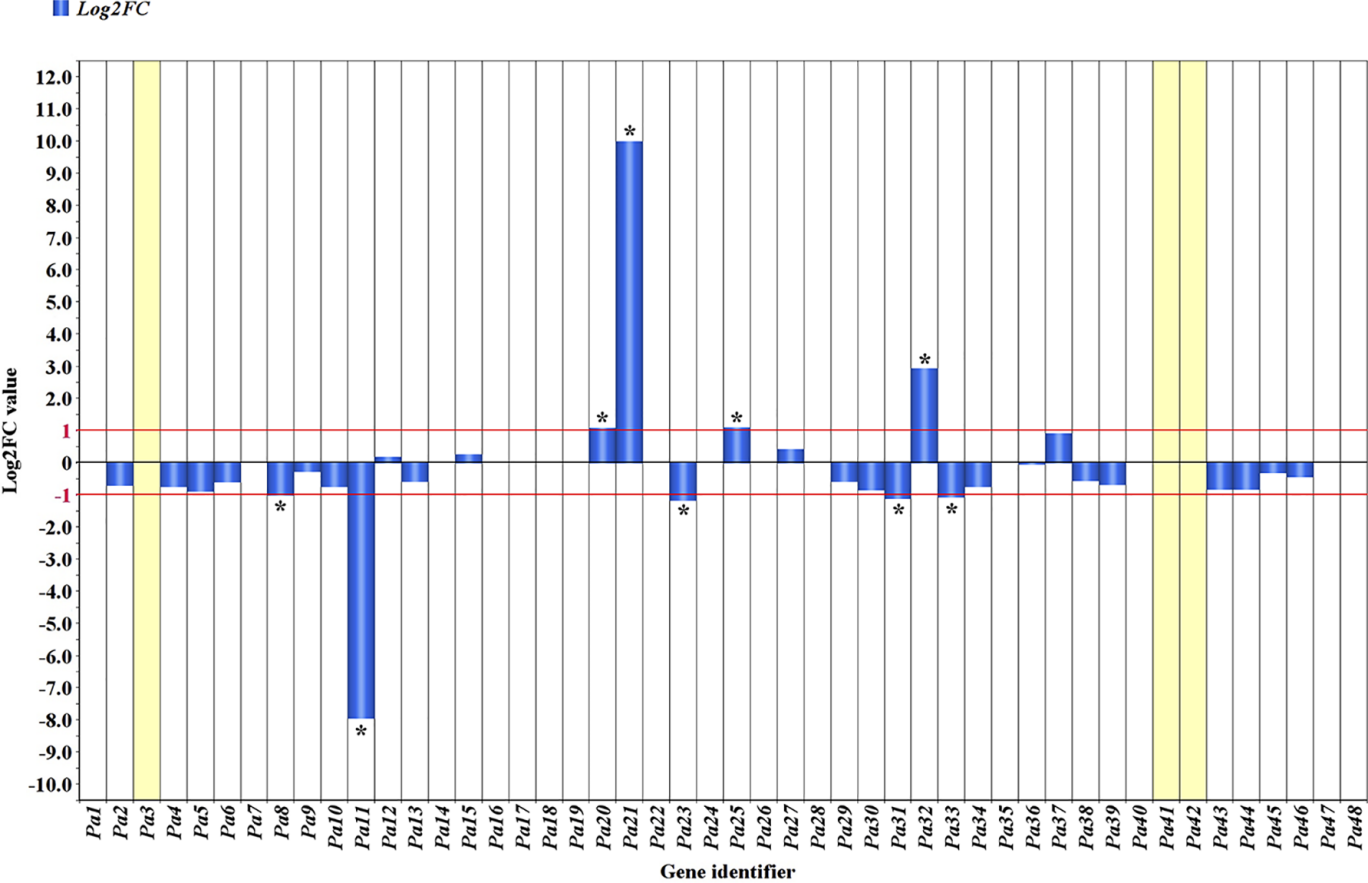
Allele-specific expression in the PPV resistance region. Blue bars indicate the absolute Log_2_ ratio of reads counted in the haplotype S of ‘Canino’ and those counted in the haplotype R of ‘Stella’ (ChrS/ChrR). A threshold of 1 or −1 was set to consider genes differentially expressed. *Genes significantly outside threshold limits. Missing bars generally indicate equal expression in both haplotypes, except for *Pa3*, *Pa41*, and *Pa42*, which have been detected and annotated only in one of the two haplotypes (see **Table 1**).

The comparative analysis of RNA-seq reads of the R and S haplotypes as split by the ‘Lito’ assembly by haplotypes allowed determination of the log_2_-transformed ratio of Chr_S/Chr_R. The range −1 to 1 was chosen as thresholds for defining differential expression (**Figure 4** and **Table 1**) and was exceeded in only a few cases.

Therefore, among the genes exclusively expressed in the R haplotype, we found the hypothetical protein Pyn_20424 (*Pa3*) and the structure-specific endonuclease subunit SLX1 (*Pa42*) exhibiting expression levels comparable to those of other genes. Five genes (*Pa8*, *Pa11*, *Pa23*, *Pa31*, and *Pa33*) encoding a pentatricopeptide repeat-containing protein At5g66520-like, a mitochondrial pyruvate dehydrogenase (acetyl-transferring) kinase, a squamosa promoter-binding-like protein 1, and two MATH domain-containing proteins, respectively, were preferentially expressed in the R haplotype.

Among the genes exclusively expressed in the S haplotype, we found *Pa20*, encoding a probable receptor-like protein kinase At5g18500, *Pa21*, encoding a Pto-interacting protein 1-like, *Pa25*, encoding a pentatricopeptide repeat-containing protein, and *Pa32*, a transcript related to another MATH domain-containing protein (**Figure 4** and **Table 1**).

### Structural Analysis of Coding Allelic Variants in PPV Locus

Several predicted transcripts exhibited structural differences possibly affecting protein stability/functionality (**Table 1** and **Supplementary Figure 6**). Among the genes highly expressed in R and with consistent mutation in the S haplotype, we pointed out that the probable inactive serine/threonine protein kinase fnkC–MATH (*Pa31*), the protein GFS12 (*Pa38*) and a methyltransferase-like protein 13 (*Pa44*) showed deletions in the inner part of the sequence. On the other hand, kinesin-like protein KIN-12C (*Pa46*) and a probable inactive serine/threonine protein kinase fnkC–MATH (*Pa34*) displayed mutations leading to premature stop codon.

The Pto-interacting protein 1-like (*Pa21*) and the MATH domain-containing protein 3-like (*Pa32*), predominantly expressed in the S haplotype, displayed premature stop codons in the R haplotype. Similarly, the pleiotropic drug resistance protein 3-like (*Pa27*-*Pa28*) encoding for a functional protein in S displayed in R haplotype an indel resulting in a truncated and split protein.

Mutations causing a probable loss of function in the R haplotype were also detected in the putative transcription factor/chromatin remodeling BED-type(Zn) family (*Pa9*), in the structure-specific endonuclease subunit SLX1 (*Pa13*), and in the mediator of RNA polymerase II transcription subunit 21-like (*Pa43*); two minor deletions were found in the BTB/POZ and MATH domain-containing protein 3-like (*Pa33*) of the R haplotype. However, in these cases, structural variations occurring in R appeared inconsistent with the higher expression in the same haplotype.

### Analysis of SNP and Their Association With Candidate Genes

As much as 128 SNPs were retrieved from the comparison of the R and S ‘Lito’ haplotypic sequence and the three cultivar ‘Stella’, ‘Canino’, and ‘Goldrich’ at the *PPVres* region (**Supplementary Table 5**).

The positions heterozygous in ‘Lito’ and falling into CDS of the genes of the region resulted in 29 SNPs, several of which fell into the CDS of the candidate genes *Pa31* and *Pa33*. ‘Stella’ was confirmed to be homozygous for the R haplotype of ‘Lito’, whereas ‘Canino’ displayed a great number of heterozygous SNPs.

## Discussion

Despite the high degree of similarity found between peach, *P. armeniaca*, *P. mume*, and *P. sibirica* (over 93%) (Zuriaga et al., 2013), apricot and peach genomes were not strictly collinear in the region of the major determinant of resistance to PPV due to several indels and a sequence inversion. This suggested that the peach genome should not be used as a reference for this work and that a *de novo* assembly of the apricot genome should be implemented both by targeting BAC clones of the region of interest and by sequencing the whole apricot genome with the PacBio technology.

We accurately reconstructed the main QTL for Sharka resistance on apricot LG1 for the resistant (R) and susceptible (S) haplotypes, independently. We annotated the entire assembled region, but we focused on the analysis of the genes between and near the markers s1_7983920 and PGS1-24 of the sequence (PPV locus). This is because, according to the phenotypic evaluation of the recombinants and previous publications (Dondini et al., 2011; Zuriaga et al., 2013; Rubio et al., 2014; Mariette et al., 2016), in this region, at least one of the major determinants of Sharka resistance is documented. Forty-eight genes were predicted and annotated in the region. The analysis of allele-specific expression of R and S haplotypes allowed identifying seven genes exclusively or preferentially expressed in the R haplotype and four genes more highly expressed in the S haplotype. However, purely focusing on the differential expression may have led to missed candidate genes if the phenotypic differences were associated with structural polymorphisms of the genes (i.e., gain or loss of protein function). For this reason, we also analyzed the structural variations occurring in the CDSs.

A cross-talk between transcription and translation is well recognized and nonsense-mediated mRNA decay (NMD), the mechanism that prevents the accumulation of potentially harmful or useless truncated proteins targeting for degradation aberrant mRNAs (Lykke-Andersen and Jensen, 2015). NMD has been recently mentioned also in studies on PPV resistance (Zuriaga et al., 2018), and our data would support the hypothesis that both transcription and posttranscriptional regulation might underpin resistance mechanisms.

Several genes identified in this study as candidates were discussed in the recent literature (Decroocq et al., 2006; Rubio et al., 2015; Mariette et al., 2016; Zuriaga et al., 2018). Meprin and TRAF-C homology domain (MATHd)-containing proteins, also named RTM3-like proteins, are encoded by a class of genes already described as atypical resistance genes that restrict the long-distance movement of several potyviruses in *Arabidopsis thaliana* (Martin et al., 2003; Cosson et al., 2012). In these cases, viral replication and cell-to-cell movement in inoculated leaves appear unaffected, hypersensitive response (HR) and systemic acquired resistance (SAR) are not triggered, and salicylic acid is not involved (Cosson et al., 2012), and this would fit both the phenotypic and molecular observations of PPV–apricot interaction. Cosson et al. (2012) suggested that mutations in RTM proteins can either alter their stability or increase their degradation.

Six genes encoding MATHd proteins were reported by Zuriaga et al. (2013; Zuriaga et al., 2018). We confirmed the occurrence of these genes, coded as *Pa31* to *Pa36*, in apricot, and the fine reconstruction of both R and S haplotypes in ‘Lito’ allowed the accurate comparison of all these genes at sequence level other than the analysis of their specific allelic expression levels.

Our findings suggest that among the six MATHd genes, *Pa31* and *Pa33*, overexpressed in the R haplotype, and *Pa34*, expressed mainly in the R haplotype and encoding for a truncated form of the protein in the S haplotype, would fit the hypothesis of the control of PPV by limiting the virus movement, as suggested in the literature (Cosson et al., 2012). Our data would disagree with the hypothesis of Zuriaga et al. (2018) hinting at *Pa31* and *Pa32* as possible host susceptibility (recessive) genes and their silencing as explanation for PPV resistance. These findings would conflict with the literature that considers the expression of this class of genes as a necessary condition of resistance (Whitham et al., 1999; Decroocq et al., 2006; Cosson et al., 2010). Moreover, *Pa31* was found preferentially expressed in the R haplotype, in contrast with the conclusions of Zuriaga et al. (2018). In that paper, a chimeric assembly of the two alleles R and S probably occurred, and such an artifact would have affected primer design, preventing the amplification of *Pa31* in ‘Stella’, the cultivar homozygous for resistance. Our approach, based on assembling the alleles separately, would suggest *Pa31* (*Par3* in Zuriaga et al., 2018) as a MATHd candidate gene in preventing the PPV diffusion in apricot.

The implication of squamosa promoter-binding-like protein 1 (*Pa22* and *Pa23*) in PPV resistance in apricot seems unlikely, as reported elsewhere (Zuriaga et al., 2013). Although *Pa23* displayed a higher expression in R, the encoded polypeptide appeared defective, whereas *Pa22* did not exhibit any difference either in the sequence or in the expression level in the two haplotypes.

The role of Pto-interacting protein 1-like (*Pa21*) has been recently called into question, mainly due to its role in the hypersensitive response (Zhou et al., 1995) which has not been observed in the interaction between PPV and apricot (Zuriaga et al., 2013). The *Oryza sativ*a Pto-interacting protein 1a (*OsPti1a*) has recently been demonstrated to operate as a negative regulator of innate immunity in rice. The activation of immune responses, which includes a hypersensitive response-like cell death, is caused by loss of the *OsPti1a* protein (Matsui et al., 2014). Our finding demonstrated that the expression of Pto-interacting protein 1-like is greater in the S haplotype, and a premature stop codon leads to a truncated form in the R haplotype. The action mechanisms of these proteins are still controversial, and the heterozygous loss-of-function mutation in ‘Lito’ genome can raise several doubts, but our findings would suggest cautious reconsideration of the possible role of Pto-interacting protein 1-like in PPV resistance.

Several other genes identified in our screening cannot be excluded from the list of possible candidates (e.g., *Pa44* and *Pa46*), but their role remain uncharacterized or, to date, poorly related to resistance mechanisms. Remarkably, sequence analysis also identified that the transcript *Pa38* is the apricot homolog of the *Arabidopsis* protein GFS12. This BEACH-domain protein is involved in the effector-triggered immunity (ETI) in plants. Teh et al. (2015) proposed that GFS12 acts predominantly to suppress a negative factor in plant immunity response. Our results showed that this gene is mainly expressed in the R haplotype and a deletion in the functional domain was detected in the S allele. These observations are consistent with the hypothesis of a recessive loss-of-function mutation in a gene possibly involved in PPV resistance mechanisms, with homozygous mutation leading to a susceptible phenotype.

In this paper, we provided an accurate assembly of the major QTL of the PPV locus, the annotation of the genes in that region, and the analysis of allelic variants. The major QTL investigated is probably necessary but not sufficient for explaining resistance (Lambert et al., 2007; Soriano et al., 2008; Marandel et al., 2009; Dondini et al., 2011; Rubio et al., 2014; Mariette et al., 2016), and a second locus, named *PPV1b* in the literature to distinguish it from the first one (*PPV1a*) and not yet identified, should be required for the resistance being deployed (Mariette et al., 2016). The occurrence of two independent genes with epistatic effect could explain the genotype versus phenotype discrepancies found in our three recombinants (i.e., GPI in **Figure 2**) by testing markers, used for selection in apricot breeding, tightly linked to the *PPV1a* locus (Soriano et al., 2012; Zuriaga et al., 2013; Rubio et al., 2014; Passaro et al., 2017). Furthermore, the ambiguity in the detection of susceptibility and resistance symptoms still represents a bottleneck, hampering the precise scoring of the different phenotypic classes (Rubio et al., 2007; Rubio et al., 2014; Mariette et al., 2016). Although our results do not provide a conclusive hypothesis, we addressed the important issue of narrowing the shortlist of possible candidate genes. Given the complexity of the molecular dialogue underlying plant–virus interplay, to date, we are not able to exclude genes, even with currently unknown function, other than MATHd, as resistance candidates. In addition, the complete and accurate sequence of contrasting haplotypes in the region might be used to predict and test the possible role of alternative transcription and regulation mechanisms (i.e., miRNAs) involved in the process. Undoubtedly, the transformation, which still represents a significant challenge in stone fruit trees, shall functionally validate the role of candidate gene/s in the PPV resistance.

## Data Availability Statement

Full sequence data of the R and S regions spanning the QTL area under investigation have been deposited, along with the BAC end sequences and NGS sequences, with link to Bioproject accession number PRJNA560760 in the NCBI (http://http://www.ncbi.nlm.nih.gov/bioproject). 

## Author Contributions

LD, ST, FG, and RT conceived and planned the whole research. DB conceived the crossing population. GDM, RF, SS, RT, FS, LD, and ST scouted the new markers. LD created the new linkage map on the extended population. FP and AM did the phenotypic screening of recombinants. GDM, RF, FS, FG, LD, and ST screened the BAC library. AS and GDM sequenced the BAC clones. GDM and SS assembled sequences and curated the gene prediction and annotation with the contribution of LD and ST. RT and GDM wrote the first draft of the text. All authors contributed to the final version of the manuscript.

## Funding

This work was partially supported by the Ministry of University and Research of Italy—Project of Relevant National Interest (PRIN) code 2012KKNMWC “Molecular strategies to gain resistance to Sharka viruses (PPV) in peach and apricot.” It was also partially supported by the “Marker Assisted Resistance to Sharka (MARS)” Small Collaborative FP7 project no. 613654, 2013–2015.

## Conflict of Interest

The authors declare that the research was conducted in the absence of any commercial or financial relationships that could be construed as a potential conflict of interest.
