# Supplementary material for: Resistance to Sharka in Apricot: Comparison of Phase-Reconstructed Resistant and Susceptible Haplotypes of ‘Lito’ Chromosome 1 and Analysis of Candidate Genes
Source: Front Plant Sci. 2019 Dec 4;10:1576. doi: 10.3389/fpls.2019.01576 (PMC6905379; doi:10.3389/fpls.2019.01576)
Supplement: Supplementary file 1 [file DataSheet_1.zip › Figure 1.DOCX]

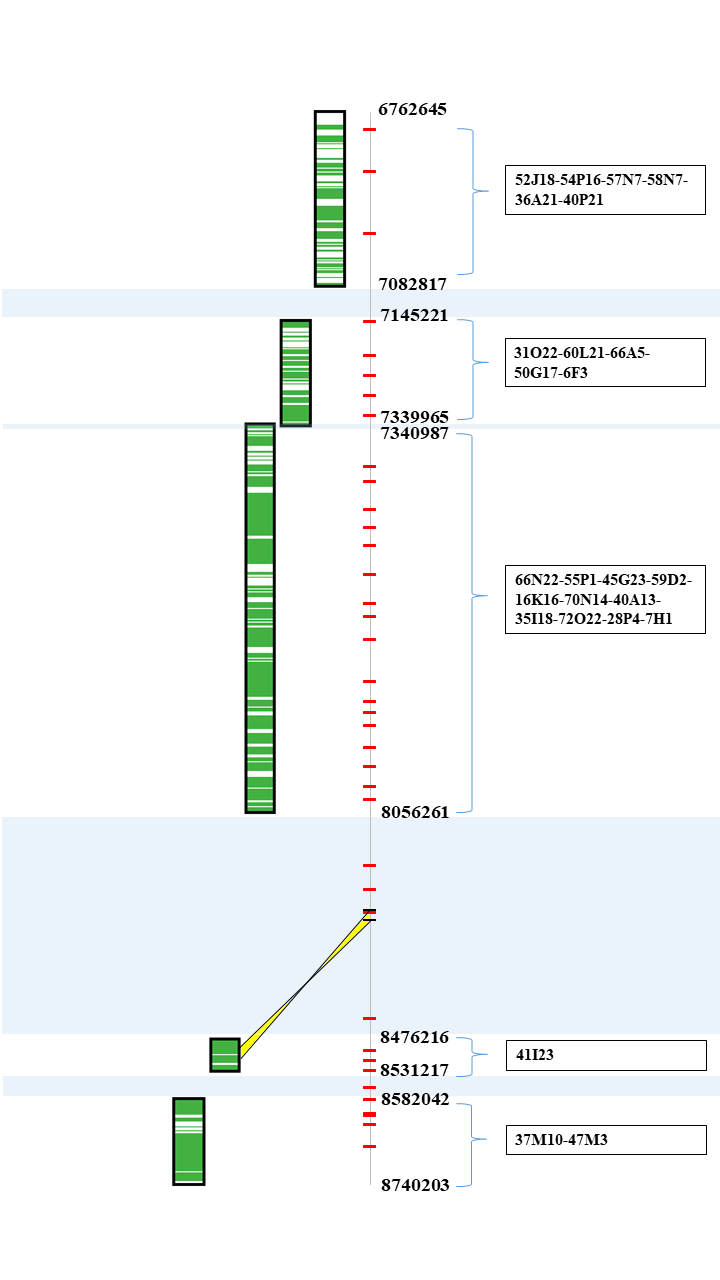


**Supplementary Figure 1A.** BAC supercontigs of ‘Lito’ resistant haplotype aligned to the peach sequence v 2.0. BAC supercontigs (right) grouped in scaffolds (left) form a minimum tiling path. The green regions within the scaffolds show the sequences shared by apricot and peach. Scaffolds on the left are aligned to peach sequence v 2.0 (center) in the interval of 6,762,645 – 8,740,203 bp. Markers of the ‘Lito’ linkage map are reported as short red lines with their relative position in the peach genome. Light blue background represents the peach sequence uncovered by apricot BACs. In yellow a sequence inversion found in apricot.


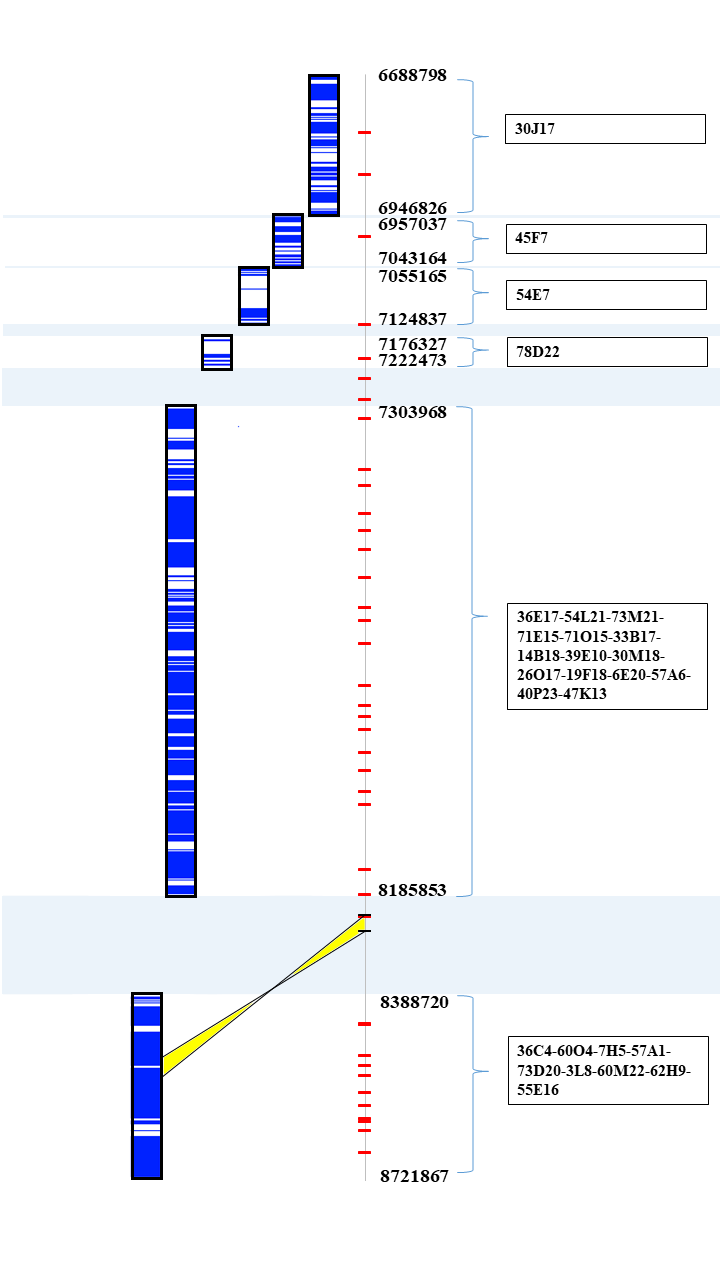


**Supplementary Figure 1B.** BAC supercontigs of ‘Lito’ susceptible haplotype aligned to the peach sequence v 2.0. BAC supercontigs (right) collected in scaffolds (left) form a minimum tiling path. The blu regions within the scaffolds show the sequences shared by apricot and peach. Scaffolds of the left are aligned to peach sequence v 2.0 (center) in the interval of 6,688,798 – 8,721,867 bp. Markers of the ‘Lito’ linkage map are reported as short red lines with their relative position in the peach genome. Light blue background represents the peach sequence uncovered by apricot BACs. In yellow a sequence inversion found in apricot.
