# Supplementary material for: Resistance to Sharka in Apricot: Comparison of Phase-Reconstructed Resistant and Susceptible Haplotypes of ‘Lito’ Chromosome 1 and Analysis of Candidate Genes
Source: Front Plant Sci. 2019 Dec 4;10:1576. doi: 10.3389/fpls.2019.01576 (PMC6905379; doi:10.3389/fpls.2019.01576)
Supplement: Supplementary file 1 [file DataSheet_1.zip › Figure 2.DOCX]

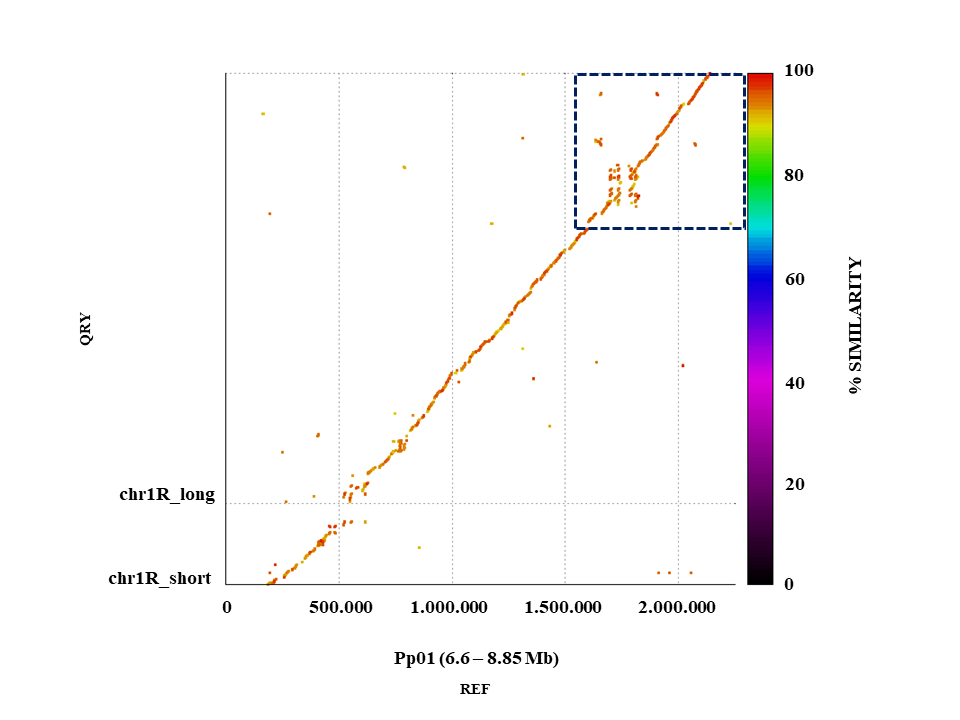


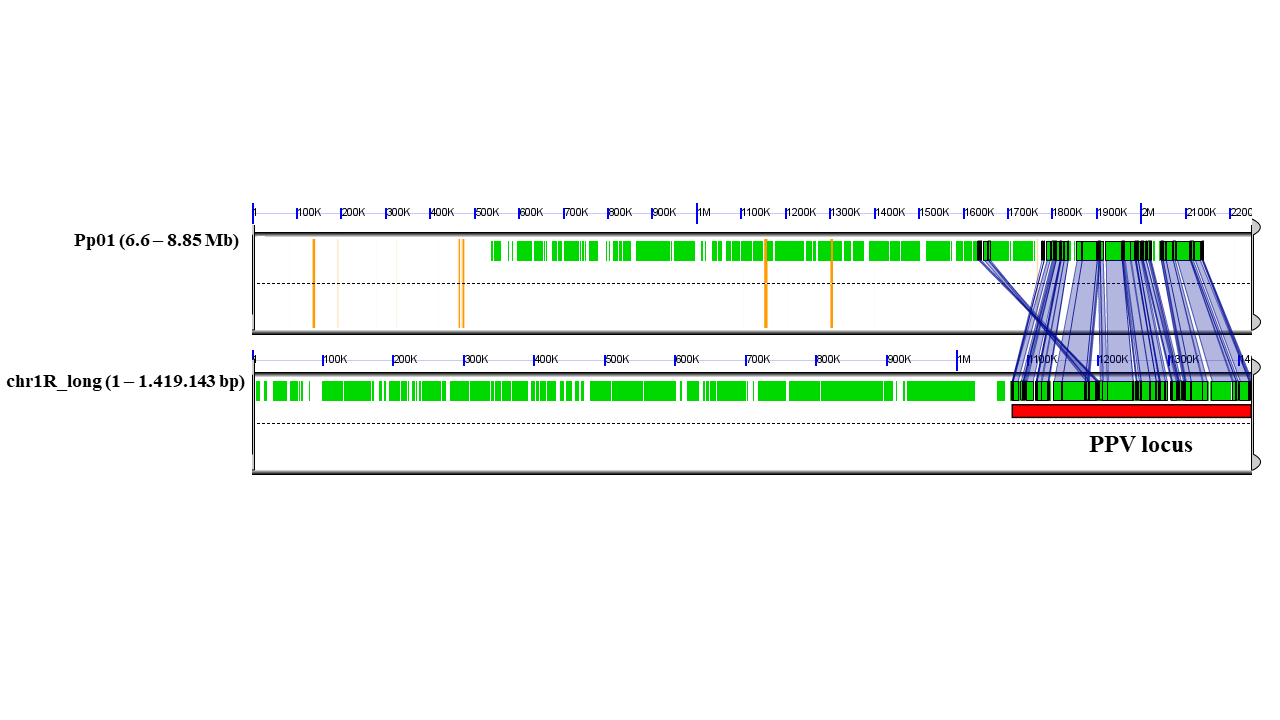


**Supplementary Figure 2a.** (Top) Alignment between the reference peach genome v 2.0 (REF, 6.60 – 8.85 Mbp) and ‘Lito’(QRY) resistant haplotype. Plot was created using NUCMer. The percentage of similarity is shown by the color bar. The dotted area highlight a region characterized by clear rearrangement between apricot and peach genome. (Bottom) Alignment between the reference peach genome (Pp01, 6.60 – 8.85 Mbp) and the long fragment of ‘Lito’ resistant haplotype (chr1R_long). Graph was created using Gevo. Blue connectors show the shared regions between peach and apricot highlighting the inversion found in apricot. Orange bars indicate sequence gaps in the peach genome. Red bar shows the analyzed PPV locus (236 kb).


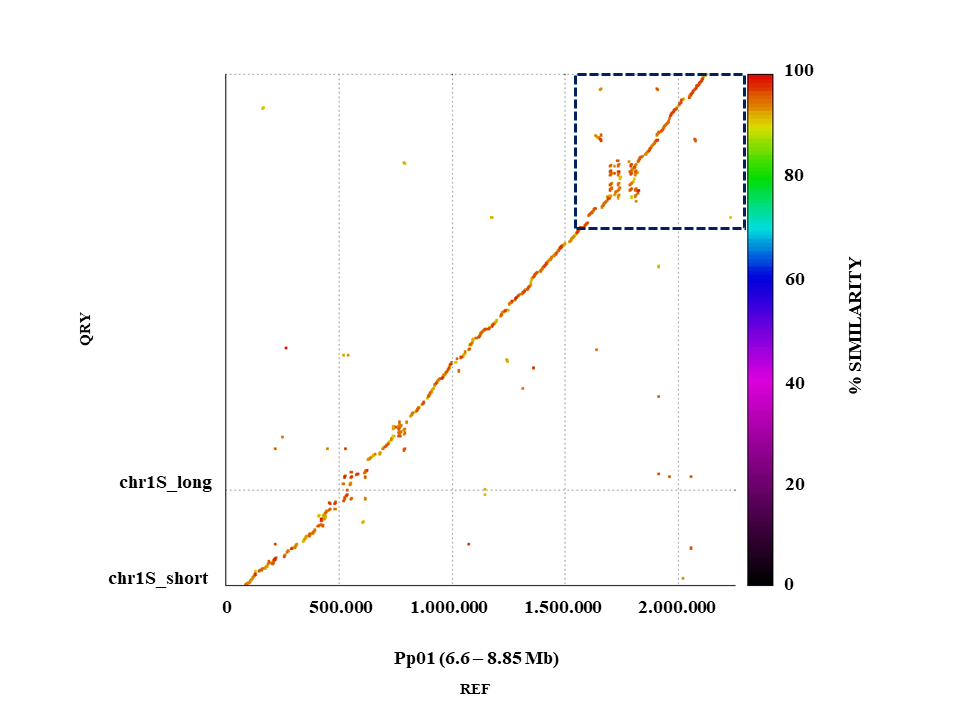


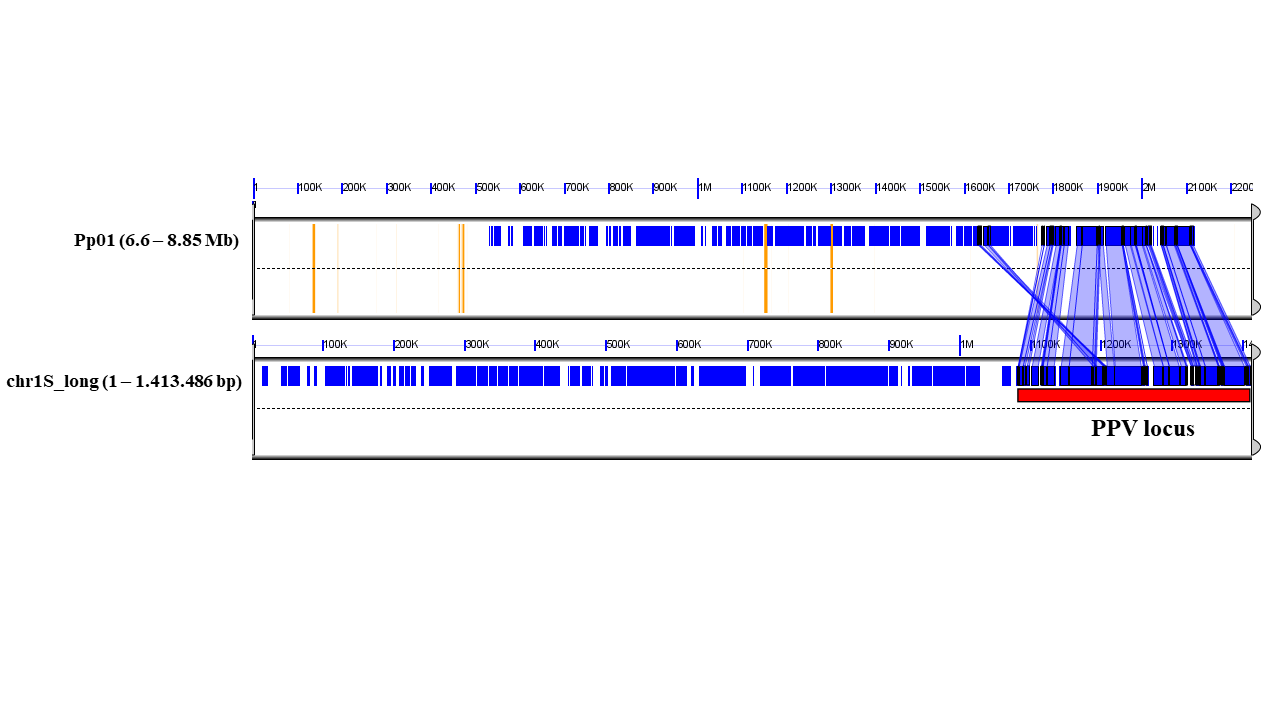


**Supplementary Figure 2B.** (Top) Alignment between the reference peach genome v 2.0 (REF, 6.60 – 8.85 Mbp) and ‘Lito’(QRY) susceptible haplotype. Plot was created using NUCMer. The percentage of similarity is shown by the color bar. The dotted area highlight a region characterized by clear rearrangement between apricot and peach genome.

(Bottom) Alignment between the reference peach genome (Pp01, 6.60 – 8.85 Mbp) and the long fragment of ‘Lito’ susceptible haplotype (chr1S_long). Graph was created using Gevo. Blue connectors show the shared regions between peach and apricot highlighting the inversion found in apricot. Orange bars indicate sequence gaps in the peach genome. Red bar shows the analyzed PPV locus (236 kb).

**
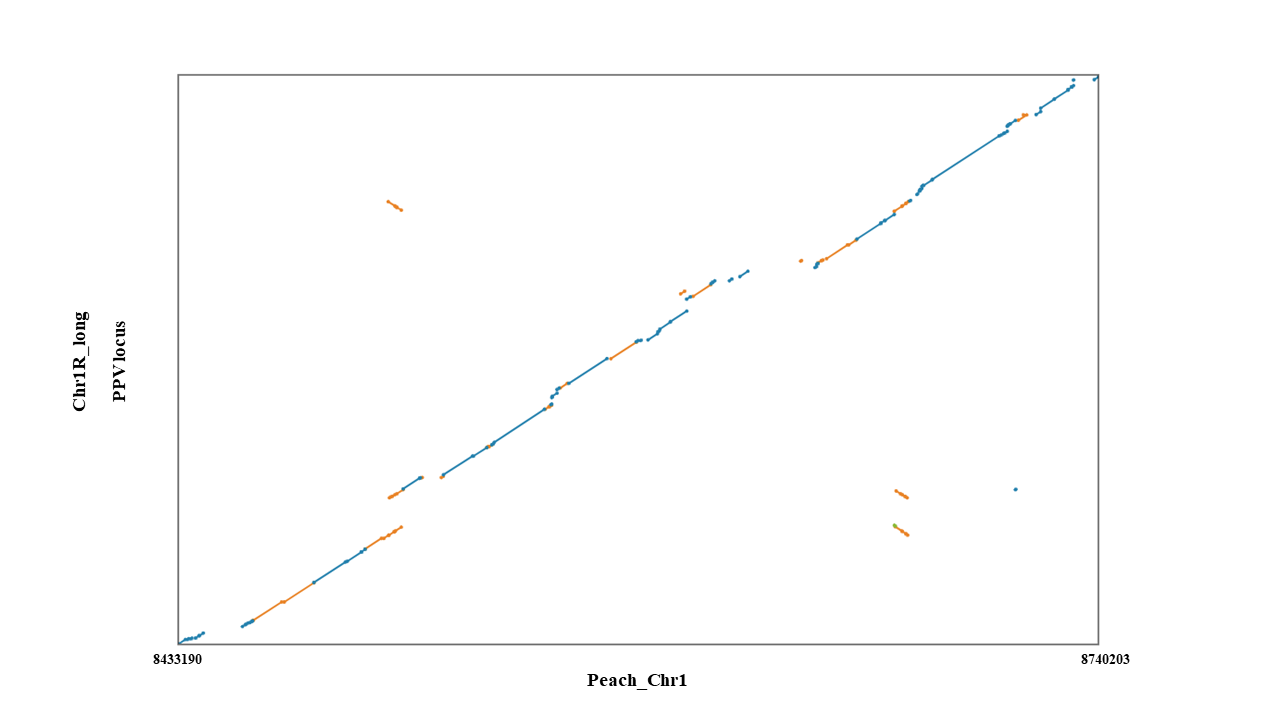
**

**Supplementary Figure 2C.** Dot plot alignment between peach genome v2.0 (8,433,190 – 8,740,203) and PPV locus of ‘Lito’ resistant haplotype. Dot plot was created using NUCMer and the interactive dot plot viewer for genome-genome alignments Dot (https://dnanexus.github.io/dot). Blue lines represent the unique forward alignments, green lines the unique reverse alignments and orange lines the repetitive alignments.

**
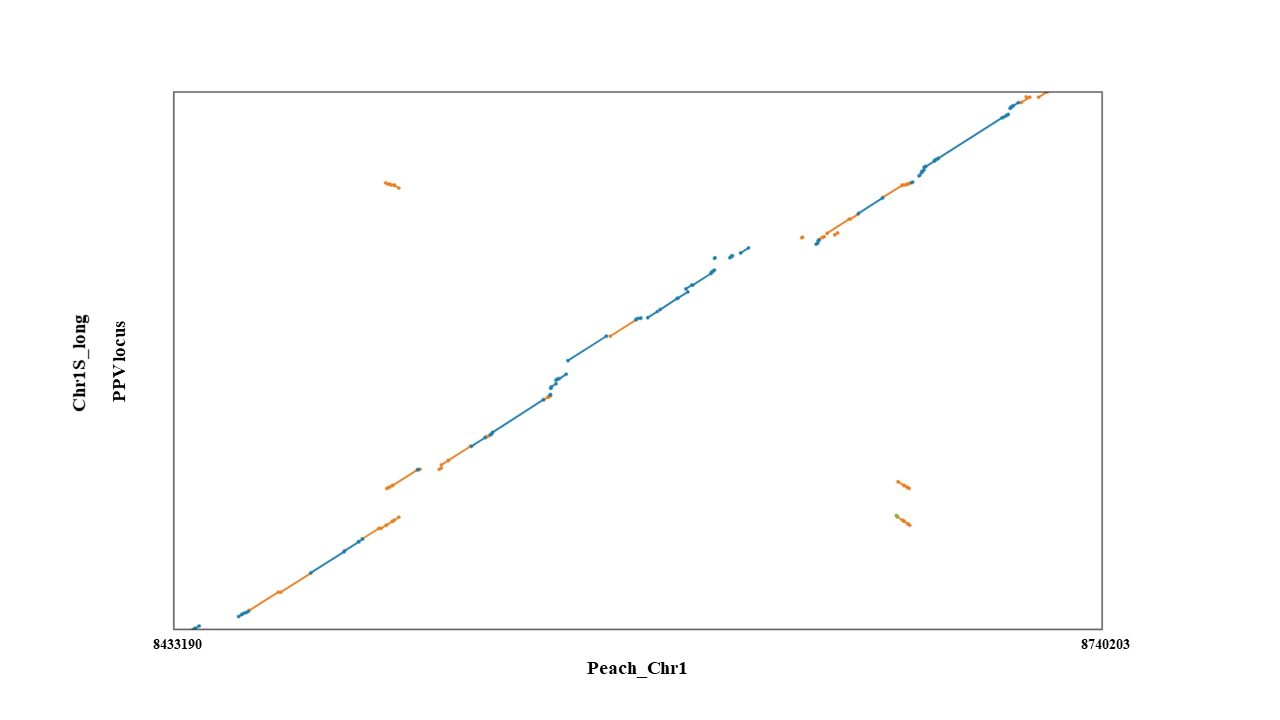
**

**Supplementary Figure 2D.** Dot plot alignment between peach genome v2.0 (8,433,190 – 8,740,203) and PPV locus of ‘Lito’ resistant haplotype. Dot plot was created using NUCMer and the interactive dot plot viewer for genome-genome alignments Dot (https://dnanexus.github.io/dot). Blue lines represent the unique forward alignments, green lines the unique reverse alignments and orange lines the repetitive alignments.
