# Supplementary material for: Resistance to Sharka in Apricot: Comparison of Phase-Reconstructed Resistant and Susceptible Haplotypes of ‘Lito’ Chromosome 1 and Analysis of Candidate Genes
Source: Front Plant Sci. 2019 Dec 4;10:1576. doi: 10.3389/fpls.2019.01576 (PMC6905379; doi:10.3389/fpls.2019.01576)
Supplement: Supplementary file 1 [file DataSheet_1.zip › Figure 3.DOCX]

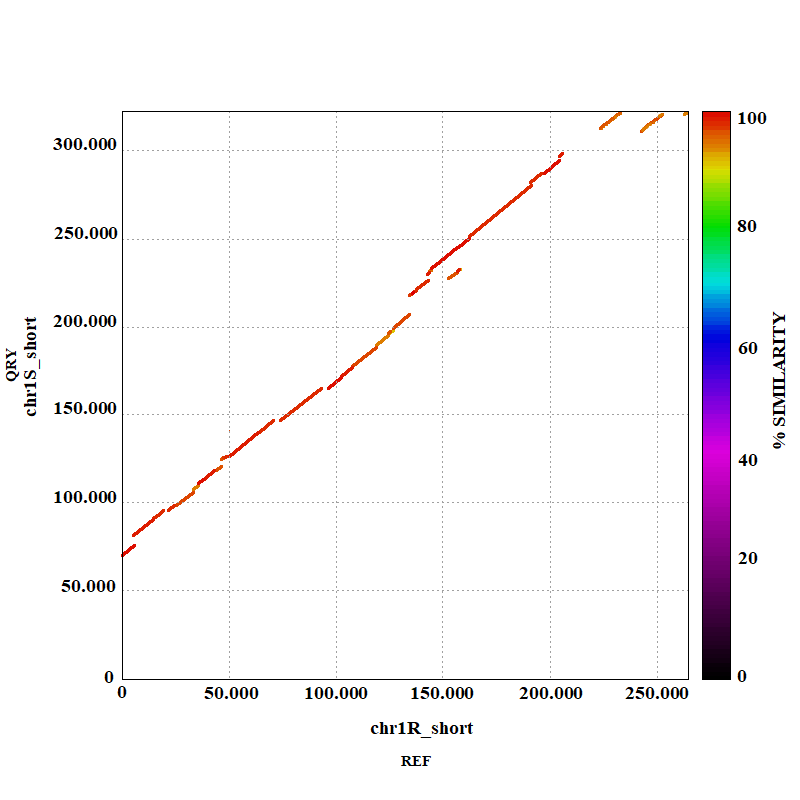

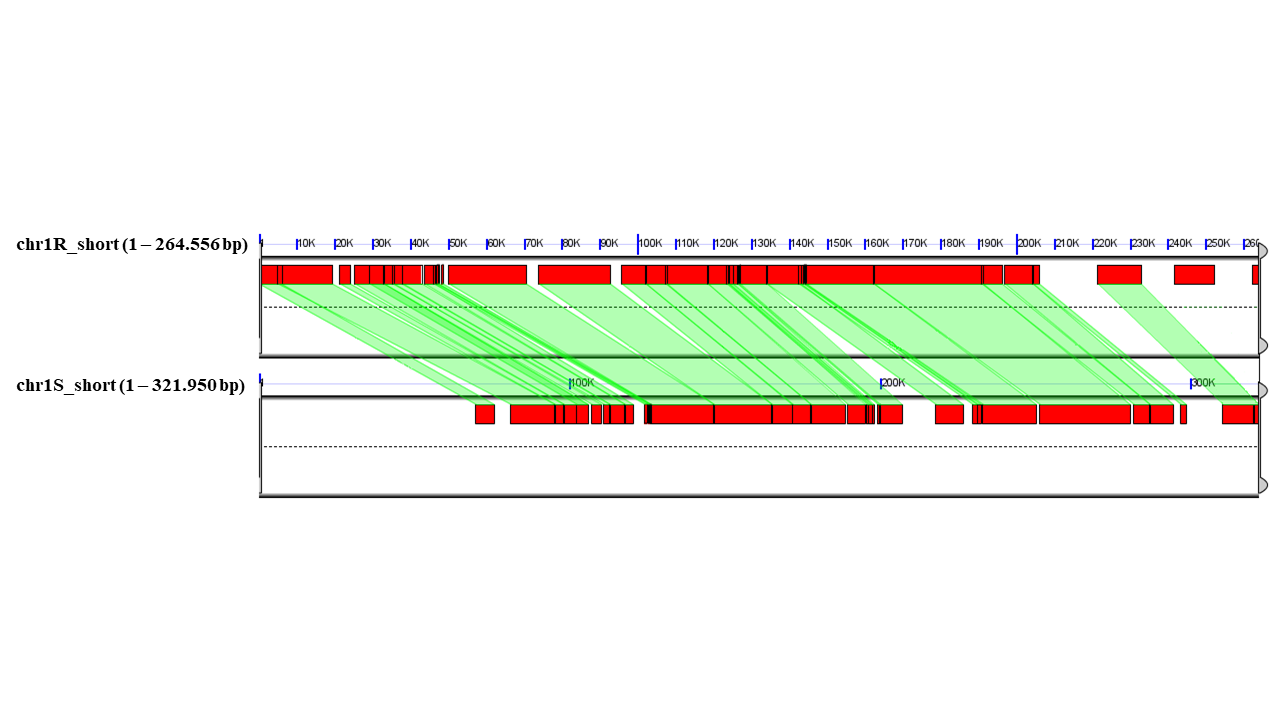

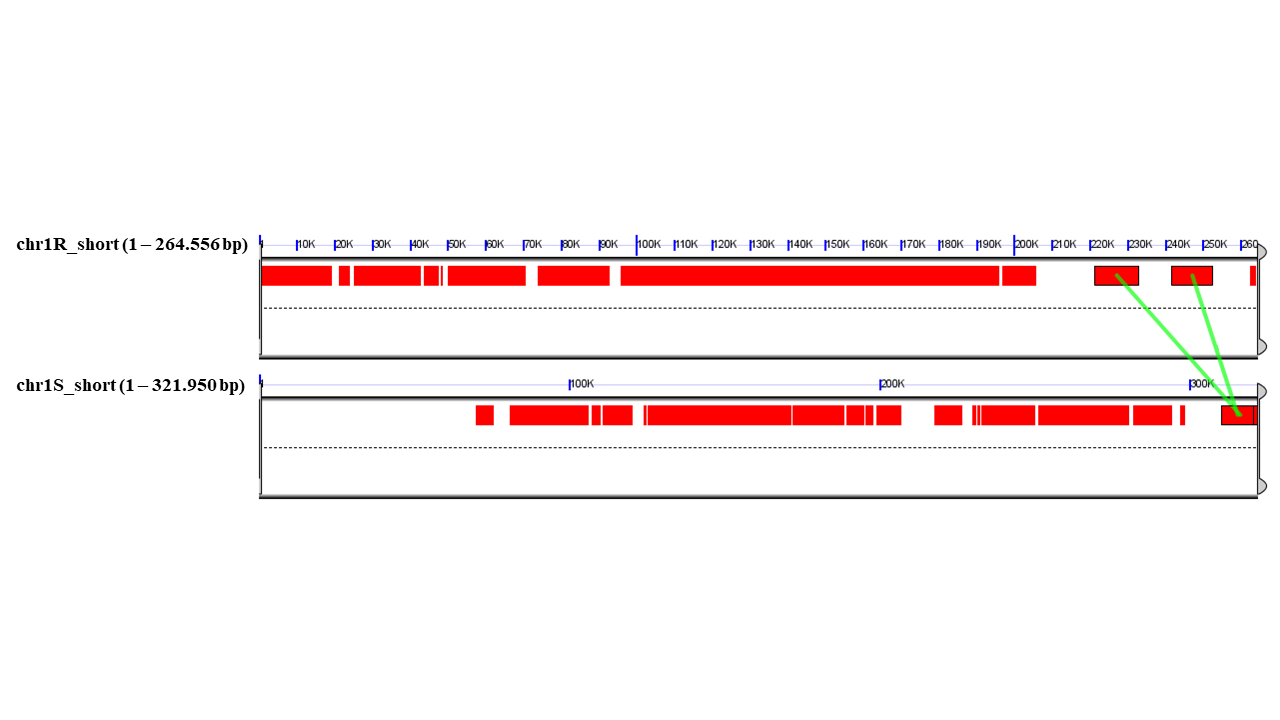


**Supplementary Figure 3a.** In the upper panel is shown the alignment of apricot chr1R_short against chr1S_short. Plot was created using NUCMer. Alignment of apricot chr1R_short against chr1S_short is represented in the middle panel. Graph was created using Gevo. Green connectors show the shared regions between the two haplotypes. White spaces highlight insertions within the sequences of the resistant haplotype compared to the susceptible ones and vice versa. On the bottom the alignment of apricot chr1R_short against chr1S_short is shown. Graph was created using Gevo. Green connectors show the duplication in the chr1R_short: the assembly of this region was difficult for both haplotypes because of several duplications. For this reason, the assembly was obtained only for one of the duplicates in susceptible haplotype.

**
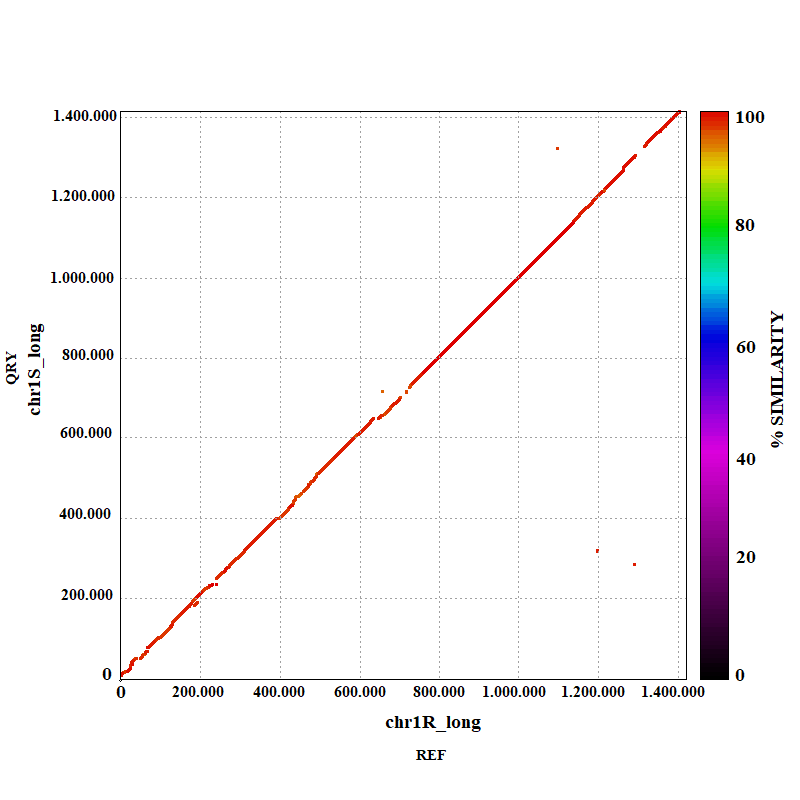
**


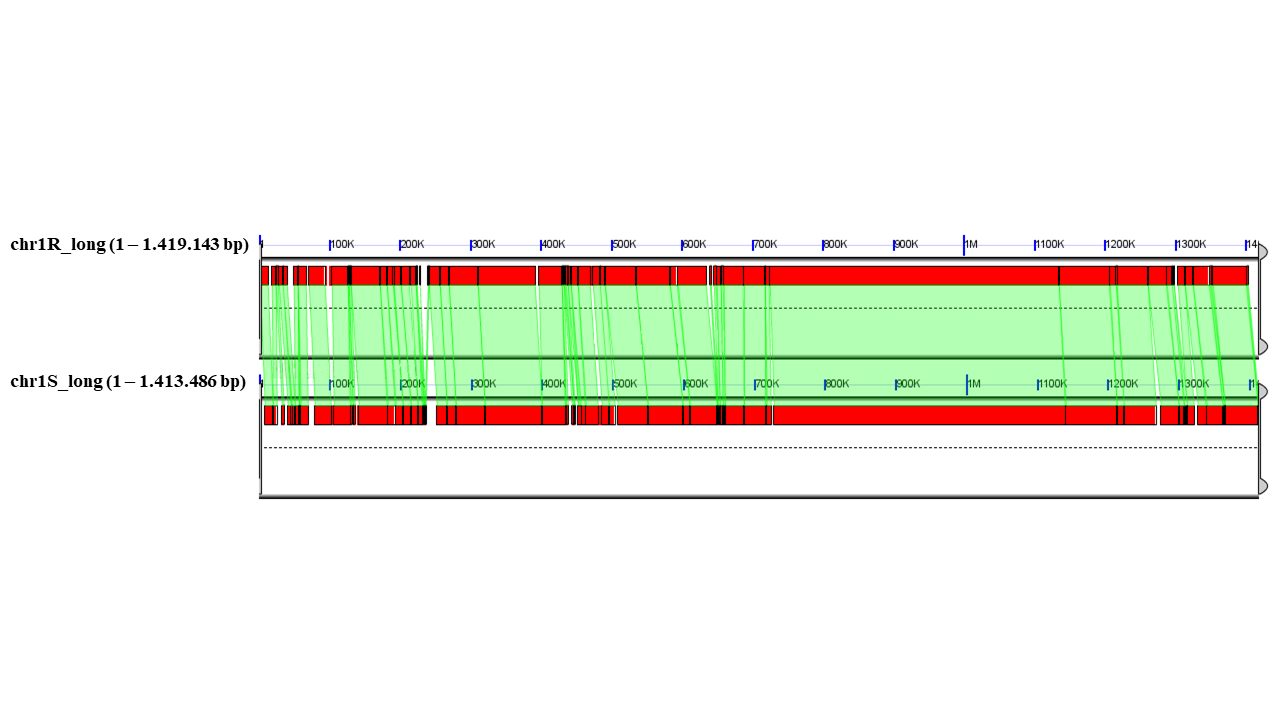


**Supplementary Figure 3B.** (Top) Alignment of apricot chr1R_long against chr1S_long. Plot was created using NUCMer. (Bottom) Alignment of apricot chr1R_long against chr1S_long. Graph was created using Gevo. Green connectors show the shared regions between the two haplotypes. White spaces highlight insertions within the sequences of the resistant haplotype compared to the susceptible ones and vice versa.
