# Supplementary material for: Resistance to Sharka in Apricot: Comparison of Phase-Reconstructed Resistant and Susceptible Haplotypes of ‘Lito’ Chromosome 1 and Analysis of Candidate Genes
Source: Front Plant Sci. 2019 Dec 4;10:1576. doi: 10.3389/fpls.2019.01576 (PMC6905379; doi:10.3389/fpls.2019.01576)
Supplement: Supplementary file 1 [file DataSheet_1.zip › Figure 4.DOCX]

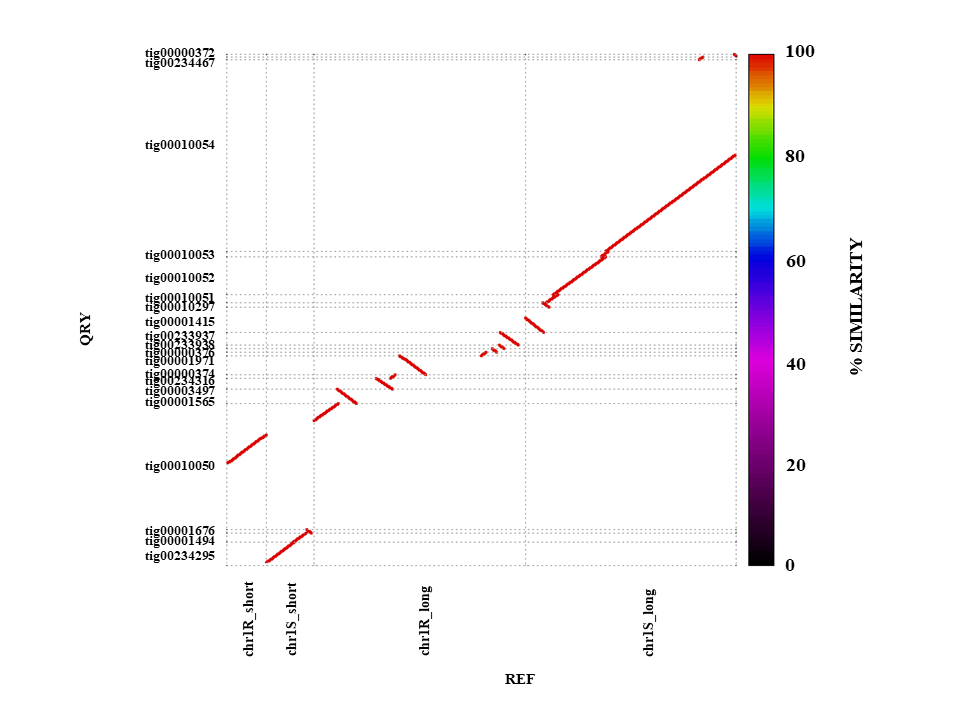


**Supplementary Figure 4a** - Nucmer plot obtained aligning contigs from the Whole genome assembly of ‘Lito’ against the assembled sequences for the two haplotypes of the region (chrR_short, chrS_short, chrR_long, chrS_long). Contigs have similarity closest to 100% with the assembled sequences. Canu assembler was able to reconstruct different contigs wherever the allelic divergence was greater than the post-correction overlap error rate.


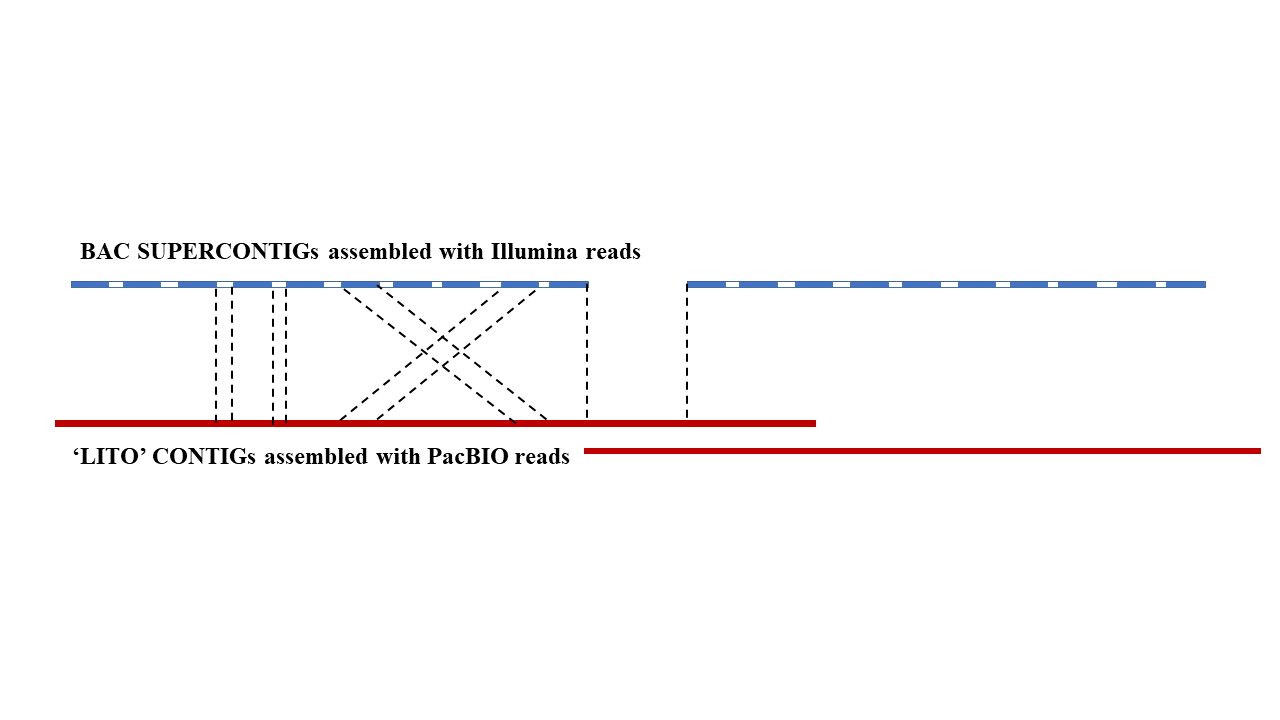


**Supplementary Figure 4B**  – Example of how PacBIO read assembly might help ordering and merging BAC supercontigs and filling the gaps.
