# Supplementary material for: Resistance to Sharka in Apricot: Comparison of Phase-Reconstructed Resistant and Susceptible Haplotypes of ‘Lito’ Chromosome 1 and Analysis of Candidate Genes
Source: Front Plant Sci. 2019 Dec 4;10:1576. doi: 10.3389/fpls.2019.01576 (PMC6905379; doi:10.3389/fpls.2019.01576)
Supplement: Supplementary file 1 [file DataSheet_1.zip › Figure 6.DOCX]

**Supplementary Figure 6**. Comparison of protein and CDS sequences displaying structural variation in R and S haplotypes, in the PPV locus.

**Pa9 - Putative transcription factor/ chromatin remodeling BED-type(Zn) family**

Aligned_sequences: 2

1: **Par.chr1R_long.5.96_B**

2: **Par.chr1S_long.5.87_B**

Matrix: EBLOSUM62

Gap_penalty: 10.0

Extend_penalty: 0.5

Length: 906

Identity: 669/906 (73.8%)

Similarity: 670/906 (74.0%)

Gaps: 236/906 (26.0%)

Score: 3521.0

R 1 MRSSGLVDPGWEHGMAQDERKKKVKCNYCGKIVSGGIYRLKQHLARVSGE 50

||||||||||||||||||||||||||||||||||||||||||||||||||

S 1 MRSSGLVDPGWEHGMAQDERKKKVKCNYCGKIVSGGIYRLKQHLARVSGE 50

R 51 VTYCDKAPEDVYMSMKANMEGSRSNKKPRHSEDIGQAYLNFQSNDDEEEV 100

||||||||||||||||||||||||||||||||||||||||||||||||||

S 51 VTYCDKAPEDVYMSMKANMEGSRSNKKPRHSEDIGQAYLNFQSNDDEEEV 100

R 101 HVGYRSKGKQLMGDRNLAMKLTPLRSLGYVDPGWEHGVAQDEKKKKVKCI 150

||||||||||||||||||||||||||||||||||||||||||||||||||

S 101 HVGYRSKGKQLMGDRNLAMKLTPLRSLGYVDPGWEHGVAQDEKKKKVKCI 150

R 151 YCEKIVSGGINRFKQHLARIPGEVAPCKHAPEEVFLKIKENMKWHRTGRR 200

||||||||||||||||||||||||||||||||||||||||||||||||||

S 151 YCEKIVSGGINRFKQHLARIPGEVAPCKHAPEEVFLKIKENMKWHRTGRR 200

R 201 QRQPDSKDMSPFDLQSDNEDQDDDQMEAALHHINKERLIDGDRRLGQNLR 250

||||||||||||||||||||||||||||||||||||||||||||||||||

S 201 QRQPDSKDMSPFDLQSDNEDQDDDQMEAALHHINKERLIDGDRRLGQNLR 250

R 251 NTFKALPPSTGSEPLFKRSRLDSLFLTAPKSLTPHSYRQVRVRTMSNKIS 300

||||||||||||||||||||||||||||||||||||||||||||||||||

S 251 NTFKALPPSTGSEPLFKRSRLDSLFLTAPKSLTPHSYRQVRVRTMSNKIS 300

R 301 RKEVISGICKFFYHAGVPLQATNSLYFHKMLELVGQYGQGLVAPPSQLIS 350

||||||||||||||||||||||||||||||||||||||||||||||||||

S 301 RKEVISGICKFFYHAGVPLQATNSLYFHKMLELVGQYGQGLVAPPSQLIS 350

R 351 GRFLQEEIATIKTYLADYKASWAITGCSIMADSWRDTEGRILINFLASGP 400

||||||||||||||||||||||||||||||||||||||||||||||||||

S 351 GRFLQEEIATIKTYLADYKASWAITGCSIMADSWRDTEGRILINFLASGP 400

R 401 NGVYFVSSVDATEIVEDASNLFKLLDKVVEEMGEENVVQVITPITPSYKA 450

||||||||||||||||||||||||||||||||||||||||||||||||||

S 401 NGVYFVSSVDATEIVEDASNLFKLLDKVVEEMGEENVVQVITPITPSYKA 450

R 451 AGNMLEEKRKKLFWTPCATSCIDQMLEDFLKIRSVAECMEKGQKITKLIY 500

||||||||||||||||||||||||||||||||||||||||||||||||||

S 451 AGNMLEEKRKKLFWTPCATSCIDQMLEDFLKIRSVAECMEKGQKITKLIY 500

R 501 NQIWLLNFLKSDFTQGKELLRPSITRFASSFATLQSLLDHRTGLRRMFQS 550

||||||||||||||||||||||||||||||||||||||||||||||||||

S 501 NQIWLLNFLKSDFTQGKELLRPSITRFASSFATLQSLLDHRTGLRRMFQS 550

R 551 NKWISSQCSKSCEGKEVESIVLNATFWKKLQFVRNSVDPIMQVLQKVETG 600

||||||||||||||||||||||||||||||||||||||||||||||||||

S 551 NKWISSQCSKSCEGKEVESIVLNATFWKKLQFVRNSVDPIMQVLQKVETG 600

R 601 DCLSMSSIYNDMYRAKLAIKTIHGDNVRKYEPFWSVIESHWNSLFYHPVY 650

||||||||||||||||||||||||||||||||||||||||||||||||||

S 601 DCLSMSSIYNDMYRAKLAIKTIHGDNVRKYEPFWSVIESHWNSLFYHPVY 650

R 651 VAAYYLNPSYRYRPDFTAYRGDAWT------------------------- 675

||||||||||||||||||: |

S 651 VAAYYLNPSYRYRPDFTAH-----TEGMRGLNECIVRLEPDSARRISASM 695

R 676 -------------------------------------------------- 675

S 696 QISDYNSAKADFGTELAISTRTELDPAAWWQQHGISCLELQRIAVRILSQ 745

R 676 -------------------------------------------------- 675

S 746 TCSSFGCEHNWSIYDQLYSLRNNRLAQKRLNDLIYVHYNLRLREQQLRRR 795

R 676 -------------------------------------------------- 675

S 796 ADNSISLDNILLERLLDDWIVDAAENDMLENEEVLYNEIEQVDEYENDMV 845

R 676 -------------------------------------------------- 675

S 846 DYEGVNGNAETRNGSVELLTLADADINPANAGVATDDDDEDDEDGDINFF 895

R 676 ------ 675

S 896 DDDMSD 901

Aligned_sequences: 2

1: **Par.chr1R_long.5.96_B**

2: **Par.chr1S_long.5.87_B**

Matrix: EDNAFULL

Gap_penalty: 10.0

Extend_penalty: 0.5

Length: 2706

Identity: 2696/2706 (99.6%)

Similarity: 2696/2706 (99.6%)

Gaps: 4/2706 ( 0.1%)

Score: 13435.0

Green color: START CODON

Red color: STOP CODON

R 1 ATGCGCTCCTCTGGACTTGTTGACCCTGGATGGGAACATGGCATGGCTCA 50

||||||||||||||||||||||||||||||||||||||||||||||||||

S 1 ATGCGCTCCTCTGGACTTGTTGACCCTGGATGGGAACATGGCATGGCTCA 50

R 51 AGATGAGAGGAAGAAAAAGGTTAAATGCAATTACTGTGGAAAAATAGTTA 100

||||||||||||||||||||||||||||||||||||||||||||||||||

S 51 AGATGAGAGGAAGAAAAAGGTTAAATGCAATTACTGTGGAAAAATAGTTA 100

R 101 GTGGAGGAATATATAGATTGAAGCAACATTTAGCCCGAGTTTCTGGAGAA 150

||||||||||||||||||||||||||||||||||||||||||||||||||

S 101 GTGGAGGAATATATAGATTGAAGCAACATTTAGCCCGAGTTTCTGGAGAA 150

R 151 GTTACTTATTGTGATAAGGCTCCAGAGGATGTATACATGAGTATGAAAGC 200

||||||||||||||||||||||||||||||||||||||||||||||||||

S 151 GTTACTTATTGTGATAAGGCTCCAGAGGATGTATACATGAGTATGAAAGC 200

R 201 AAATATGGAAGGAAGTCGTTCTAATAAGAAACCTAGGCATTCTGAAGATA 250

||||||||||||||||||||||||||||||||||||||||||||||||||

S 201 AAATATGGAAGGAAGTCGTTCTAATAAGAAACCTAGGCATTCTGAAGATA 250

R 251 TTGGGCAAGCATATTTGAATTTCCAATCTAATGATGATGAAGAAGAGGTG 300

||||||||||||||||||||||||||||||||||||||||||||||||||

S 251 TTGGGCAAGCATATTTGAATTTCCAATCTAATGATGATGAAGAAGAGGTG 300

R 301 CATGTTGGTTATAGAAGCAAAGGAAAGCAATTGATGGGTGACAGGAATTT 350

||||||||||||||||||||||||||||||||||||||||||||||||||

S 301 CATGTTGGTTATAGAAGCAAAGGAAAGCAATTGATGGGTGACAGGAATTT 350

R 351 AGCTATGAAGTTGACTCCTCTTCGGTCATTAGGATATGTTGACCCTGGGT 400

|||||||||||||||||||||||||||||||||||||||.||||||||||

S 351 AGCTATGAAGTTGACTCCTCTTCGGTCATTAGGATATGTGGACCCTGGGT 400

R 401 GGGAACATGGTGTTGCTCAGGATGAGAAAAAGAAAAAGGTGAAATGCATT 450

||||||||||||||||||||||||||||||||||||||||||||||||||

S 401 GGGAACATGGTGTTGCTCAGGATGAGAAAAAGAAAAAGGTGAAATGCATT 450

R 451 TATTGCGAGAAAATAGTTAGTGGTGGCATCAATCGGTTTAAACAACATTT 500

||||||||||||||||||||||||||||||||||||||||||||||||||

S 451 TATTGCGAGAAAATAGTTAGTGGTGGCATCAATCGGTTTAAACAACATTT 500

R 501 AGCTAGAATTCCTGGAGAAGTAGCACCTTGTAAACATGCTCCTGAGGAAG 550

||||||||||||||||||||||||||||||||||||||||||||||||||

S 501 AGCTAGAATTCCTGGAGAAGTAGCACCTTGTAAACATGCTCCTGAGGAAG 550

R 551 TTTTTCTTAAAATAAAAGAGAATATGAAATGGCATCGTACTGGAAGGAGA 600

||||||||||||||||||||||||||||||||||||||||||||||||||

S 551 TTTTTCTTAAAATAAAAGAGAATATGAAATGGCATCGTACTGGAAGGAGA 600

R 601 CAGAGACAACCCGATTCAAAGGACATGTCACCTTTTGATCTGCAGTCAGA 650

||||||||||||||||||||||||||||||||||||||||||||||||||

S 601 CAGAGACAACCCGATTCAAAGGACATGTCACCTTTTGATCTGCAGTCAGA 650

R 651 CAATGAAGATCAAGACGATGACCAAATGGAAGCTGCTCTGCATCATATAA 700

||||||||||||||||||||||||||||||||||||||||||||||||||

S 651 CAATGAAGATCAAGACGATGACCAAATGGAAGCTGCTCTGCATCATATAA 700

R 701 ACAAGGAAAGATTGATTGATGGTGATAGGAGATTGGGCCAAAATTTGAGA 750

||||||||||||||||||||||||||||||||||||||||||||||||||

S 701 ACAAGGAAAGATTGATTGATGGTGATAGGAGATTGGGCCAAAATTTGAGA 750

R 751 AATACATTCAAGGCATTGCCTCCCAGTACTGGTTCTGAACCATTATTTAA 800

||||||||||||||.|||||||||||||||||||||||||||||||||||

S 751 AATACATTCAAGGCGTTGCCTCCCAGTACTGGTTCTGAACCATTATTTAA 800

R 801 AAGATCAAGGCTAGATTCCCTTTTCTTGACTGCTCCCAAGAGTTTGACAC 850

||||||||||||||||||||||||||||||||||||||||||||||||||

S 801 AAGATCAAGGCTAGATTCCCTTTTCTTGACTGCTCCCAAGAGTTTGACAC 850

R 851 CACATTCTTACAGACAAGTAAGGGTCAGAACAATGTCAAATAAAATATCC 900

||||||||||||||||||||||||||||||||||||||||||||||||||

S 851 CACATTCTTACAGACAAGTAAGGGTCAGAACAATGTCAAATAAAATATCC 900

R 901 CGCAAGGAAGTTATTTCTGGAATTTGCAAATTCTTTTACCATGCAGGAGT 950

||||||||||||||||||||||||||||||||||||||||||||||||||

S 901 CGCAAGGAAGTTATTTCTGGAATTTGCAAATTCTTTTACCATGCAGGAGT 950

R 951 TCCTCTACAAGCAACAAACTCCTTATACTTCCATAAGATGCTGGAATTGG 1000

||||||||||||||||||||||||||||||||||||||||||||||||||

S 951 TCCTCTACAAGCAACAAACTCCTTATACTTCCATAAGATGCTGGAATTGG 1000

R 1001 TTGGCCAATATGGCCAGGGTCTGGTAGCACCTCCAAGCCAATTAATATCT 1050

||||||||||||||||||||||||||||||||||||||||||||||||||

S 1001 TTGGCCAATATGGCCAGGGTCTGGTAGCACCTCCAAGCCAATTAATATCT 1050

R 1051 GGTCGGTTTCTGCAAGAGGAAATTGCAACCATTAAAACCTACCTCGCTGA 1100

||||||||||||||||||||||||||||||||||||||||||||.|||||

S 1051 GGTCGGTTTCTGCAAGAGGAAATTGCAACCATTAAAACCTACCTGGCTGA 1100

R 1101 TTATAAGGCGTCTTGGGCAATCACCGGGTGTTCTATAATGGCAGACAGTT 1150

||||||||||||||||||||||||.|||||||||||||||||||||||||

S 1101 TTATAAGGCGTCTTGGGCAATCACTGGGTGTTCTATAATGGCAGACAGTT 1150

R 1151 GGAGAGACACAGAGGGTAGGATATTAATAAATTTCTTGGCTTCTGGCCCA 1200

||||||||||||||||||||||||||||||||||||||||||||||||||

S 1151 GGAGAGACACAGAGGGTAGGATATTAATAAATTTCTTGGCTTCTGGCCCA 1200

R 1201 AATGGTGTATACTTTGTTTCTTCAGTTGATGCCACTGAAATAGTTGAAGA 1250

||||||||||||||||||||||||||||||||||||||||||||||||||

S 1201 AATGGTGTATACTTTGTTTCTTCAGTTGATGCCACTGAAATAGTTGAAGA 1250

R 1251 TGCTTCGAATTTGTTTAAGCTGCTGGACAAAGTGGTTGAAGAGATGGGTG 1300

||||||||||||||||||||||||||||||||||||||||||||||||||

S 1251 TGCTTCGAATTTGTTTAAGCTGCTGGACAAAGTGGTTGAAGAGATGGGTG 1300

R 1301 AGGAAAATGTAGTTCAGGTAATCACTCCAATTACTCCTAGCTATAAAGCT 1350

||||||||||||||||||||||||||||||||||||||||||||||||||

S 1301 AGGAAAATGTAGTTCAGGTAATCACTCCAATTACTCCTAGCTATAAAGCT 1350

R 1351 GCTGGAAATATGCTTGAAGAGAAAAGAAAGAAATTATTCTGGACCCCATG 1400

||||||||||||||||||||||||||||||||||||||||||||||||||

S 1351 GCTGGAAATATGCTTGAAGAGAAAAGAAAGAAATTATTCTGGACCCCATG 1400

R 1401 TGCCACCAGTTGTATTGATCAAATGCTTGAAGATTTTTTGAAGATAAGAA 1450

||||||||||||||||||||||||||||||||||||||||||||||||||

S 1401 TGCCACCAGTTGTATTGATCAAATGCTTGAAGATTTTTTGAAGATAAGAA 1450

R 1451 GTGTAGCGGAGTGCATGGAGAAGGGGCAAAAAATTACGAAGCTCATTTAC 1500

||||||||||||||||||||||||||||||||||||||||||||||||||

S 1451 GTGTAGCGGAGTGCATGGAGAAGGGGCAAAAAATTACGAAGCTCATTTAC 1500

R 1501 AACCAAATTTGGTTGTTAAATTTTCTGAAGAGTGATTTTACACAGGGGAA 1550

||||||||||||||||||||||||||||||||||||||||||||||||||

S 1501 AACCAAATTTGGTTGTTAAATTTTCTGAAGAGTGATTTTACACAGGGGAA 1550

R 1551 GGAACTTTTGAGACCGTCTATTACCCGATTCGCCTCTAGCTTTGCCACCT 1600

|||||||||||||||||||||||||||.||||||||||||||||||||||

S 1551 GGAACTTTTGAGACCGTCTATTACCCGGTTCGCCTCTAGCTTTGCCACCT 1600

R 1601 TACAAAGTTTGCTGGACCACAGGACTGGTCTTAGAAGAATGTTTCAATCA 1650

||||||||||||||||||||||||||||||||||||||||||||||||||

S 1601 TACAAAGTTTGCTGGACCACAGGACTGGTCTTAGAAGAATGTTTCAATCA 1650

R 1651 AACAAATGGATTTCATCTCAGTGCTCCAAATCATGTGAAGGAAAAGAGGT 1700

||||||||||||||||||||||||||||||||||||||||||||||||||

S 1651 AACAAATGGATTTCATCTCAGTGCTCCAAATCATGTGAAGGAAAAGAGGT 1700

R 1701 GGAAAGTATTGTATTAAATGCTACATTCTGGAAGAAGCTACAGTTTGTTA 1750

||||||||||||||||||||||||||||||||||||||||||||||||||

S 1701 GGAAAGTATTGTATTAAATGCTACATTCTGGAAGAAGCTACAGTTTGTTA 1750

R 1751 GGAATTCAGTGGACCCAATTATGCAAGTTCTTCAGAAGGTTGAGACTGGT 1800

||||||||||||||||||||||||||||||||||||||||||||||||||

S 1751 GGAATTCAGTGGACCCAATTATGCAAGTTCTTCAGAAGGTTGAGACTGGT 1800

R 1801 GACTGCTTGTCAATGTCATCTATATATAATGACATGTACAGGGCAAAGCT 1850

||||||||||||||||||||||||||||||||||||||||||||||||||

S 1801 GACTGCTTGTCAATGTCATCTATATATAATGACATGTACAGGGCAAAGCT 1850

R 1851 TGCAATCAAAACCATTCATGGTGACAATGTACGTAAATATGAACCATTTT 1900

||||||||||||||||||||||||||||||||||||||||||||||||||

S 1851 TGCAATCAAAACCATTCATGGTGACAATGTACGTAAATATGAACCATTTT 1900

R 1901 GGAGTGTTATAGAAAGTCATTGGAACTCGTTGTTCTACCACCCGGTATAT 1950

||||||||||||||||||||||||||||||||||||||||||||||||||

S 1901 GGAGTGTTATAGAAAGTCATTGGAACTCGTTGTTCTACCACCCGGTATAT 1950

R 1951 GTAGCTGCTTACTACTTAAATCCATCATACAGATATCGACCTGATTTTAC 2000

||||||||||||||||||||||||||||||||||||||||||||||||||

S 1951 GTAGCTGCTTACTACTTAAATCCATCATACAGATATCGACCTGATTTTAC 2000

R 2001 GGCG--TACCGAGGGGATGCGTGGACTTAATGAGTGCATTGTTCGGCTGG 2048

|||| ||||||||||||||||||||||||||||||||||||||||||||

S 2001 GGCGCATACCGAGGGGATGCGTGGACTTAATGAGTGCATTGTTCGGCTGG 2050

R 2049 AGCCAGACAGTGCAAGAAGGATTTCTGCATCTATGCAG--TTCTGATTAC 2096

|||||||||||||||||||||||||||||||||||||| ||||||||||

S 2051 AGCCAGACAGTGCAAGAAGGATTTCTGCATCTATGCAGATTTCTGATTAC 2100

R 2097 AACTCTGCTAAAGCTGATTTTGGAACCGAATTGGCAATCAGTACAAGAAC 2146

||||||||||||||||||||||||||||||||||||||||||||||||||

S 2101 AACTCTGCTAAAGCTGATTTTGGAACCGAATTGGCAATCAGTACAAGAAC 2150

R 2147 TGAGCTTGATCCAGCTGCATGGTGGCAACAACATGGGATAAGTTGCTTAG 2196

||||||||||||||||||||||||||||||||||||||||||||||||||

S 2151 TGAGCTTGATCCAGCTGCATGGTGGCAACAACATGGGATAAGTTGCTTAG 2200

R 2197 AGCTGCAGCGTATTGCTGTCCGTATTTTGAGTCAGACGTGCTCATCTTTT 2246

||||||||||||||||||||||||||||||||||||||||||||||||||

S 2201 AGCTGCAGCGTATTGCTGTCCGTATTTTGAGTCAGACGTGCTCATCTTTT 2250

R 2247 GGTTGTGAGCATAACTGGAGTATATACGATCAATTGTACAGTTTAAGAAA 2296

||||||||||||||||||||||||||||||||||||||||||||||||||

S 2251 GGTTGTGAGCATAACTGGAGTATATACGATCAATTGTACAGTTTAAGAAA 2300

R 2297 TAATCGTTTAGCTCAAAAAAGATTGAATGACCTCATCTACGTTCACTACA 2346

||||||||||||||||||||||||||||||||||||||||||||||||||

S 2301 TAATCGTTTAGCTCAAAAAAGATTGAATGACCTCATCTACGTTCACTACA 2350

R 2347 ATCTGCGTCTTAGAGAACAACAATTACGAAGAAGGGCTGATAATTCAATC 2396

||||||||||||||||||||||||||||||||||||||||||||||||||

S 2351 ATCTGCGTCTTAGAGAACAACAATTACGAAGAAGGGCTGATAATTCAATC 2400

R 2397 TCCCTCGACAATATTCTGTTAGAGCGTTTGCTAGATGACTGGATTGTAGA 2446

|||||.||||||||||||||||||||||||||||||||||||||||||||

S 2401 TCCCTTGACAATATTCTGTTAGAGCGTTTGCTAGATGACTGGATTGTAGA 2450

R 2447 TGCCGCCGAAAATGACATGCTAGAAAATGAGGAAGTCCTTTACAATGAAA 2496

||||||||||||||||||||||||||||||||||||||||||||||||||

S 2451 TGCCGCCGAAAATGACATGCTAGAAAATGAGGAAGTCCTTTACAATGAAA 2500

R 2497 TCGAACAGGTTGATGAATACGAGAATGATATGGTTGATTATGAAGGTGTA 2546

||||||||||||||||||||||||||||||||||||||||||||||||||

S 2501 TCGAACAGGTTGATGAATACGAGAATGATATGGTTGATTATGAAGGTGTA 2550

R 2547 AATGGAAATGCAGAGACTAGGAACGGATCAGTCGAGCTGTTAACTTTGGC 2596

||||||||||||||||||||||||||||||||||||||||||||||||||

S 2551 AATGGAAATGCAGAGACTAGGAACGGATCAGTCGAGCTGTTAACTTTGGC 2600

R 2597 TGATGCAGATATAAATCCTGCCAATGCTGGTGTTGCCACTGATGATGATG 2646

||||||||||||||||||||||||||||||||||||||||||||||||||

S 2601 TGATGCAGATATAAATCCTGCCAATGCTGGTGTTGCCACTGATGATGATG 2650

R 2647 ATGAAGATGATGAGGATGGCGATATAAACTTTTTCGATGATGACATGAGT 2696

||||||||||||||||||||||||||||||||||||||||||||||||||

S 2651 ATGAAGATGATGAGGATGGCGATATAAACTTTTTCGATGATGACATGAGT 2700

R 2697 GATTAG 2702

||||||

S 2701 GATTAG 2706

**Pa13 - Structure-specific endonuclease subunit SLX1**

Aligned_sequences: 2

1: **Par.chr1R_long.5.98**

2: **Par.chr1S_long.6.134**

Matrix: EBLOSUM62

Gap_penalty: 10.0

Extend_penalty: 0.5

Length: 269

Identity: 52/269 (19.3%)

Similarity: 54/269 (20.1%)

Gaps: 213/269 (79.2%)

Score: 269.0

R 1 MGQRRKIGSEIPETLIEEEEETEEGRFFACYLLTSRSPRYKGHTYIGFTV 50

||||||||||||||||.|||||||||||||||||||||||||||||||||

S 1 MGQRRKIGSEIPETLIAEEEETEEGRFFACYLLTSRSPRYKGHTYIGFTV 50

R 51 NP-RMVK------------------------------------------- 56

|| |.::

S 51 NPRRRIRQHNGEIAQGAWRTKRKRPWEMVLCIYGFPTNVSALQFEWAWQH 100

R 57 -------------------------------------------------- 56

S 101 PTVSKAVRQAAASFKSLRGLVSKIKLAYTMLTLPPWQSLNITVKFFSTQY 150

R 57 -------------------------------------------------- 56

S 151 TKHSAGCPRLPEQMKVEVCSMDELPSCTKLSDDLLENKDEWCHERECDED 200

R 57 -------------------------------------------------- 56

S 201 MNSSTLPEETLLDFRTHNSADDQQSDSGIRMNEEYGCSKEVGKDEWYNGK 250

R 57 ------------------- 56

S 251 ECDEAMKDGTCQKKHDQIL 269

Aligned_sequences: 2

1: **Par.chr1R_long.5.98**

2: **Par.chr1S_long.6.134**

Matrix: EDNAFULL

Gap_penalty: 10.0

Extend_penalty: 0.5

Length: 810

Identity: 786/810 (97.0%)

Similarity: 786/810 (97.0%)

Gaps: 19/810 ( 2.3%)

Score: 3891.0

R 1 ATGGGGCAGAGAAGAAAGATTGGATCAGAAATCCCAGAAACCCTAATCGA 50

|||||||||||||||||||||||||||||||||||||||||||||||||.

S 1 ATGGGGCAGAGAAGAAAGATTGGATCAGAAATCCCAGAAACCCTAATCGC 50

R 51 GGAGGAGGAAGAAACAGAAGAAGGACGATTCTTCGCATGCTATCTGTTGA 100

||||||||||||||||||||||||||||||||||||||||||||||||||

S 51 GGAGGAGGAAGAAACAGAAGAAGGACGATTCTTCGCATGCTATCTGTTGA 100

R 101 CCTCTCGCAGCCCCCGCTACAAAGGCCACACCTATATCGGATTCACAGTG 150

||||||||||||||||||||||||||||||||||||||||||||||||||

S 101 CCTCTCGCAGCCCCCGCTACAAAGGCCACACCTATATCGGATTCACAGTG 150

R 151 AACCCACG-------------------CATGGTGAAATAGCGCAAGGTGC 181

|||||||| .||||||||||||||||||||||

S 151 AACCCACGGCGTCGTATAAGACAGCACAATGGTGAAATAGCGCAAGGTGC 200

R 182 TTGGAGAACGAAGCGGAAGCGTCCATGGGAGATGGTGTTGTGCATCTATG 231

||||||||||||||||||||||||||||||||||||||||||||||||||

S 201 TTGGAGAACGAAGCGGAAGCGTCCATGGGAGATGGTGTTGTGCATCTATG 250

R 232 GTTTCCCAACTAACGTTTCTGCTCTCCAGTTTGAATGGGCCTGGCAGCAC 281

||||||||||||||||||||||||||||||||||||||||||||||||||

S 251 GTTTCCCAACTAACGTTTCTGCTCTCCAGTTTGAATGGGCCTGGCAGCAC 300

R 282 CCAACTGTATCGAAGGCGGTTAGGCAGGCTGCTGCAAGCTTTAAATCACT 331

||||||||||||||||||||||||||||||||||||||||||||||||||

S 301 CCAACTGTATCGAAGGCGGTTAGGCAGGCTGCTGCAAGCTTTAAATCACT 350

R 332 GCGAGGGCTTGTCAGTAAGATCAAACTTGCATACACCATGCTCACTCTCC 381

||||||||||||||||||||||||||||||||||||||||||||||||||

S 351 GCGAGGGCTTGTCAGTAAGATCAAACTTGCATACACCATGCTCACTCTCC 400

R 382 CTCCTTGGCAGAGCTTGAACATCACTGTAAAATTCTTTTCAACCCAGTAC 431

||||||||||||||||||||||||||||||||||||||||||||||||||

S 401 CTCCTTGGCAGAGCTTGAACATCACTGTAAAATTCTTTTCAACCCAGTAC 450

R 432 ACCAAACATTCTGCTGGTTGTCCACGACTTCCAGAACAGATGAAGGTCAA 481

||||||||||||||||||||||||||||||||||||||||||||||||.|

S 451 ACCAAACATTCTGCTGGTTGTCCACGACTTCCAGAACAGATGAAGGTCGA 500

R 482 AGTCTGCTCAATGGATGAGCTTCCTTCCTGTACTAAACTATCTGATGACC 531

||||||||||||||||||||||||||||||||||||||||||||||||||

S 501 AGTCTGCTCAATGGATGAGCTTCCTTCCTGTACTAAACTATCTGATGACC 550

R 532 TTTTGGAAAATAAAGATGACTGGTGTCATGAAAGGGAATGTGATGAAGAT 581

|||||||||||||||||||.||||||||||||||||||||||||||||||

S 551 TTTTGGAAAATAAAGATGAGTGGTGTCATGAAAGGGAATGTGATGAAGAT 600

R 582 ATGAATTCCAGTACACTACCCGAAGAAACATTATTGGACTTCAGGACTCA 631

||||||||||||||||||||||||||||||||||||||||||||||||||

S 601 ATGAATTCCAGTACACTACCCGAAGAAACATTATTGGACTTCAGGACTCA 650

R 632 TAATTCAGCAGATGATCAGCAGAGTGATAGTGGCATTAGAATGAATGAAG 681

||||||||||||||||||||||||||||||||||||||||||||||||||

S 651 TAATTCAGCAGATGATCAGCAGAGTGATAGTGGCATTAGAATGAATGAAG 700

R 682 AATATGGATGCAGCAAGGAAGTAGGAAAAGATGAGTGGTATAATGGAAAG 731

||||||||||||||||||||||||||||||||||||||||||||||||||

S 701 AATATGGATGCAGCAAGGAAGTAGGAAAAGATGAGTGGTATAATGGAAAG 750

R 732 GAATGTGATGAAGCTATGAAAGATGGTACATGACAGAAGAAACATGATCA 781

||||||||||||||||||||||||||||||||.|||||||||||||||||

S 751 GAATGTGATGAAGCTATGAAAGATGGTACATGTCAGAAGAAACATGATCA 800

R 782 GATCTTATAA 791

||||||||||

S 801 GATCTTATAA 810

**Pa21 - pto-interacting protein 1-like**

Aligned_sequences: 2

1: **Par.chr1R_long.6.21**

2: **Par.chr1S_long.6.30**

Matrix: EBLOSUM62

Gap_penalty: 10.0

Extend_penalty: 0.5

Length: 379

Identity: 150/379 (39.6%)

Similarity: 152/379 (40.1%)

Gaps: 224/379 (59.1%)

Score: 760.0

R 1 MSCFSCCVQDDIRKASDNGPFVANNSAGSSGGYYHRETAPKDTQTVNILP 50

||||||||||||||||||||||||||||||||||||||||||||||||||

S 1 MSCFSCCVQDDIRKASDNGPFVANNSAGSSGGYYHRETAPKDTQTVNILP 50

R 51 IAVPAIPVDELKDLTDNFGTKSLIGEGSYGRVYHGVLKSGPAAAIKKLDS 100

||||||||||||||||||||||||||||||||||||||||||||||||||

S 51 IAVPAIPVDELKDLTDNFGTKSLIGEGSYGRVYHGVLKSGPAAAIKKLDS 100

R 101 SKQPDQEFLSQVSMVSRLKHENVVELVGYCIDGPLRLLAYEYAPNGSLMI 150

||||||||||||||||||||||||||||||||||||||||||||||||

S 101 SKQPDQEFLSQVSMVSRLKHENVVELVGYCIDGPLRLLAYEYAPNGSL-- 148

R 151 FSIKTFLLLIKLLISFTTVAYHLIINPP---------------------- 178

|.|::..

S 149 ---------------------HDILHGQKGVKGAQPGPVLSWVQRVKIAV 177

R 179 -------------------------------------------------- 178

S 178 GAARGLEYLHEKAQPHIIHRDIKSCNILLFDDDVAKIADFDLSNQAPDMA 227

R 179 -------------------------------------------------- 178

S 228 ARLHSTRVLGTFGYHAPEYAMTGQLSSKSDVYSFGVVLLELLTGRKPVDH 277

R 179 -------------------------------------------------- 178

S 278 TLPRGQQSLVTWATPKLSEDKVKQCVDARLNGEYPSKAVAKLAAVAALCV 327

R 179 ----------------------------- 178

S 328 QYEADFRPNMSIVVKALQPLLNARSGPHH 356

Aligned_sequences: 2

1: **Par.chr1R_long.6.21**

2: **Par.chr1S_long.6.30**

Matrix: EDNAFULL

Gap_penalty: 10.0

Extend_penalty: 0.5

Length: 1088

Identity: 507/1088 (46.6%)

Similarity: 507/1088 (46.6%)

Gaps: 568/1088 (52.2%)

Score: 2348.0

Green color: START CODON

Red color: STOP CODON

R 1 ATGAGCTGCTTCAGTTGTTGTGTACAAGATGATATCCGCAAAGCTTCTGA 50

||||||||||||||||||||||||||||||||||||||||||||||||||

S 1 ATGAGCTGCTTCAGTTGTTGTGTACAAGATGATATCCGCAAAGCTTCTGA 50

R 51 CAATGGACCGTTTGTAGCAAATAACTCAGCAGGCAGTAGTGGAGGTTACT 100

||||||||||||||||||||||||||||||||||||||||||||||||||

S 51 CAATGGACCGTTTGTAGCAAATAACTCAGCAGGCAGTAGTGGAGGTTACT 100

R 101 ATCACAGAGAAACTGCACCAAAGGATACTCAGACTGTAAATATCCTACCC 150

||||||||||||||||||||||||||||||||||||||||||||||||||

S 101 ATCACAGAGAAACTGCACCAAAGGATACTCAGACTGTAAATATCCTACCC 150

R 151 ATTGCTGTCCCTGCCATTCCAGTCGATGAATTAAAAGATTTGACAGATAA 200

||||||||||||||||||||||||||||||||||||||||||||||||||

S 151 ATTGCTGTCCCTGCCATTCCAGTCGATGAATTAAAAGATTTGACAGATAA 200

R 201 TTTTGGCACAAAGTCCTTAATTGGTGAGGGTTCATATGGAAGAGTATATC 250

||||||||||||||||||||||||||||||||||||||||||||||||||

S 201 TTTTGGCACAAAGTCCTTAATTGGTGAGGGTTCATATGGAAGAGTATATC 250

R 251 ATGGTGTTCTGAAAAGTGGGCCAGCTGCAGCTATTAAAAAGTTAGATTCC 300

||||||||||||||||||||||||||||||||||||||||||||||||||

S 251 ATGGTGTTCTGAAAAGTGGGCCAGCTGCAGCTATTAAAAAGTTAGATTCC 300

R 301 AGTAAACAACCTGACCAAGAATTTTTATCACAGGTCTCCATGGTTTCAAG 350

||||||||||||||||||||||||||||||||||||||||||||||||||

S 301 AGTAAACAACCTGACCAAGAATTTTTATCACAGGTCTCCATGGTTTCAAG 350

R 351 ACTAAAACATGAAAATGTTGTTGAGCTTGTTGGTTATTGTATTGATGGCC 400

||||||||||||||||||||||||||||||||||||||||||||||||||

S 351 ACTAAAACATGAAAATGTTGTTGAGCTTGTTGGTTATTGTATTGATGGCC 400

R 401 CTCTCCGTCTCCTTGCCTATGAGTATGCTCCTAATGGGTCAC-TCATGAT 449

|||||||||||||||||||||||||||||||||||||||||| |||||||

S 401 CTCTCCGTCTCCTTGCCTATGAGTATGCTCCTAATGGGTCACTTCATGAT 450

R 450 ATTCTCCA--------------TTAAG---------------ACATTTCT 470

|||||||| ||||| .||.||||

S 451 ATTCTCCATGGACAAAAAGGTGTTAAGGGAGCACAGCCAGGTCCAGTTCT 500

R 471 ------ACTGCTTA---TTAAACTTCTTATTTCATTCACCACTGTTGCCT 511

..|||..| |||||.|| .|||||| .

S 501 GTCATGGGTGCAAAGGGTTAAAATT---------------GCTGTTG--G 533

R 512 ATC---------ACTTGATTATTAAC--------------CCTC-CATAA 537

|.| ||||||.||||.|| |||| .||||

S 534 AGCAGCAAGAGGACTTGAATATTTACATGAAAAGGCTCAGCCTCATATAA 583

R 538 -------------------------------------------------- 537

S 584 TCCATCGTGATATTAAGTCCTGCAATATACTGCTTTTTGATGATGATGTT 633

R 538 -------------------------------------------------- 537

S 634 GCAAAGATTGCTGACTTTGATCTGTCAAATCAAGCCCCTGATATGGCTGC 683

R 538 -------------------------------------------------- 537

S 684 ACGTCTTCATTCCACTCGTGTTCTTGGGACTTTTGGTTATCATGCTCCAG 733

R 538 -------------------------------------------------- 537

S 734 AATATGCAATGACCGGACAGCTGAGTTCAAAAAGTGATGTTTACAGCTTT 783

R 538 -------------------------------------------------- 537

S 784 GGTGTAGTTCTGCTTGAACTCTTGACTGGGCGCAAACCTGTTGATCACAC 833

R 538 -------------------------------------------------- 537

S 834 ATTGCCACGTGGACAGCAGAGCCTTGTGACATGGGCAACACCGAAACTCA 883

R 538 -------------------------------------------------- 537

S 884 GTGAAGATAAGGTGAAGCAGTGTGTTGATGCTAGACTCAATGGAGAATAC 933

R 538 -------------------------------------------------- 537

S 934 CCTTCCAAGGCAGTTGCAAAGCTGGCTGCTGTTGCTGCCTTGTGCGTCCA 983

R 538 -------------------------------------------------- 537

S 984 ATATGAAGCTGATTTTCGGCCAAATATGAGCATTGTCGTCAAGGCTCTAC 1033

R 538 -------------------------------------- 537

S 1034 AGCCGCTATTGAATGCTCGGTCCGGCCCCCACCATTGA 1071

**Pa27/28 - Pleiotropic drug resistance protein 3-like**

Aligned_sequences: 2

1: **Par.chr1R_long.6.113_A**

2: **Par.chr1S_long.6.119**

Matrix: EBLOSUM62

Gap_penalty: 10.0

Extend_penalty: 0.5

Length: 1429

Identity: 732/1429 (51.2%)

Similarity: 732/1429 (51.2%)

Gaps: 696/1429 (48.7%)

Score: 3840.0

R 1 -------------------------------------------------- 0

S 1 MELTPVERNVHSIIGEEDEEVQLQWAAIERLPTLKRLRTSLFDVGGGGNG 50

R 1 -------------------------------------------------- 0

S 51 SGGKDSKDYAGKRVVDVTKLGAHERHLFIEKLISHIENDNLKLLQKLRER 100

R 1 -------------------------------------------------- 0

S 101 IDRVNVKLPTVEVRYKNLFVEAECEVVQGKPLPTLWNSLLSLLSVFTKAI 150

R 1 -------------------------------------------------- 0

S 151 WFKSVEAKISILTDVSGIIKPSRLTLLLGPPGCGKTTLLQALAGKQDKSL 200

R 1 -------------------------------------------------- 0

S 201 KVSGEISYNGHKLDEFVPQKTSAYISEYDLHIPELTVRETIDFSARCQGV 250

R 1 -------------------------------------------------- 0

S 251 RSRADIMMEVSRREKEAGIVPDPDIDTYMKAISVQGQKRNLQTDYILKIL 300

R 1 -------------------------------------------------- 0

S 301 GLDTCSDTMVGDALSRGISGGQKKRLTTGEMIVGPTKALFMDEISTGLDS 350

R 1 -------------------------------------------------- 0

S 351 STTFQIVTYLQQLVHITDATALVSLLQPAPETFDLFDDVILMGEGKVVYH 400

R 1 -------------------------------------------------- 0

S 401 GPRSHALQFFEDCGFKCPSRKGAADFLQEVISKKDQAQYWKHDNIPYYHV 450

R 1 -------------------------------------------------- 0

S 451 SVDQLSQLFRASYLGNKLDDELSKPYDKSQSHDNALSFTTYSVSKWELFK 500

R 1 -------------------------------------------------- 0

S 501 ACMARELLLMKRNSFVYVFKTVQLIIIAFITMTVFIRTQMAVNLTSANFL 550

R 1 -------------------------------------------------- 0

S 551 LGALFYTLVRHMTNGVAELSLTVTRLPVVYKQRGFYLYPAWAYSIPASML 600

R 1 -------------------------------------------------- 0

S 601 KVPFSFMDSVLWTATTYYVIGYSPEIKRFFCQFLVLFALHQASTSMCRLV 650

R 1 ----------------------------------------------MTYG 4

||||

S 651 AVIFRTMVAATTCGTFILVVMFLCGGFILPRPSLPPWLRWVFWCSPMTYG 700

R 5 EIGTALNEFLAPRWQKVSKGNTTLGNEVLTSHGLNFDGSFYWISVGALFG 54

||||||||||||||||||||||||||||||||||||||||||||||||||

S 701 EIGTALNEFLAPRWQKVSKGNTTLGNEVLTSHGLNFDGSFYWISVGALFG 750

R 55 FTVLFDLGFALALTYLNPPKMSRAIISEKRLSQLQGKDACNTSAQSENVS 104

||||||||||||||||||||||||||||||||||||||||||||||||||

S 751 FTVLFDLGFALALTYLNPPKMSRAIISEKRLSQLQGKDACNTSAQSENVS 800

R 105 TPADLYQNVGEKLKFGKMALALPFEPLTMSFKDVQYYVDTPPEMREHGFK 154

||||||||||||||||||||||||||||||||||||||||||||||||||

S 801 TPADLYQNVGEKLKFGKMALALPFEPLTMSFKDVQYYVDTPPEMREHGFK 850

R 155 QKKLQLLKDITGAFRPGILTALMGVSGAGKTTLMDVLSGRKTGGTIEGDI 204

||||||||||||||||||||||||||||||||||||||||||||||||||

S 851 QKKLQLLKDITGAFRPGILTALMGVSGAGKTTLMDVLSGRKTGGTIEGDI 900

R 205 RIGGHPKVQKTFARISGYCEQTDIHSPHITVGESVMYSAWLRLPPDTDPD 254

||||||||||||||||||||||||||||||||||||||||||||||||||

S 901 RIGGHPKVQKTFARISGYCEQTDIHSPHITVGESVMYSAWLRLPPDTDPD 950

R 255 TKSRFVEEVIETIELEDIKDSLVGIPGQSGLSTEQRKRLTIAVELVSNPS 304

||||||||||||||||||||||||||||||||||||||||||||||||||

S 951 TKSRFVEEVIETIELEDIKDSLVGIPGQSGLSTEQRKRLTIAVELVSNPS 1000

R 305 IIFMDEPTSGLDARAAAIVMRAVKNVVDTGRTTVCTIHQPSIDIFESFDE 354

||||||||||||||||||||||||||||||||||||||||||||||||||

S 1001 IIFMDEPTSGLDARAAAIVMRAVKNVVDTGRTTVCTIHQPSIDIFESFDE 1050

R 355 LILMKTGGQIIYSGILGHQSSKLIEYFEGIPGVPKIKDNYNPATWMLEVT 404

||||||||||||||||||||||||||||||||||||||||||||||||||

S 1051 LILMKTGGQIIYSGILGHQSSKLIEYFEGIPGVPKIKDNYNPATWMLEVT 1100

R 405 SASVEEELGLDFASIYRESTQYRDTIELVRQLIVPKPGSKDLYFPTHFPQ 454

||||||||||||||||||||||||||||||||.|||||||||||||||||

S 1101 SASVEEELGLDFASIYRESTQYRDTIELVRQLSVPKPGSKDLYFPTHFPQ 1150

R 455 NSWVQFKACLWKQHLSYWRSPEYNLARFMFMIGASVLFGIIFWQKGKEIN 504

||||||||||||||||||||||||||||||||||||||||||||||||||

S 1151 NSWVQFKACLWKQHLSYWRSPEYNLARFMFMIGASVLFGIIFWQKGKEIN 1200

R 505 NEQDLLNILGSMYIAVIFLGVTNCNLVLPYVETERTVLYRERFAGMYSSK 554

||||||||||||||||||||||||||||||||||||||||||||||||||

S 1201 NEQDLLNILGSMYIAVIFLGVTNCNLVLPYVETERTVLYRERFAGMYSSK 1250

R 555 AYSFAQVAVEMPYTLLQAILFVIITYPTIGYYWSATKVFWYFYATFWTFL 604

||||||||||||||||||||||||||||||||||||||||||||||||||

S 1251 AYSFAQVAVEMPYTLLQAILFVIITYPTIGYYWSATKVFWYFYATFWTFL 1300

R 605 YFVYLGMLIASLSTNLDVASILATAVYTILNLFSGFLMPGPKIPKWWVWC 654

||||||||||||||||||||||||||||||||||||||||||||||||||

S 1301 YFVYLGMLIASLSTNLDVASILATAVYTILNLFSGFLMPGPKIPKWWVWC 1350

R 655 YWICPTSWSLNGLLTSQYGDMNKEILIFGEHKTVGSFLQDYYGFHHDGLA 704

||||||||||||||||||||||||||||||||||||||||||||||||||

S 1351 YWICPTSWSLNGLLTSQYGDMNKEILIFGEHKTVGSFLQDYYGFHHDGLA 1400

R 705 LVAIVLIAFPVAYASLFAYCIGKLNFQRR 733

|||||||||||||||||||||||||||||

S 1401 LVAIVLIAFPVAYASLFAYCIGKLNFQRR 1429

Aligned_sequences: 2

1: **Par.chr1R_long.6.113_A**

2: **Par.chr1S_long.6.119**

Matrix: EDNAFULL

Gap_penalty: 10.0

Extend_penalty: 0.5

Length: 4290

Identity: 2200/4290 (51.3%)

Similarity: 2200/4290 (51.3%)

Gaps: 2088/4290 (48.7%)

Score: 10992.0

Green color: START CODON

Red color: STOP CODON

R 1 -------------------------------------------------- 0

S 1 ATGGAGCTAACACCAGTTGAAAGAAATGTCCACTCAATCATAGGGGAGGA 50

R 1 -------------------------------------------------- 0

S 51 GGATGAGGAAGTTCAGCTGCAGTGGGCTGCAATTGAGAGACTGCCTACGT 100

R 1 -------------------------------------------------- 0

S 101 TAAAACGCCTCAGAACCTCACTTTTTGATGTTGGTGGTGGTGGGAATGGA 150

R 1 -------------------------------------------------- 0

S 151 AGCGGTGGAAAAGACTCGAAAGACTATGCAGGAAAGAGGGTGGTTGATGT 200

R 1 -------------------------------------------------- 0

S 201 TACTAAGTTAGGAGCACATGAGAGGCATCTGTTCATTGAAAAACTCATAA 250

R 1 -------------------------------------------------- 0

S 251 GCCACATAGAGAATGATAATCTGAAACTTCTACAGAAACTGCGAGAAAGA 300

R 1 -------------------------------------------------- 0

S 301 ATAGACCGAGTGAATGTGAAACTGCCGACAGTGGAGGTGAGGTACAAGAA 350

R 1 -------------------------------------------------- 0

S 351 CTTGTTTGTGGAAGCAGAGTGTGAGGTAGTCCAAGGAAAGCCGCTTCCGA 400

R 1 -------------------------------------------------- 0

S 401 CGCTATGGAACTCTCTTCTAAGCTTGTTATCAGTTTTTACAAAGGCAATT 450

R 1 -------------------------------------------------- 0

S 451 TGGTTCAAATCTGTAGAAGCCAAGATAAGCATTCTAACAGATGTCAGTGG 500

R 1 -------------------------------------------------- 0

S 501 CATCATCAAACCATCAAGGCTTACTCTCCTTCTTGGTCCCCCAGGCTGTG 550

R 1 -------------------------------------------------- 0

S 551 GGAAAACCACCTTGTTACAAGCTCTTGCAGGAAAACAAGACAAATCTCTC 600

R 1 -------------------------------------------------- 0

S 601 AAGGTTTCAGGGGAGATTTCTTATAATGGTCACAAGCTAGATGAGTTTGT 650

R 1 -------------------------------------------------- 0

S 651 CCCCCAGAAAACGTCAGCTTACATAAGCGAATACGACCTTCACATACCTG 700

R 1 -------------------------------------------------- 0

S 701 AGTTGACTGTGAGGGAAACAATCGACTTTTCTGCCCGCTGTCAGGGTGTT 750

R 1 -------------------------------------------------- 0

S 751 CGGAGTAGAGCTGATATAATGATGGAGGTCAGCAGAAGAGAAAAAGAAGC 800

R 1 -------------------------------------------------- 0

S 801 AGGAATAGTCCCTGATCCAGATATTGACACCTACATGAAGGCAATATCAG 850

R 1 -------------------------------------------------- 0

S 851 TTCAAGGACAAAAGAGAAATCTCCAAACCGATTACATTTTGAAGATCCTC 900

R 1 -------------------------------------------------- 0

S 901 GGACTGGATACCTGTAGTGACACAATGGTTGGTGATGCATTAAGTAGAGG 950

R 1 -------------------------------------------------- 0

S 951 CATTTCAGGTGGCCAGAAGAAGAGGCTGACAACAGGAGAGATGATTGTAG 1000

R 1 -------------------------------------------------- 0

S 1001 GTCCCACAAAAGCTCTATTTATGGATGAAATATCGACGGGATTAGACAGC 1050

R 1 -------------------------------------------------- 0

S 1051 TCTACAACATTTCAGATAGTTACCTATCTCCAGCAGTTGGTGCACATCAC 1100

R 1 -------------------------------------------------- 0

S 1101 AGATGCAACTGCATTGGTATCACTTCTTCAACCAGCACCTGAGACCTTTG 1150

R 1 -------------------------------------------------- 0

S 1151 ATCTGTTTGATGATGTAATATTAATGGGAGAGGGGAAGGTAGTTTACCAT 1200

R 1 -------------------------------------------------- 0

S 1201 GGTCCTCGCAGCCACGCACTTCAGTTTTTTGAAGATTGCGGTTTCAAGTG 1250

R 1 -------------------------------------------------- 0

S 1251 CCCATCAAGAAAGGGTGCTGCAGATTTCCTTCAGGAGGTAATCTCAAAGA 1300

R 1 -------------------------------------------------- 0

S 1301 AGGATCAAGCACAATACTGGAAGCATGATAACATTCCCTACTATCATGTT 1350

R 1 -------------------------------------------------- 0

S 1351 TCAGTGGATCAGTTATCACAACTTTTCAGAGCAAGTTACTTAGGAAATAA 1400

R 1 -------------------------------------------------- 0

S 1401 GTTAGACGATGAGCTCTCAAAACCGTATGATAAATCGCAGTCCCATGACA 1450

R 1 -------------------------------------------------- 0

S 1451 ATGCCTTATCATTTACCACTTACTCTGTGAGCAAATGGGAGTTGTTCAAA 1500

R 1 -------------------------------------------------- 0

S 1501 GCTTGCATGGCCAGGGAGCTGCTTCTTATGAAACGAAATTCCTTTGTTTA 1550

R 1 -------------------------------------------------- 0

S 1551 TGTATTCAAAACAGTGCAGCTTATCATCATTGCATTTATTACAATGACAG 1600

R 1 -------------------------------------------------- 0

S 1601 TATTTATAAGGACTCAGATGGCTGTGAATTTGACAAGCGCAAATTTTTTG 1650

R 1 -------------------------------------------------- 0

S 1651 TTGGGCGCATTGTTTTATACACTTGTTCGACATATGACAAATGGTGTTGC 1700

R 1 -------------------------------------------------- 0

S 1701 AGAGCTGTCCTTGACTGTTACTAGACTTCCAGTAGTTTACAAGCAAAGAG 1750

R 1 -------------------------------------------------- 0

S 1751 GATTCTATTTGTACCCAGCATGGGCGTATTCTATTCCGGCCTCTATGCTG 1800

R 1 -------------------------------------------------- 0

S 1801 AAGGTTCCATTTTCATTCATGGATTCAGTGCTTTGGACCGCCACAACTTA 1850

R 1 -------------------------------------------------- 0

S 1851 CTATGTTATTGGGTATAGCCCAGAAATAAAAAGGTTCTTCTGCCAGTTCC 1900

R 1 -------------------------------------------------- 0

S 1901 TTGTGCTATTTGCTCTGCATCAAGCATCAACATCCATGTGTCGTTTGGTT 1950

R 1 -------------------------------------------------- 0

S 1951 GCTGTAATTTTCCGAACTATGGTTGCTGCAACAACTTGTGGTACTTTTAT 2000

R 1 -------------------------------------------------- 0

S 2001 CTTAGTGGTAATGTTTTTATGTGGAGGCTTCATTTTGCCACGACCCTCTC 2050

R 1 --------------------------------------ATGACTTATGGG 12

||||||||||||

S 2051 TACCTCCGTGGTTGAGGTGGGTATTCTGGTGTTCTCCTATGACTTATGGG 2100

R 13 GAAATAGGTACAGCTCTAAATGAATTCCTTGCTCCTCGCTGGCAAAAGGT 62

||||||||||||||||||||||||||||||||||||||||||||||||||

S 2101 GAAATAGGTACAGCTCTAAATGAATTCCTTGCTCCTCGCTGGCAAAAGGT 2150

R 63 TTCAAAGGGAAACACAACCTTAGGGAATGAAGTTCTAACCAGTCATGGTC 112

||||||||||||||||||||||||||||||||||||||||||||||||||

S 2151 TTCAAAGGGAAACACAACCTTAGGGAATGAAGTTCTAACCAGTCATGGTC 2200

R 113 TGAACTTTGATGGCTCTTTCTATTGGATATCGGTAGGAGCATTATTCGGC 162

||||||||||||||||||||||||||||||||||||||||||||||||||

S 2201 TGAACTTTGATGGCTCTTTCTATTGGATATCGGTAGGAGCATTATTCGGC 2250

R 163 TTCACAGTACTTTTCGATCTTGGATTTGCTTTAGCCTTAACTTACTTAAA 212

||||||||||||||||||||||||||||||||||||||||||||||||||

S 2251 TTCACAGTACTTTTCGATCTTGGATTTGCTTTAGCCTTAACTTACTTAAA 2300

R 213 TCCTCCAAAGATGTCTCGGGCTATTATTTCGGAAAAGAGGTTATCTCAAC 262

||||||||||||||||||||||||||||||||||||||||||||||||||

S 2301 TCCTCCAAAGATGTCTCGGGCTATTATTTCGGAAAAGAGGTTATCTCAAC 2350

R 263 TACAAGGGAAAGATGCTTGCAACACCAGTGCTCAGTCAGAGAATGTATCA 312

||||||||||||||||||||||||||||||||||||||||||||||||||

S 2351 TACAAGGGAAAGATGCTTGCAACACCAGTGCTCAGTCAGAGAATGTATCA 2400

R 313 ACTCCAGCTGATCTTTACCAAAATGTGGGAGAAAAGTTAAAATTTGGGAA 362

||||||||||||||||||||||||||||||||||||||||||||||||||

S 2401 ACTCCAGCTGATCTTTACCAAAATGTGGGAGAAAAGTTAAAATTTGGGAA 2450

R 363 GATGGCCCTGGCTCTGCCATTTGAACCACTGACAATGTCATTTAAGGATG 412

||||||||||||||||||||||||||||||||||||||||||||||||||

S 2451 GATGGCCCTGGCTCTGCCATTTGAACCACTGACAATGTCATTTAAGGATG 2500

R 413 TGCAGTATTATGTTGATACCCCTCCGGAAATGAGAGAGCATGGTTTCAAG 462

||||||||||||||||||||||||||||||||||||||||||||||||||

S 2501 TGCAGTATTATGTTGATACCCCTCCGGAAATGAGAGAGCATGGTTTCAAG 2550

R 463 CAGAAAAAGCTTCAGTTGCTTAAAGATATTACAGGAGCATTCAGACCTGG 512

||||||||||||||||||||||||||||||||||||||||||||||||||

S 2551 CAGAAAAAGCTTCAGTTGCTTAAAGATATTACAGGAGCATTCAGACCTGG 2600

R 513 AATTCTTACAGCATTGATGGGTGTCAGTGGAGCTGGGAAAACAACTCTCA 562

||||||||||||||||||||||||||||||||||||||||||||||||||

S 2601 AATTCTTACAGCATTGATGGGTGTCAGTGGAGCTGGGAAAACAACTCTCA 2650

R 563 TGGATGTTCTTTCTGGAAGGAAAACAGGAGGTACTATTGAAGGAGATATA 612

||||||||||||||||||||||||||||||||||||||||||||||||||

S 2651 TGGATGTTCTTTCTGGAAGGAAAACAGGAGGTACTATTGAAGGAGATATA 2700

R 613 AGAATAGGAGGGCACCCCAAGGTCCAGAAGACATTTGCAAGAATATCAGG 662

||||||||||||||||||||||||||||||||||||||||||||||||||

S 2701 AGAATAGGAGGGCACCCCAAGGTCCAGAAGACATTTGCAAGAATATCAGG 2750

R 663 TTACTGTGAGCAGACTGATATACATTCTCCACATATCACAGTAGGAGAAT 712

||||||||||||||||||||||||||||||||||||||||||||||||||

S 2751 TTACTGTGAGCAGACTGATATACATTCTCCACATATCACAGTAGGAGAAT 2800

R 713 CAGTTATGTACTCAGCTTGGTTGCGGTTGCCACCTGACACTGATCCAGAC 762

||||||||||||||||||||||||||||||||||||||||||||||||||

S 2801 CAGTTATGTACTCAGCTTGGTTGCGGTTGCCACCTGACACTGATCCAGAC 2850

R 763 ACAAAATCTAGATTTGTGGAAGAAGTCATTGAAACGATTGAACTGGAGGA 812

||||||||||||||||||||||||||||||||||||||||||||||||||

S 2851 ACAAAATCTAGATTTGTGGAAGAAGTCATTGAAACGATTGAACTGGAGGA 2900

R 813 TATAAAAGATTCTTTAGTTGGGATTCCTGGACAAAGTGGCCTATCCACTG 862

||||||||||||||||||||||||||||||||||||||||||||||||||

S 2901 TATAAAAGATTCTTTAGTTGGGATTCCTGGACAAAGTGGCCTATCCACTG 2950

R 863 AGCAGCGTAAAAGGCTAACAATTGCAGTGGAGCTTGTTTCCAATCCATCC 912

||||||||||||||||||||||||||||||||||||||||||||||||||

S 2951 AGCAGCGTAAAAGGCTAACAATTGCAGTGGAGCTTGTTTCCAATCCATCC 3000

R 913 ATAATATTTATGGATGAACCTACATCAGGTTTAGACGCCAGAGCAGCTGC 962

||||||||||||||||||||||||||||||||||||||||||||||||||

S 3001 ATAATATTTATGGATGAACCTACATCAGGTTTAGACGCCAGAGCAGCTGC 3050

R 963 AATTGTCATGCGCGCAGTGAAGAACGTCGTTGACACAGGAAGGACAACTG 1012

||||||||||||||||||||||||||||||||||||||||||||||||||

S 3051 AATTGTCATGCGCGCAGTGAAGAACGTCGTTGACACAGGAAGGACAACTG 3100

R 1013 TTTGCACGATTCACCAACCAAGCATTGATATCTTTGAGTCTTTTGATGAG 1062

||||||||||||||||||||||||||||||||||||||||||||||||||

S 3101 TTTGCACGATTCACCAACCAAGCATTGATATCTTTGAGTCTTTTGATGAG 3150

R 1063 TTAATTTTGATGAAAACGGGAGGACAGATCATCTATTCTGGAATATTAGG 1112

||||||||||||||||||||||||||||||||||||||||||||||||||

S 3151 TTAATTTTGATGAAAACGGGAGGACAGATCATCTATTCTGGAATATTAGG 3200

R 1113 ACATCAATCGAGTAAACTAATCGAATATTTCGAGGGCATTCCTGGTGTAC 1162

||||||||||||||||||||||||||||||||||||||||||||||||||

S 3201 ACATCAATCGAGTAAACTAATCGAATATTTCGAGGGCATTCCTGGTGTAC 3250

R 1163 CAAAAATCAAAGATAATTACAATCCAGCAACATGGATGTTAGAAGTTACT 1212

||||||||||||||||||||||||||||||||||||||||||||||||||

S 3251 CAAAAATCAAAGATAATTACAATCCAGCAACATGGATGTTAGAAGTTACT 3300

R 1213 TCCGCTTCGGTAGAGGAAGAACTTGGTTTAGATTTTGCCAGCATTTATAG 1262

||||||||||||||||||||||||||||||||||||||||||||||||||

S 3301 TCCGCTTCGGTAGAGGAAGAACTTGGTTTAGATTTTGCCAGCATTTATAG 3350

R 1263 AGAGTCTACTCAGTATAGGGACACAATTGAGCTGGTAAGACAGTTAATTG 1312

|||||||||||||||||||||||||||||||||||||||||||||||..|

S 3351 AGAGTCTACTCAGTATAGGGACACAATTGAGCTGGTAAGACAGTTAAGCG 3400

R 1313 TGCCAAAGCCGGGTTCAAAGGACTTGTATTTTCCCACTCATTTTCCTCAA 1362

||||||||||||||||||||||||||||||||||||||||||||||||||

S 3401 TGCCAAAGCCGGGTTCAAAGGACTTGTATTTTCCCACTCATTTTCCTCAA 3450

R 1363 AATAGCTGGGTGCAGTTTAAGGCATGCCTTTGGAAACAACACTTGTCCTA 1412

||||||||||||||||||||||||||||||||||||||||||||||||||

S 3451 AATAGCTGGGTGCAGTTTAAGGCATGCCTTTGGAAACAACACTTGTCCTA 3500

R 1413 CTGGAGAAGTCCTGAATACAATCTAGCACGTTTCATGTTTATGATTGGTG 1462

||||||||||||||||||||||||||||||||||||||||||||||||||

S 3501 CTGGAGAAGTCCTGAATACAATCTAGCACGTTTCATGTTTATGATTGGTG 3550

R 1463 CATCAGTGTTGTTTGGGATAATCTTTTGGCAGAAAGGGAAGGAAATAAAT 1512

||||||||||||||||||||||||||||||||||||||||||||||||||

S 3551 CATCAGTGTTGTTTGGGATAATCTTTTGGCAGAAAGGGAAGGAAATAAAT 3600

R 1513 AATGAGCAGGATTTGTTGAACATACTTGGGTCCATGTACATTGCCGTAAT 1562

||||||||||||||||||||||||||||||||||||||||||||||||||

S 3601 AATGAGCAGGATTTGTTGAACATACTTGGGTCCATGTACATTGCCGTAAT 3650

R 1563 ATTCTTGGGCGTAACCAATTGCAACTTAGTTCTGCCTTATGTGGAAACTG 1612

||||||||||||||||||||||||||||||||||||||||||||||||||

S 3651 ATTCTTGGGCGTAACCAATTGCAACTTAGTTCTGCCTTATGTGGAAACTG 3700

R 1613 AGCGCACTGTTTTGTACCGGGAACGATTTGCTGGGATGTACTCATCAAAG 1662

||||||||||||||||||||||||||||||||||||||||||||||||||

S 3701 AGCGCACTGTTTTGTACCGGGAACGATTTGCTGGGATGTACTCATCAAAG 3750

R 1663 GCTTATTCGTTTGCGCAGGTGGCCGTGGAAATGCCATACACATTGTTGCA 1712

||||||||||||||||||||||||||||||||||||||||||||||||||

S 3751 GCTTATTCGTTTGCGCAGGTGGCCGTGGAAATGCCATACACATTGTTGCA 3800

R 1713 AGCAATTCTGTTTGTGATTATAACATACCCTACAATAGGGTATTATTGGT 1762

||||||||||||||||||||||||||||||||||||||||||||||||||

S 3801 AGCAATTCTGTTTGTGATTATAACATACCCTACAATAGGGTATTATTGGT 3850

R 1763 CAGCTACTAAGGTTTTTTGGTACTTTTATGCAACATTCTGGACATTTCTG 1812

||||||||||||||||||||||||||||||||||||||||||||||||||

S 3851 CAGCTACTAAGGTTTTTTGGTACTTTTATGCAACATTCTGGACATTTCTG 3900

R 1813 TACTTTGTGTATCTTGGGATGCTGATTGCTTCTTTGAGCACAAACCTGGA 1862

||||||||||||||||||||||||||||||||||||||||||||||||||

S 3901 TACTTTGTGTATCTTGGGATGCTGATTGCTTCTTTGAGCACAAACCTGGA 3950

R 1863 TGTTGCTTCCATATTGGCAACTGCAGTTTACACCATATTGAATCTTTTCT 1912

||||||||||||||||||||||||||||||||||||||||||||||||||

S 3951 TGTTGCTTCCATATTGGCAACTGCAGTTTACACCATATTGAATCTTTTCT 4000

R 1913 CGGGCTTCCTCATGCCGGGACCGAAAATTCCTAAGTGGTGGGTTTGGTGC 1962

||||||||||||||||||||||||||||||||||||||||||||||||||

S 4001 CGGGCTTCCTCATGCCGGGACCGAAAATTCCTAAGTGGTGGGTTTGGTGC 4050

R 1963 TATTGGATATGTCCTACATCATGGTCCCTAAATGGCCTCCTGACCTCACA 2012

||||||||||||||||||||||||||||||||||||||||||||||||||

S 4051 TATTGGATATGTCCTACATCATGGTCCCTAAATGGCCTCCTGACCTCACA 4100

R 2013 ATATGGAGACATGAACAAAGAGATCTTGATTTTTGGGGAGCATAAAACAG 2062

||||||||||||||||||||||||||||||||||||||||||||||||||

S 4101 ATATGGAGACATGAACAAAGAGATCTTGATTTTTGGGGAGCATAAAACAG 4150

R 2063 TTGGTTCCTTCCTACAAGATTACTATGGTTTCCATCATGATGGTTTAGCC 2112

||||||||||||||||||||||||||||||||||||||||||||||||||

S 4151 TTGGTTCCTTCCTACAAGATTACTATGGTTTCCATCATGATGGTTTAGCC 4200

R 2113 CTTGTGGCTATTGTTCTCATTGCTTTCCCAGTTGCTTATGCGTCTCTATT 2162

||||||||||||||||||||||||||||||||||||||||||||||||||

S 4201 CTTGTGGCTATTGTTCTCATTGCTTTCCCAGTTGCTTATGCGTCTCTATT 4250

R 2163 TGCCTATTGCATTGGGAAATTAAATTTTCAAAGAAGGTAG 2202

||||||||||||||||||||||||||||||||||||||||

S 4251 TGCCTATTGCATTGGGAAATTAAATTTTCAAAGAAGGTAG 4290

Aligned_sequences: 2

1: **Par.chr1R_long.6.113_B**

2: **Par.chr1S_long.6.119**

Matrix: EBLOSUM62

Gap_penalty: 10.0

Extend_penalty: 0.5

Length: 1429

Identity: 522/1429 (36.5%)

Similarity: 523/1429 (36.6%)

Gaps: 902/1429 (63.1%)

Score: 2677.0

R 1 MELTPVERNVHSIIGEEDEEVQLQWAAIERLPTLKRLRTSLFDVGGGGNG 50

||||||||||||||||||||||||||||||||||||||||||||||||||

S 1 MELTPVERNVHSIIGEEDEEVQLQWAAIERLPTLKRLRTSLFDVGGGGNG 50

R 51 SGGKDSKDYAGKRVVDVTKLGAHERHLFIEKLISHIENDNLKLLQKLRER 100

||||||||||||||||||||||||||||||||||||||||||||||||||

S 51 SGGKDSKDYAGKRVVDVTKLGAHERHLFIEKLISHIENDNLKLLQKLRER 100

R 101 IDRVNVKLPTVEVRYKNLFVEAECEVVQGKPLPTLWNSLLSLLSVFTKAI 150

||||||||||||||||||||||||||||||||||||||||||||||||||

S 101 IDRVNVKLPTVEVRYKNLFVEAECEVVQGKPLPTLWNSLLSLLSVFTKAI 150

R 151 WFKSVEAKISILTDVSGIIKPSRLTLLLGPPGCGKTTLLQALAGKQDKSL 200

||||||||||||||||||||||||||||||||||||||||||||||||||

S 151 WFKSVEAKISILTDVSGIIKPSRLTLLLGPPGCGKTTLLQALAGKQDKSL 200

R 201 KVSGEISYNGHKLDEFVPQKTSAYISEYDLHIPELTVRETIDFSARCQGV 250

||||||||||||||||||||||||||||||||||||||||||||||||||

S 201 KVSGEISYNGHKLDEFVPQKTSAYISEYDLHIPELTVRETIDFSARCQGV 250

R 251 RSRADIMMEVSRREKEAGIVPDPDIDTYMKAISVQGQKRNLQTDYILKIL 300

||||||||||||||||||||||||||||||||||||||||||||||||||

S 251 RSRADIMMEVSRREKEAGIVPDPDIDTYMKAISVQGQKRNLQTDYILKIL 300

R 301 GLDTCSDTMVGDALSRGISGGQKKRLTTGEMIVGPTKALFMDEISTGLDS 350

||||||||||||||||||||||||||||||||||||||||||||||||||

S 301 GLDTCSDTMVGDALSRGISGGQKKRLTTGEMIVGPTKALFMDEISTGLDS 350

R 351 STTFQIVTYLQQLVHITDATALVSLLQPAPETFDLFDDVILMGEGKVVYH 400

||||||||||||||||||||||||||||||||||||||||||||||||||

S 351 STTFQIVTYLQQLVHITDATALVSLLQPAPETFDLFDDVILMGEGKVVYH 400

R 401 GPRSHTLQFFEDCGFKCPSRKGAADFLQEVISKKDQAQYWKHDNIPYYHV 450

|||||.||||||||||||||||||||||||||||||||||||||||||||

S 401 GPRSHALQFFEDCGFKCPSRKGAADFLQEVISKKDQAQYWKHDNIPYYHV 450

R 451 SVDQLSQLFRASYLGNKLDDELSKPYDKSQSHDNALSFTTYSVSKWELFK 500

||||||||||||||||||||||||||||||||||||||||||||||||||

S 451 SVDQLSQLFRASYLGNKLDDELSKPYDKSQSHDNALSFTTYSVSKWELFK 500

R 501 ACMARELLLMKRNSFVYVFKTVQVYCH----------------------- 527

|||||||||||||||||||||||:...

S 501 ACMARELLLMKRNSFVYVFKTVQLIIIAFITMTVFIRTQMAVNLTSANFL 550

R 528 -------------------------------------------------- 527

S 551 LGALFYTLVRHMTNGVAELSLTVTRLPVVYKQRGFYLYPAWAYSIPASML 600

R 528 -------------------------------------------------- 527

S 601 KVPFSFMDSVLWTATTYYVIGYSPEIKRFFCQFLVLFALHQASTSMCRLV 650

R 528 -------------------------------------------------- 527

S 651 AVIFRTMVAATTCGTFILVVMFLCGGFILPRPSLPPWLRWVFWCSPMTYG 700

R 528 -------------------------------------------------- 527

S 701 EIGTALNEFLAPRWQKVSKGNTTLGNEVLTSHGLNFDGSFYWISVGALFG 750

R 528 -------------------------------------------------- 527

S 751 FTVLFDLGFALALTYLNPPKMSRAIISEKRLSQLQGKDACNTSAQSENVS 800

R 528 -------------------------------------------------- 527

S 801 TPADLYQNVGEKLKFGKMALALPFEPLTMSFKDVQYYVDTPPEMREHGFK 850

R 528 -------------------------------------------------- 527

S 851 QKKLQLLKDITGAFRPGILTALMGVSGAGKTTLMDVLSGRKTGGTIEGDI 900

R 528 -------------------------------------------------- 527

S 901 RIGGHPKVQKTFARISGYCEQTDIHSPHITVGESVMYSAWLRLPPDTDPD 950

R 528 -------------------------------------------------- 527

S 951 TKSRFVEEVIETIELEDIKDSLVGIPGQSGLSTEQRKRLTIAVELVSNPS 1000

R 528 -------------------------------------------------- 527

S 1001 IIFMDEPTSGLDARAAAIVMRAVKNVVDTGRTTVCTIHQPSIDIFESFDE 1050

R 528 -------------------------------------------------- 527

S 1051 LILMKTGGQIIYSGILGHQSSKLIEYFEGIPGVPKIKDNYNPATWMLEVT 1100

R 528 -------------------------------------------------- 527

S 1101 SASVEEELGLDFASIYRESTQYRDTIELVRQLSVPKPGSKDLYFPTHFPQ 1150

R 528 -------------------------------------------------- 527

S 1151 NSWVQFKACLWKQHLSYWRSPEYNLARFMFMIGASVLFGIIFWQKGKEIN 1200

R 528 -------------------------------------------------- 527

S 1201 NEQDLLNILGSMYIAVIFLGVTNCNLVLPYVETERTVLYRERFAGMYSSK 1250

R 528 -------------------------------------------------- 527

S 1251 AYSFAQVAVEMPYTLLQAILFVIITYPTIGYYWSATKVFWYFYATFWTFL 1300

R 528 -------------------------------------------------- 527

S 1301 YFVYLGMLIASLSTNLDVASILATAVYTILNLFSGFLMPGPKIPKWWVWC 1350

R 528 -------------------------------------------------- 527

S 1351 YWICPTSWSLNGLLTSQYGDMNKEILIFGEHKTVGSFLQDYYGFHHDGLA 1400

R 528 ----------------------------- 527

S 1401 LVAIVLIAFPVAYASLFAYCIGKLNFQRR 1429

Aligned_sequences: 2

1: **Par.chr1R_long.6.113_B**

2: **Par.chr1S_long.6.119**

Matrix: EDNAFULL

Gap_penalty: 10.0

Extend_penalty: 0.5

Length: 4291

Identity: 1576/4291 (36.7%)

Similarity: 1576/4291 (36.7%)

Gaps: 2708/4291 (63.1%)

Score: 7825.5

Green color: START CODON

Red color: STOP CODON

R 1 ATGGAGCTAACACCAGTTGAAAGAAATGTCCACTCAATCATAGGGGAGGA 50

||||||||||||||||||||||||||||||||||||||||||||||||||

S 1 ATGGAGCTAACACCAGTTGAAAGAAATGTCCACTCAATCATAGGGGAGGA 50

R 51 GGATGAGGAAGTTCAGCTGCAGTGGGCTGCAATTGAGAGACTGCCTACAT 100

||||||||||||||||||||||||||||||||||||||||||||||||.|

S 51 GGATGAGGAAGTTCAGCTGCAGTGGGCTGCAATTGAGAGACTGCCTACGT 100

R 101 TAAAACGCCTCAGAACCTCACTTTTTGATGTTGGTGGTGGTGGGAATGGA 150

||||||||||||||||||||||||||||||||||||||||||||||||||

S 101 TAAAACGCCTCAGAACCTCACTTTTTGATGTTGGTGGTGGTGGGAATGGA 150

R 151 AGCGGTGGAAAAGACTCGAAAGACTATGCAGGGAAGAGGGTGGTTGATGT 200

||||||||||||||||||||||||||||||||.|||||||||||||||||

S 151 AGCGGTGGAAAAGACTCGAAAGACTATGCAGGAAAGAGGGTGGTTGATGT 200

R 201 TACTAAGTTAGGAGCACATGAGAGGCATCTGTTCATTGAAAAACTCATAA 250

||||||||||||||||||||||||||||||||||||||||||||||||||

S 201 TACTAAGTTAGGAGCACATGAGAGGCATCTGTTCATTGAAAAACTCATAA 250

R 251 GCCACATAGAGAATGATAATCTGAAACTTCTACAGAAACTGCGAGAAAGA 300

||||||||||||||||||||||||||||||||||||||||||||||||||

S 251 GCCACATAGAGAATGATAATCTGAAACTTCTACAGAAACTGCGAGAAAGA 300

R 301 ATAGACCGAGTGAATGTGAAACTGCCGACAGTGGAGGTGAGGTACAAGAA 350

||||||||||||||||||||||||||||||||||||||||||||||||||

S 301 ATAGACCGAGTGAATGTGAAACTGCCGACAGTGGAGGTGAGGTACAAGAA 350

R 351 CTTGTTTGTGGAAGCAGAGTGTGAGGTAGTCCAAGGAAAGCCGCTTCCGA 400

||||||||||||||||||||||||||||||||||||||||||||||||||

S 351 CTTGTTTGTGGAAGCAGAGTGTGAGGTAGTCCAAGGAAAGCCGCTTCCGA 400

R 401 CGCTATGGAACTCTCTTCTAAGCTTGTTATCAGTTTTTACAAAGGCAATT 450

||||||||||||||||||||||||||||||||||||||||||||||||||

S 401 CGCTATGGAACTCTCTTCTAAGCTTGTTATCAGTTTTTACAAAGGCAATT 450

R 451 TGGTTCAAATCTGTAGAAGCCAAGATAAGCATTCTAACAGATGTCAGTGG 500

||||||||||||||||||||||||||||||||||||||||||||||||||

S 451 TGGTTCAAATCTGTAGAAGCCAAGATAAGCATTCTAACAGATGTCAGTGG 500

R 501 CATCATCAAACCATCAAGGCTTACTCTCCTTCTTGGTCCCCCAGGCTGTG 550

||||||||||||||||||||||||||||||||||||||||||||||||||

S 501 CATCATCAAACCATCAAGGCTTACTCTCCTTCTTGGTCCCCCAGGCTGTG 550

R 551 GGAAAACCACCTTGTTACAAGCTCTTGCAGGAAAACAAGACAAATCTCTC 600

||||||||||||||||||||||||||||||||||||||||||||||||||

S 551 GGAAAACCACCTTGTTACAAGCTCTTGCAGGAAAACAAGACAAATCTCTC 600

R 601 AAGGTTTCAGGGGAGATTTCTTATAATGGTCACAAGCTAGATGAGTTTGT 650

||||||||||||||||||||||||||||||||||||||||||||||||||

S 601 AAGGTTTCAGGGGAGATTTCTTATAATGGTCACAAGCTAGATGAGTTTGT 650

R 651 CCCCCAGAAAACGTCAGCTTACATAAGCGAATACGACCTTCACATACCTG 700

||||||||||||||||||||||||||||||||||||||||||||||||||

S 651 CCCCCAGAAAACGTCAGCTTACATAAGCGAATACGACCTTCACATACCTG 700

R 701 AGTTGACTGTGAGGGAAACAATCGACTTTTCTGCCCGCTGTCAGGGTGTT 750

||||||||||||||||||||||||||||||||||||||||||||||||||

S 701 AGTTGACTGTGAGGGAAACAATCGACTTTTCTGCCCGCTGTCAGGGTGTT 750

R 751 CGGAGTAGAGCTGATATAATGATGGAGGTCAGCAGAAGAGAAAAAGAAGC 800

||||||||||||||||||||||||||||||||||||||||||||||||||

S 751 CGGAGTAGAGCTGATATAATGATGGAGGTCAGCAGAAGAGAAAAAGAAGC 800

R 801 AGGAATAGTCCCTGATCCAGATATTGACACCTACATGAAGGCAATATCAG 850

||||||||||||||||||||||||||||||||||||||||||||||||||

S 801 AGGAATAGTCCCTGATCCAGATATTGACACCTACATGAAGGCAATATCAG 850

R 851 TTCAAGGACAAAAGAGAAATCTCCAAACCGATTACATTTTGAAGATCCTC 900

||||||||||||||||||||||||||||||||||||||||||||||||||

S 851 TTCAAGGACAAAAGAGAAATCTCCAAACCGATTACATTTTGAAGATCCTC 900

R 901 GGACTGGATACCTGTAGTGACACAATGGTTGGTGATGCATTAAGTAGAGG 950

||||||||||||||||||||||||||||||||||||||||||||||||||

S 901 GGACTGGATACCTGTAGTGACACAATGGTTGGTGATGCATTAAGTAGAGG 950

R 951 CATTTCAGGTGGCCAGAAGAAGAGGCTGACAACAGGAGAGATGATTGTAG 1000

||||||||||||||||||||||||||||||||||||||||||||||||||

S 951 CATTTCAGGTGGCCAGAAGAAGAGGCTGACAACAGGAGAGATGATTGTAG 1000

R 1001 GTCCCACAAAAGCTCTATTTATGGATGAAATATCGACGGGATTAGACAGC 1050

||||||||||||||||||||||||||||||||||||||||||||||||||

S 1001 GTCCCACAAAAGCTCTATTTATGGATGAAATATCGACGGGATTAGACAGC 1050

R 1051 TCTACAACATTTCAGATAGTTACCTATCTCCAGCAGTTGGTGCACATCAC 1100

||||||||||||||||||||||||||||||||||||||||||||||||||

S 1051 TCTACAACATTTCAGATAGTTACCTATCTCCAGCAGTTGGTGCACATCAC 1100

R 1101 AGATGCAACTGCATTGGTATCACTTCTTCAACCAGCACCTGAGACCTTTG 1150

||||||||||||||||||||||||||||||||||||||||||||||||||

S 1101 AGATGCAACTGCATTGGTATCACTTCTTCAACCAGCACCTGAGACCTTTG 1150

R 1151 ATCTGTTTGATGATGTAATATTAATGGGAGAGGGGAAGGTAGTTTACCAT 1200

||||||||||||||||||||||||||||||||||||||||||||||||||

S 1151 ATCTGTTTGATGATGTAATATTAATGGGAGAGGGGAAGGTAGTTTACCAT 1200

R 1201 GGTCCTCGCAGCCACACACTTCAGTTTTTTGAAGATTGCGGTTTCAAGTG 1250

|||||||||||||||.||||||||||||||||||||||||||||||||||

S 1201 GGTCCTCGCAGCCACGCACTTCAGTTTTTTGAAGATTGCGGTTTCAAGTG 1250

R 1251 CCCATCAAGAAAGGGTGCTGCAGATTTCCTTCAGGAGGTAATCTCAAAGA 1300

||||||||||||||||||||||||||||||||||||||||||||||||||

S 1251 CCCATCAAGAAAGGGTGCTGCAGATTTCCTTCAGGAGGTAATCTCAAAGA 1300

R 1301 AGGATCAAGCACAATACTGGAAGCATGATAACATTCCCTACTACCATGTT 1350

|||||||||||||||||||||||||||||||||||||||||||.||||||

S 1301 AGGATCAAGCACAATACTGGAAGCATGATAACATTCCCTACTATCATGTT 1350

R 1351 TCAGTGGATCAGTTATCACAACTTTTCAGAGCAAGTTACTTAGGAAATAA 1400

||||||||||||||||||||||||||||||||||||||||||||||||||

S 1351 TCAGTGGATCAGTTATCACAACTTTTCAGAGCAAGTTACTTAGGAAATAA 1400

R 1401 GTTAGACGATGAGCTCTCAAAACCGTATGATAAATCGCAGTCCCATGACA 1450

||||||||||||||||||||||||||||||||||||||||||||||||||

S 1401 GTTAGACGATGAGCTCTCAAAACCGTATGATAAATCGCAGTCCCATGACA 1450

R 1451 ATGCCTTATCATTTACCACTTACTCTGTGAGCAAATGGGAGTTGTTCAAA 1500

||||||||||||||||||||||||||||||||||||||||||||||||||

S 1451 ATGCCTTATCATTTACCACTTACTCTGTGAGCAAATGGGAGTTGTTCAAA 1500

R 1501 GCTTGCATGGCCAGGGAGCTGCTTCTTATGAAACGAAATTCCTTTGTTTA 1550

||||||||||||||||||||||||||||||||||||||||||||||||||

S 1501 GCTTGCATGGCCAGGGAGCTGCTTCTTATGAAACGAAATTCCTTTGTTTA 1550

R 1551 TGTATTCAAAACAGTGCAG--------------GTTTATTGTCACTGA-- 1584

||||||||||||||||||| .|||||| .||.|||

S 1551 TGTATTCAAAACAGTGCAGCTTATCATCATTGCATTTATT-ACAATGACA 1599

R 1585 -------------------------------------------------- 1584

S 1600 GTATTTATAAGGACTCAGATGGCTGTGAATTTGACAAGCGCAAATTTTTT 1649

R 1585 -------------------------------------------------- 1584

S 1650 GTTGGGCGCATTGTTTTATACACTTGTTCGACATATGACAAATGGTGTTG 1699

R 1585 -------------------------------------------------- 1584

S 1700 CAGAGCTGTCCTTGACTGTTACTAGACTTCCAGTAGTTTACAAGCAAAGA 1749

R 1585 -------------------------------------------------- 1584

S 1750 GGATTCTATTTGTACCCAGCATGGGCGTATTCTATTCCGGCCTCTATGCT 1799

R 1585 -------------------------------------------------- 1584

S 1800 GAAGGTTCCATTTTCATTCATGGATTCAGTGCTTTGGACCGCCACAACTT 1849

R 1585 -------------------------------------------------- 1584

S 1850 ACTATGTTATTGGGTATAGCCCAGAAATAAAAAGGTTCTTCTGCCAGTTC 1899

R 1585 -------------------------------------------------- 1584

S 1900 CTTGTGCTATTTGCTCTGCATCAAGCATCAACATCCATGTGTCGTTTGGT 1949

R 1585 -------------------------------------------------- 1584

S 1950 TGCTGTAATTTTCCGAACTATGGTTGCTGCAACAACTTGTGGTACTTTTA 1999

R 1585 -------------------------------------------------- 1584

S 2000 TCTTAGTGGTAATGTTTTTATGTGGAGGCTTCATTTTGCCACGACCCTCT 2049

R 1585 -------------------------------------------------- 1584

S 2050 CTACCTCCGTGGTTGAGGTGGGTATTCTGGTGTTCTCCTATGACTTATGG 2099

R 1585 -------------------------------------------------- 1584

S 2100 GGAAATAGGTACAGCTCTAAATGAATTCCTTGCTCCTCGCTGGCAAAAGG 2149

R 1585 -------------------------------------------------- 1584

S 2150 TTTCAAAGGGAAACACAACCTTAGGGAATGAAGTTCTAACCAGTCATGGT 2199

R 1585 -------------------------------------------------- 1584

S 2200 CTGAACTTTGATGGCTCTTTCTATTGGATATCGGTAGGAGCATTATTCGG 2249

R 1585 -------------------------------------------------- 1584

S 2250 CTTCACAGTACTTTTCGATCTTGGATTTGCTTTAGCCTTAACTTACTTAA 2299

R 1585 -------------------------------------------------- 1584

S 2300 ATCCTCCAAAGATGTCTCGGGCTATTATTTCGGAAAAGAGGTTATCTCAA 2349

R 1585 -------------------------------------------------- 1584

S 2350 CTACAAGGGAAAGATGCTTGCAACACCAGTGCTCAGTCAGAGAATGTATC 2399

R 1585 -------------------------------------------------- 1584

S 2400 AACTCCAGCTGATCTTTACCAAAATGTGGGAGAAAAGTTAAAATTTGGGA 2449

R 1585 -------------------------------------------------- 1584

S 2450 AGATGGCCCTGGCTCTGCCATTTGAACCACTGACAATGTCATTTAAGGAT 2499

R 1585 -------------------------------------------------- 1584

S 2500 GTGCAGTATTATGTTGATACCCCTCCGGAAATGAGAGAGCATGGTTTCAA 2549

R 1585 -------------------------------------------------- 1584

S 2550 GCAGAAAAAGCTTCAGTTGCTTAAAGATATTACAGGAGCATTCAGACCTG 2599

R 1585 -------------------------------------------------- 1584

S 2600 GAATTCTTACAGCATTGATGGGTGTCAGTGGAGCTGGGAAAACAACTCTC 2649

R 1585 -------------------------------------------------- 1584

S 2650 ATGGATGTTCTTTCTGGAAGGAAAACAGGAGGTACTATTGAAGGAGATAT 2699

R 1585 -------------------------------------------------- 1584

S 2700 AAGAATAGGAGGGCACCCCAAGGTCCAGAAGACATTTGCAAGAATATCAG 2749

R 1585 -------------------------------------------------- 1584

S 2750 GTTACTGTGAGCAGACTGATATACATTCTCCACATATCACAGTAGGAGAA 2799

R 1585 -------------------------------------------------- 1584

S 2800 TCAGTTATGTACTCAGCTTGGTTGCGGTTGCCACCTGACACTGATCCAGA 2849

R 1585 -------------------------------------------------- 1584

S 2850 CACAAAATCTAGATTTGTGGAAGAAGTCATTGAAACGATTGAACTGGAGG 2899

R 1585 -------------------------------------------------- 1584

S 2900 ATATAAAAGATTCTTTAGTTGGGATTCCTGGACAAAGTGGCCTATCCACT 2949

R 1585 -------------------------------------------------- 1584

S 2950 GAGCAGCGTAAAAGGCTAACAATTGCAGTGGAGCTTGTTTCCAATCCATC 2999

R 1585 -------------------------------------------------- 1584

S 3000 CATAATATTTATGGATGAACCTACATCAGGTTTAGACGCCAGAGCAGCTG 3049

R 1585 -------------------------------------------------- 1584

S 3050 CAATTGTCATGCGCGCAGTGAAGAACGTCGTTGACACAGGAAGGACAACT 3099

R 1585 -------------------------------------------------- 1584

S 3100 GTTTGCACGATTCACCAACCAAGCATTGATATCTTTGAGTCTTTTGATGA 3149

R 1585 -------------------------------------------------- 1584

S 3150 GTTAATTTTGATGAAAACGGGAGGACAGATCATCTATTCTGGAATATTAG 3199

R 1585 -------------------------------------------------- 1584

S 3200 GACATCAATCGAGTAAACTAATCGAATATTTCGAGGGCATTCCTGGTGTA 3249

R 1585 -------------------------------------------------- 1584

S 3250 CCAAAAATCAAAGATAATTACAATCCAGCAACATGGATGTTAGAAGTTAC 3299

R 1585 -------------------------------------------------- 1584

S 3300 TTCCGCTTCGGTAGAGGAAGAACTTGGTTTAGATTTTGCCAGCATTTATA 3349

R 1585 -------------------------------------------------- 1584

S 3350 GAGAGTCTACTCAGTATAGGGACACAATTGAGCTGGTAAGACAGTTAAGC 3399

R 1585 -------------------------------------------------- 1584

S 3400 GTGCCAAAGCCGGGTTCAAAGGACTTGTATTTTCCCACTCATTTTCCTCA 3449

R 1585 -------------------------------------------------- 1584

S 3450 AAATAGCTGGGTGCAGTTTAAGGCATGCCTTTGGAAACAACACTTGTCCT 3499

R 1585 -------------------------------------------------- 1584

S 3500 ACTGGAGAAGTCCTGAATACAATCTAGCACGTTTCATGTTTATGATTGGT 3549

R 1585 -------------------------------------------------- 1584

S 3550 GCATCAGTGTTGTTTGGGATAATCTTTTGGCAGAAAGGGAAGGAAATAAA 3599

R 1585 -------------------------------------------------- 1584

S 3600 TAATGAGCAGGATTTGTTGAACATACTTGGGTCCATGTACATTGCCGTAA 3649

R 1585 -------------------------------------------------- 1584

S 3650 TATTCTTGGGCGTAACCAATTGCAACTTAGTTCTGCCTTATGTGGAAACT 3699

R 1585 -------------------------------------------------- 1584

S 3700 GAGCGCACTGTTTTGTACCGGGAACGATTTGCTGGGATGTACTCATCAAA 3749

R 1585 -------------------------------------------------- 1584

S 3750 GGCTTATTCGTTTGCGCAGGTGGCCGTGGAAATGCCATACACATTGTTGC 3799

R 1585 -------------------------------------------------- 1584

S 3800 AAGCAATTCTGTTTGTGATTATAACATACCCTACAATAGGGTATTATTGG 3849

R 1585 -------------------------------------------------- 1584

S 3850 TCAGCTACTAAGGTTTTTTGGTACTTTTATGCAACATTCTGGACATTTCT 3899

R 1585 -------------------------------------------------- 1584

S 3900 GTACTTTGTGTATCTTGGGATGCTGATTGCTTCTTTGAGCACAAACCTGG 3949

R 1585 -------------------------------------------------- 1584

S 3950 ATGTTGCTTCCATATTGGCAACTGCAGTTTACACCATATTGAATCTTTTC 3999

R 1585 -------------------------------------------------- 1584

S 4000 TCGGGCTTCCTCATGCCGGGACCGAAAATTCCTAAGTGGTGGGTTTGGTG 4049

R 1585 -------------------------------------------------- 1584

S 4050 CTATTGGATATGTCCTACATCATGGTCCCTAAATGGCCTCCTGACCTCAC 4099

R 1585 -------------------------------------------------- 1584

S 4100 AATATGGAGACATGAACAAAGAGATCTTGATTTTTGGGGAGCATAAAACA 4149

R 1585 -------------------------------------------------- 1584

S 4150 GTTGGTTCCTTCCTACAAGATTACTATGGTTTCCATCATGATGGTTTAGC 4199

R 1585 -------------------------------------------------- 1584

S 4200 CCTTGTGGCTATTGTTCTCATTGCTTTCCCAGTTGCTTATGCGTCTCTAT 4249

R 1585 ----------------------------------------- 1584

S 4250 TTGCCTATTGCATTGGGAAATTAAATTTTCAAAGAAGGTAG 4290

**Pa31 - Probable inactive serine/threonine-protein kinase fnkC - MATH**

Aligned_sequences: 2

1: **Par.chr1R_long.6.147**

2: **Par.chr1S_long.6.121_A**

Matrix: EBLOSUM62

Gap_penalty: 10.0

Extend_penalty: 0.5

Length: 319

Identity: 269/319 (84.3%)

Similarity: 286/319 (89.7%)

Gaps: 0/319 ( 0.0%)

Score: 1446.0

R 1 MNMTSLNFDEQDGILRTISDAPPTHYMIKIQSLSLLSVHSLEKYESGEFE 50

||||||||||||||||||||.|||||.||||||||||||||||||||.||

S 1 MNMTSLNFDEQDGILRTISDVPPTHYTIKIQSLSLLSVHSLEKYESGVFE 50

R 51 AGGYKWKLVFYPNGNKSRNVKEHISLYLVLAGANAPKTCWEVHAAFRLFL 100

|||||||||||||||||.|.||||||||||||||.|:|||||||||||||

S 51 AGGYKWKLVFYPNGNKSSNGKEHISLYLVLAGANGPQTCWEVHAAFRLFL 100

R 101 LDQNIGKYFAFQEQNERCFHGMKLDWGFDKCLSLKAFTDASNGFLVEDTC 150

||||.|||.|.||:|||.|||||||||||:.||||||||.|||||:||.|

S 101 LDQNTGKYLALQEKNERRFHGMKLDWGFDQFLSLKAFTDTSNGFLMEDAC 150

R 151 VFGAEVFVRKERSTCKGECLSMIKGAIMYKHVWKIDNFSKLNAESYDSQT 200

|||||||||||:||||||||||||.|:|||||||||||||||||||||.|

S 151 VFGAEVFVRKEKSTCKGECLSMIKDAVMYKHVWKIDNFSKLNAESYDSPT 200

R 201 FIAGDQKWKIKLYPKGRDGAASGHLSLYLALADPTSLPPTSKIYAEFTLR 250

||||:|||||:|||||||.....||||||||||||||||||||||::|||

S 201 FIAGNQKWKIRLYPKGRDSGTGSHLSLYLALADPTSLPPTSKIYAQYTLR 250

R 251 LINQQNSSLHYAYSKVNWWFSASSPMRGWGRFITVGWFYVNQANYRYLVN 300

:|||.||...|.||||.||||||||.|||..|||:|:|.:.|:|:.|||.

S 251 IINQLNSPYPYEYSKVTWWFSASSPSRGWPSFITIGYFNIAQSNWGYLVK 300

R 301 DSCTVEAEVTVHGTASALE 319

||||||||||||||||||:

S 301 DSCTVEAEVTVHGTASALD 319

Aligned_sequences: 2

1: **Par.chr1R_long.6.147**

2: **Par.chr1S_long.6.121_A**

Matrix: EDNAFULL

Gap_penalty: 10.0

Extend_penalty: 0.5

Length: 961

Identity: 880/961 (91.6%)

Similarity: 880/961 (91.6%)

Gaps: 2/961 ( 0.2%)

Score: 4064.0

Green color: START CODON

Red color: STOP CODON

R 1 ATGAACATGACTAGTCTCAACTTCGATGAACAAGACGGGATTTTGAGAAC 50

|||||||||||||||||.||||||||||||||||||||||||||||||||

S 1 ATGAACATGACTAGTCTTAACTTCGATGAACAAGACGGGATTTTGAGAAC 50

R 51 AATTTCTGATGCGCCGCCAACTCATTACATGATCAAAATACAGTCGCTTT 100

|||||||||||.|||||||||||||||||.||||||||||||||||||||

S 51 AATTTCTGATGTGCCGCCAACTCATTACACGATCAAAATACAGTCGCTTT 100

R 101 CATTGCTGTCTGTCCATTCACTGGAGAAATATGAATCAGGGGAGTTTGAA 150

|||||||.||.||.||||||||||||||||||||||||||||.|||||||

S 101 CATTGCTATCCGTACATTCACTGGAGAAATATGAATCAGGGGTGTTTGAA 150

R 151 GCTGGAGGATACAAATGGAAACTGGTTTTCTATCCAAATGGAAACAAGAG 200

|||||||||||||||||||||.||||||||||||||||||||||||||||

S 151 GCTGGAGGATACAAATGGAAATTGGTTTTCTATCCAAATGGAAACAAGAG 200

R 201 CAGGAATGTGAAAGAGCACATCTCTCTCTACTTAGTATTGGCCGGAGCAA 250

|||.||||.|||||||||||||||||||||||||||||||||||||||||

S 201 CAGCAATGGGAAAGAGCACATCTCTCTCTACTTAGTATTGGCCGGAGCAA 250

R 251 ATGCCCCCAAGACTTGTTGGGAGGTGCATGCTGCTTTCAGGTTGTTTTTG 300

|||.||||.|||||||||||||.||||||||.||||||||||||||||||

S 251 ATGGCCCCCAGACTTGTTGGGAAGTGCATGCCGCTTTCAGGTTGTTTTTG 300

R 301 CTTGATCAGAATATTGGCAAGTACTTTGCTTTTCAAGAACAAAATGAAAG 350

|||||||||||||.||||||||||||.||..||||||||.||||||||||

S 301 CTTGATCAGAATACTGGCAAGTACTTGGCACTTCAAGAAAAAAATGAAAG 350

R 351 GTGCTTCCATGGGATGAAGTTAGACTGGGGATTTGATAAATGCCTCTCCC 400

|.|||||||||||||||||.|.|||||||||||||||.|||.||||||.|

S 351 GCGCTTCCATGGGATGAAGCTTGACTGGGGATTTGATCAATTCCTCTCTC 400

R 401 TCAAAGCTTTTACTGACGCTTCCAATGGATTTCTCGTGGAAGACACCTGT 450

|||||||||||||||||.|||||||||||||||||.|||||||..||||.

S 401 TCAAAGCTTTTACTGACACTTCCAATGGATTTCTCATGGAAGATGCCTGC 450

R 451 GTGTTTGGAGCGGAGGTCTTTGTTCGTAAAGAAAGAAGCACATGCAAAGG 500

||||||||||||||||||||.|||||||||||||.|||||||||||||||

S 451 GTGTTTGGAGCGGAGGTCTTCGTTCGTAAAGAAAAAAGCACATGCAAAGG 500

R 501 AGAGTGTCTATCAATGATCAAGGGTGCCATTATGTACAAGCATGTTTGGA 550

|||.|||||||||||||||||||.||||.|||||||||||||||||||||

S 501 AGAATGTCTATCAATGATCAAGGATGCCGTTATGTACAAGCATGTTTGGA 550

R 551 AAATTGACAACTTCTCAAAGTTAAACGCGGAGAGCTATGACTCACAAACA 600

||||||||||||||||.||||||||||||||||||||||||||||.||||

S 551 AAATTGACAACTTCTCGAAGTTAAACGCGGAGAGCTATGACTCACCAACA 600

R 601 TTCATTGCTGGAGACCAAAAATGGAAGATAAAACTGTATCCGAAGGGAAG 650

||||||||||||.||||.||||||||||||..||||||||||||||||||

S 601 TTCATTGCTGGAAACCAGAAATGGAAGATACGACTGTATCCGAAGGGAAG 650

R 651 AGACGGTGCGGCGAGTGGCCATCTCTCTCTTTATTTGGCATTAGCTGATC 700

||||.|||.|.|..||.|||||||||||||||||||||||||.|||||||

S 651 AGACAGTGGGACCGGTAGCCATCTCTCTCTTTATTTGGCATTGGCTGATC 700

R 701 CGACATCTCTGCCTCCTACCTCTAAAATATATGCAGAGTTTACCCTGCGC 750

|||||||||||||||||||||||||||||||||||.|||.||||||||||

S 701 CGACATCTCTGCCTCCTACCTCTAAAATATATGCACAGTATACCCTGCGC 750

R 751 CTCATAAATCAACAGAACAGCAGCCT-TCACTATGCATATAGTAAAGTTA 799

.||||||||||||.||||||| .||| ||.|||||.||||||||||||||

S 751 ATCATAAATCAACTGAACAGC-CCCTATCCCTATGAATATAGTAAAGTTA 799

R 800 ATTGGTGGTTCAGTGCCTCCAGTCCCATGCGGGGCTGGGGTAGATTCATT 849

.||||||||||||||||||||||||||..|||||||||...||.||||||

S 800 CTTGGTGGTTCAGTGCCTCCAGTCCCAGCCGGGGCTGGCCGAGTTTCATT 849

R 850 ACAGTCGGATGGTTCTATGTGAATCAGGCGAACTACCGGTACTTGGTGAA 899

|||.||||||..|||.||.|...||||.||||||...|||||||||||||

S 850 ACAATCGGATATTTCAATATTGCTCAGTCGAACTGGGGGTACTTGGTGAA 899

R 900 CGATAGTTGTACCGTGGAGGCCGAGGTCACTGTGCATGGAACTGCTAGTG 949

.||||||||.|||||||||||.||||||||||||||||||||||||||||

S 900 GGATAGTTGCACCGTGGAGGCAGAGGTCACTGTGCATGGAACTGCTAGTG 949

R 950 CGCTAGAGTAA 960

|.|||||.|||

S 950 CTCTAGACTAA 960

**Pa32 - BTB/POZ and MATH domain-containing protein 3-like**

Aligned_sequences: 2

1: **Par.chr1R_long.6.150_A**

2: **Par.chr1S_long.6.121_B**

Matrix: EBLOSUM62

Gap_penalty: 10.0

Extend_penalty: 0.5

Length: 318

Identity: 38/318 (11.9%)

Similarity: 39/318 (12.3%)

Gaps: 276/318 (86.8%)

Score: 185.5

R 1 MSMNNLNFDDQYGILRTFSDSMPTHYTFKIQSFSLMS------FTGEI-- 42

||||||||||||||||||||||||||||||||||||| :..|.

S 1 MSMNNLNFDDQYGILRTFSDSMPTHYTFKIQSFSLMSKHSLERYESEDFE 50

R 43 -------------------------------------------------- 42

S 51 AGGYKWKLAFYPNGNKSKNVKEHISLYLVLAGANGPQTCWEVYAAFRLFL 100

R 43 -------------------------------------------------- 42

S 101 LDQNNGKYLALQEQKERCFHGIKLDWGFDQFLSQKDFTDASNGFLVDDAC 150

R 43 -------------------------------------------------- 42

S 151 VFGAEVFVRKERSTCKGECLSMIKDAVMYKHVWKIENLSKLDEESYDSET 200

R 43 -------------------------------------------------- 42

S 201 FIAGDQKWKIEFYPKGRDDGKDSHLSIDLALADPTSLSPTSKLYAQFTLR 250

R 43 -------------------------------------------------- 42

S 251 LVDPVYSHRHFEYGTKATWWFSASSPKRGWPKFITLGIFGDESVGYLEND 300

R 43 ------------------ 42

S 301 STILEAEVTVLGTAIALD 318

Aligned_sequences: 2

1: **Par.chr1R_long.6.150_A**

2: **Par.chr1S_long.6.121_B**

Matrix: EDNAFULL

Gap_penalty: 10.0

Extend_penalty: 0.5

Length: 964

Identity: 922/964 (95.6%)

Similarity: 922/964 (95.6%)

Gaps: 12/964 ( 1.2%)

Score: 4436.5

Green color: START CODON

Red color: STOP CODON

R 1 ATGAGCATGAATAACCTTAACTTCGATGACCAATACGGGATTTTGAGAAC 50

||||||||||||||||||||||||||||||||||||||||||||||||||

S 1 ATGAGCATGAATAACCTTAACTTCGATGACCAATACGGGATTTTGAGAAC 50

R 51 ATTTTCTGATTCCATGCCAACTCATTACACGTTCAAAATACAGTCATTTT 100

||||||||||||.||||||||||||||||||||||||||||||||.||||

S 51 ATTTTCTGATTCAATGCCAACTCATTACACGTTCAAAATACAGTCGTTTT 100

R 101 CATTGATGT-----CATTCACTGGAGAGATATGAATCGGAGGACTTTGAA 145

||||||||| ||||||||||||||||||||||||||||||||||||

S 101 CATTGATGTCCAAACATTCACTGGAGAGATATGAATCGGAGGACTTTGAA 150

R 146 GCTGGAGGATACAAATAGGAAACTGGCTTTCTATCCAAATGGAAACAAGA 195

|||||||||||||||| |||||||||||||.|||||||||||||||||||

S 151 GCTGGAGGATACAAAT-GGAAACTGGCTTTTTATCCAAATGGAAACAAGA 199

R 196 GCAAGAATGTGAAAGAGCACATCTCTCTCTACTTAGTATTGGCTGGAGCA 245

||||||||||||||||||||||||||||.|||||||||||||||||||||

S 200 GCAAGAATGTGAAAGAGCACATCTCTCTTTACTTAGTATTGGCTGGAGCA 249

R 246 AATGGCCCCCAGACCTGTTGGGAAGTGTATGCTGCTTTCAGGTTGTTTTT 295

||||||||||||||||||||||||||||||||||||||||||||||||.|

S 250 AATGGCCCCCAGACCTGTTGGGAAGTGTATGCTGCTTTCAGGTTGTTTCT 299

R 296 ACTTGATCAGAACAATGGCAAGTACTTGGCTCTTCGAAGAACAAAAGGAA 345

||||||||||||||||||||||||||||||||||| ||||||||||||||

S 300 ACTTGATCAGAACAATGGCAAGTACTTGGCTCTTC-AAGAACAAAAGGAA 348

R 346 AGGTGCTTCCATGGGATTAAGCTTGACTGGGGATTTGATCAATTTCTCTC 395

||||||||||||||||||||||||||||||||||||||||||||||||||

S 349 AGGTGCTTCCATGGGATTAAGCTTGACTGGGGATTTGATCAATTTCTCTC 398

R 396 TCAAAAAGATTTCACTGATGCTTCCAATGGATTTCTTGTGGATGACGCCT 445

||||||||||||||||||||||||||||||||||||||||||||||||||

S 399 TCAAAAAGATTTCACTGATGCTTCCAATGGATTTCTTGTGGATGACGCCT 448

R 446 GTGTGTTTGGAGCAGAGGTCTTTGTTCGTAAAGAAAGAAGCACATGCAAA 495

||||||||||||||||||||||||||||.|||||||||||||||||||||

S 449 GTGTGTTTGGAGCAGAGGTCTTTGTTCGGAAAGAAAGAAGCACATGCAAA 498

R 496 GGAGAGTGTCTATCAATGATCAAGGATGCCGTTATGTACAAGCATGTTTG 545

||||||||.|||||||||||||||||||||||||||||||||||||||||

S 499 GGAGAGTGCCTATCAATGATCAAGGATGCCGTTATGTACAAGCATGTTTG 548

R 546 GAAAATTGAAAACTTGTCAAAGTTAGACAAGGAGAGCTACGACTCAGAAA 595

||||||||||||||||||.||||||||..|||||||||||||||||||||

S 549 GAAAATTGAAAACTTGTCGAAGTTAGATGAGGAGAGCTACGACTCAGAAA 598

R 596 CATTCATCGCTGGAGACCAAAAATAGGAAGATAGAGTTCTATCCCGAGGG 645

|||||||||||||||||||.|||| ||||.|||||||||||||||.||||

S 599 CATTCATCGCTGGAGACCAGAAAT-GGAAAATAGAGTTCTATCCCAAGGG 647

R 646 AAGAGACGATGGGAAGGGTAGCCATCTTTCTATTGATTTGGCATTGGCTG 695

|||||||||||||||||.||||||||||||||||||||||||||||||||

S 648 AAGAGACGATGGGAAGGATAGCCATCTTTCTATTGATTTGGCATTGGCTG 697

R 696 ATCCCACATCTCTGTCTCCTACCTCTAAATTGTATGCACAGTTTACCCTT 745

|||||||||||||||||||||||||||||||.||||||||||||||||||

S 698 ATCCCACATCTCTGTCTCCTACCTCTAAATTATATGCACAGTTTACCCTT 747

R 746 CGCCTCGTAGATCCAGTGTACAGCTCCCGTCACTTTGAATATGGTGCTAA 795

||||||||||||||||||||||||..|||||||||||||||||||.||||

S 748 CGCCTCGTAGATCCAGTGTACAGCCACCGTCACTTTGAATATGGTACTAA 797

R 796 TTACAGCTACTTGGTGGTTCAGTGCCTCCAGTCCGAAGAGGGGCTGGCCG 845

||||||||||||||||||||||||||||||||||.|||||||||||

S 798 ----AGCTACTTGGTGGTTCAGTGCCTCCAGTCCGAAGCGGGGCTGGCCG 843

R 846 AAATTCATTACACTGGGACATTTCAGTGATAAGACCTTGGGGTATTTGGA 895

||||||||||||||||||..||||.|||||.|||.|.|||||||||||||

S 844 AAATTCATTACACTGGGAATTTTCGGTGATGAGAGCGTGGGGTATTTGGA 893

R 896 GAATGATAGTACCATTGTGGAGGCAGAGGTCACTGTGCTTGGAACTGCTA 945

|||||||||||||||..|.|||||||||||||||||||||||||||||||

S 894 GAATGATAGTACCATCCTAGAGGCAGAGGTCACTGTGCTTGGAACTGCTA 943

R 946 GTGCACTAGACTAA 959

.|||.|||||||||

S 944 TTGCGCTAGACTAA 957

**Pa33 - BTB/POZ and MATH domain-containing protein 3-like**

Aligned_sequences: 2

1: **Par.chr1R_long.6.150_B**

2: **Par.chr1S_long.6.121_C**

Matrix: EBLOSUM62

Gap_penalty: 10.0

Extend_penalty: 0.5

Length: 313

Identity: 293/313 (93.6%)

Similarity: 294/313 (93.9%)

Gaps: 11/313 ( 3.5%)

Score: 1494.5

R 1 MMTSLNFDAQDGILRSFSDAPPTHYTVKIQSLSLLAKNSLEKYESGDFEA 50

||||||||||||||||||||||||||||||||||||||||||||||||||

S 1 MMTSLNFDAQDGILRSFSDAPPTHYTVKIQSLSLLAKNSLEKYESGDFEA 50

R 51 GG---KLVFYPNGNKSRNVKDHISLYLVMSGANATHISREVYAVFRLFLL 97

|| ||||||||||||||||||||||||||||||.||||||||||||||

S 51 GGYKWKLVFYPNGNKSRNVKDHISLYLVMSGANATQISREVYAVFRLFLL 100

R 98 DQNKGNYLVLQEQNERRFHGMKLNWGFDQFLSQKVFTEASNGFLLDDTSV 147

|||||||||||||||||||||||:||||||||||.|||||||||||||||

S 101 DQNKGNYLVLQEQNERRFHGMKLDWGFDQFLSQKAFTEASNGFLLDDTSV 150

R 148 FGAEIFVCKERSTCKGEYLSMVKDAVMYKHVWKIDNFSKLDAEFYDSKT- 196

|||||||||||||||||.|||||||||||||||||||||||||||||||

S 151 FGAEIFVCKERSTCKGECLSMVKDAVMYKHVWKIDNFSKLDAEFYDSKTF 200

R 197 -------KIQLYPKGKGNGIGTHLSLYLALADPKSLPPGSKIYADITLRI 239

|||||||||||||||||||||||||.||||||||||||.||||

S 201 ISGDQKWKIQLYPKGKGNGIGTHLSLYLALADTKSLPPGSKIYADFTLRI 250

R 240 LDQVNARHQFGKGNFWFSASNPEWGWWRFITLGFLSQAGMGFLSKDTCIV 289

||||||||||||.||||||||||.||.|||||||||||||||||||||||

S 251 LDQVNARHQFGKVNFWFSASNPERGWLRFITLGFLSQAGMGFLSKDTCIV 300

R 290 EAEVTVHGISNAL 302

|||||||||||||

S 301 EAEVTVHGISNAL 313

Aligned_sequences: 2

1: **Par.chr1R_long.6.150_B**

2: **Par.chr1S_long.6.121_C**

Matrix: EDNAFULL

Gap_penalty: 10.0

Extend_penalty: 0.5

Length: 942

Identity: 898/942 (95.3%)

Similarity: 898/942 (95.3%)

Gaps: 33/942 ( 3.5%)

Score: 4410.5

Green color: START CODON

Red color: STOP CODON

R 1 ATGATGACTAGTCTTAACTTCGATGCCCAAGATGGGATTTTGAGAAGCTT 50

||||||||||||||||||||||||||||||||||||||||||||||||||

S 1 ATGATGACTAGTCTTAACTTCGATGCCCAAGATGGGATTTTGAGAAGCTT 50

R 51 TTCGGATGCACCACCGACTCATTACACAGTAAAAATACAGTCCCTTTCAT 100

||||||||||||||||||||||||||||||||||||||||||||||||||

S 51 TTCGGATGCACCACCGACTCATTACACAGTAAAAATACAGTCCCTTTCAT 100

R 101 TGCTGGCCAAAAATTCTTTGGAGAAATATGAATCAGGGGACTTTGAAGCT 150

||||||||||||||||||||||||||||||||||||||||||||||||||

S 101 TGCTGGCCAAAAATTCTTTGGAGAAATATGAATCAGGGGACTTTGAAGCT 150

R 151 GGAG---------GGAAACTGGTTTTCTATCCAAATGGAAACAAGAGCAG 191

|||| |||||||||||||||||||||||||||||||||||||

S 151 GGAGGGTACAAATGGAAACTGGTTTTCTATCCAAATGGAAACAAGAGCAG 200

R 192 GAATGTGAAAGATCACATCTCTCTCTACTTGGTAATGTCTGGTGCAAATG 241

||||||||||||||||||||||||||||||||||||||||||||||||||

S 201 GAATGTGAAAGATCACATCTCTCTCTACTTGGTAATGTCTGGTGCAAATG 250

R 242 CTACCCATATTTCTCGGGAAGTGTATGCTGTTTTCAGGTTGTTTTTACTT 291

|||||||.||||||||||||||||||||||||||||||||||||||||||

S 251 CTACCCAGATTTCTCGGGAAGTGTATGCTGTTTTCAGGTTGTTTTTACTT 300

R 292 GATCAGAATAAGGGCAACTACTTGGTTCTTCAAGAACAAAATGAAAGGCG 341

||||||||||||||||||||||||||||||||||||||||||||||||||

S 301 GATCAGAATAAGGGCAACTACTTGGTTCTTCAAGAACAAAATGAAAGGCG 350

R 342 CTTCCATGGGATGAAGCTCAATTGGGGATTTGATCAGTTCCTCTCTCAGA 391

||||||.||||||||||||.||||||||||||||||||||||||||||||

S 351 CTTCCACGGGATGAAGCTCGATTGGGGATTTGATCAGTTCCTCTCTCAGA 400

R 392 AAGTTTTTACTGAAGCTTCCAATGGATTTCTCTTAGATGACACCAGTGTG 441

|||.||||||||||||||||||||||||||||||||||||||||||||||

S 401 AAGCTTTTACTGAAGCTTCCAATGGATTTCTCTTAGATGACACCAGTGTG 450

R 442 TTTGGAGCAGAGATCTTTGTTTGTAAAGAGAGAAGTACATGCAAAGGAGA 491

||||||||||||||||||||||||||||||||||||||||||||||||||

S 451 TTTGGAGCAGAGATCTTTGTTTGTAAAGAGAGAAGTACATGCAAAGGAGA 500

R 492 GTATCTATCAATGGTAAAAGATGCCGTGATGTACAAGCATGTTTGGAAAA 541

||.|||||||||||||||||||||||||||||||||||||||||||||||

S 501 GTGTCTATCAATGGTAAAAGATGCCGTGATGTACAAGCATGTTTGGAAAA 550

R 542 TTGACAACTTCTCGAAGTTAGATGCGGAATTCTATGACTCAAAAAC---- 587

||||||||||||||||||||||||||||||||||||||||||||||

S 551 TTGACAACTTCTCGAAGTTAGATGCGGAATTCTATGACTCAAAAACGTTC 600

R 588 --------------------GAAGATTCAACTCTATCCCAAGGGAAAAGG 617

||||||||||||||||||||||||||||||

S 601 ATTTCTGGAGACCAGAAATGGAAGATTCAACTCTATCCCAAGGGAAAAGG 650

R 618 CAATGGAATTGGTACTCATCTTTCTCTTTATTTGGCATTGGCTGATCCAA 667

||||||||||||||||||||||||||||||||||||||||||||||.|.|

S 651 CAATGGAATTGGTACTCATCTTTCTCTTTATTTGGCATTGGCTGATACGA 700

R 668 AATCTCTTCCTCCAGGCTCTAAAATCTATGCAGATATTACGCTACGGATC 717

|||||||||||||||||||||||||||||||||||.||||||||||||||

S 701 AATCTCTTCCTCCAGGCTCTAAAATCTATGCAGATTTTACGCTACGGATC 750

R 718 CTGGACCAAGTGAATGCAAGGCATCAGTTTGGTAAAGGCAACTTCTGGTT 767

|||||||||||||||||||||||||||||||||||||.||||||||||||

S 751 CTGGACCAAGTGAATGCAAGGCATCAGTTTGGTAAAGTCAACTTCTGGTT 800

R 768 CAGCGCCTCAAATCCGGAGTGGGGTTGGTGGAGATTCATAACGCTGGGAT 817

|||||||||||||||||||.|||||||||.||||||||||||||||||||

S 801 CAGCGCCTCAAATCCGGAGCGGGGTTGGTTGAGATTCATAACGCTGGGAT 850

R 818 TTCTCAGCCAAGCAGGCATGGGGTTTTTGTCGAAGGATACTTGCATCGTG 867

||||||||||||||||||||||||||||||||||||||||||||||||||

S 851 TTCTCAGCCAAGCAGGCATGGGGTTTTTGTCGAAGGATACTTGCATCGTG 900

R 868 GAAGCAGAGGTCACTGTCCATGGAATTTCTAATGCCCTGTAG 909

||||||||||||||||||||||||||||||||||||||||||

S 901 GAAGCAGAGGTCACTGTCCATGGAATTTCTAATGCCCTGTAG 942

**Pa34 - probable inactive serine/threonine-protein kinase fnkC – MATH**

Aligned_sequences: 2

1: **Par.chr1R_long.6.135**

2: **Par.chr1S_long.6.21**

Matrix: EBLOSUM62

Gap_penalty: 10.0

Extend_penalty: 0.5

Length: 319

Identity: 51/319 (16.0%)

Similarity: 52/319 (16.3%)

Gaps: 267/319 (83.7%)

Score: 270.0

R 1 MSLIFDQDGLSRSLSNSPPTHYTLTIESFSMLTENSVDTYESGEFDAGGY 50

||||||:|||||||||||||||||||||||||||||||||||||||||||

S 1 MSLIFDRDGLSRSLSNSPPTHYTLTIESFSMLTENSVDTYESGEFDAGGY 50

R 51 KWKLVVYPNGNTKKNVEDHISVYLKMAEANSLQTGWEVSVDFRLFLLDQN 100

||

S 51 KW------------------------------------------------ 52

R 101 KGIYLVLQDANMNKMCLHGAMLQVGFDRVIPLNAFSVASNGYLIDDTCVF 150

S 53 -------------------------------------------------- 52

R 151 GAEVFVCKERRAGKAECLSRIKKAFMNKHCWKIESFSTLLFQCLQSELFT 200

S 53 -------------------------------------------------- 52

R 201 AGGQKWKIELYPKGDGDGENTHVSVYLSLLANPEKLSPGSQLLTECTVRI 250

S 53 -------------------------------------------------- 52

R 251 VDQLNGKDKSRELNHAWFSASSSSWGWPCFIKLDSFKMLDNGYLVKNTCL 300

S 53 -------------------------------------------------- 52

R 301 VEAEVTVHGIAKALEPTDD 319

S 53 ------------------- 52

Aligned_sequences: 2

1: **Par.chr1R_long.6.135**

2: **Par.chr1S_long.6.21**

Matrix: EDNAFULL

Gap_penalty: 10.0

Extend_penalty: 0.5

Length: 965

Identity: 949/965 (98.3%)

Similarity: 949/965 (98.3%)

Gaps: 5/965 ( 0.5%)

Score: 4660.5

Green color: START CODON

Red color: STOP CODON

R 1 ATGAGCCTTATATTTGACCAAGATGGTCTTTCGAGATCACTTTCAAATTC 50

|||||||||||||||||||.||||||||||||||||||||||||||||||

S 1 ATGAGCCTTATATTTGACCGAGATGGTCTTTCGAGATCACTTTCAAATTC 50

R 51 ACCACCAACTCATTACACTCTAACAATAGAGTCATTTTCGATGCTAACTG 100

||||||||||||||||||||||||||||||||||||||||||||||||||

S 51 ACCACCAACTCATTACACTCTAACAATAGAGTCATTTTCGATGCTAACTG 100

R 101 AAAATTCAGTGGATACATATGAGTCTGGGGAGTTTGATGCTGGAGGATAC 150

||||||||||||||||||||||||||||||||||||||||||||||||||

S 101 AAAATTCAGTGGATACATATGAGTCTGGGGAGTTTGATGCTGGAGGATAC 150

R 151 AAATGG--AAACTGGTGGTGTACCCAAATGGAAACACGAAGAAGAATGTA 198

|||||| ||||||||||||||||||||||||||||||||||||||||||

S 151 AAATGGTAAAACTGGTGGTGTACCCAAATGGAAACACGAAGAAGAATGTA 200

R 199 GAAGACCACATCTCTGTCTACTTAAAAATGGCAGAAGCAAATTCACTTCA 248

||||||.|||||||||||||||||||||||||||||||||||||||||||

S 201 GAAGACTACATCTCTGTCTACTTAAAAATGGCAGAAGCAAATTCACTTCA 250

R 249 GACTGGTTGGGAAGTATCTGTTGATTTTAGATTGTTTTTGCTTGATCAGA 298

||||||||||||||||||||||||||||||||||||||||||||||||||

S 251 GACTGGTTGGGAAGTATCTGTTGATTTTAGATTGTTTTTGCTTGATCAGA 300

R 299 ATAAGGGAATCTACTTGGTTCTTCA-GGATGCTAATATGAACAAGATGTG 347

||||||||||||||||||||||||| ||||||||||||||||||||||||

S 301 ATAAGGGAATCTACTTGGTTCTTCAGGGATGCTAATATGAACAAGATGTG 350

R 348 CTTGCACGGGGCGATGCTCCAAGTGGGTTTTGATAGAGTTATCCCTCTGA 397

||||||||||||||||||||||||||||||||||||||||||||||||||

S 351 CTTGCACGGGGCGATGCTCCAAGTGGGTTTTGATAGAGTTATCCCTCTGA 400

R 398 ATGCATTTTCTGTTGCTTCCAATGGCTATCTCATTGATGATACCTGTGTT 447

||||||||||||||||||||||||||||||||||||||||||||||||||

S 401 ATGCATTTTCTGTTGCTTCCAATGGCTATCTCATTGATGATACCTGTGTT 450

R 448 TTTGGAGCCGAGGTCTTCGTTTGCAAAGAAAGAAGAGCGGGCAAGGCAGA 497

||||||||||||||||||||||||||||||||||||||||||||||||||

S 451 TTTGGAGCCGAGGTCTTCGTTTGCAAAGAAAGAAGAGCGGGCAAGGCAGA 500

R 498 GTGTCTGTCAAGGATCAAAAAGGCTTTTATGAACAAGCATTGTTGGAAGA 547

||||||||||||||||||||||||||||||||||||||||||||||||||

S 501 GTGTCTGTCAAGGATCAAAAAGGCTTTTATGAACAAGCATTGTTGGAAGA 550

R 548 TTGAGAGTTTTTCAACGTTATTATTCCAATGCTTGCAATCAGAACTATTC 597

||||||||||||||||||||..||.|||||||||||||||||||||||||

S 551 TTGAGAGTTTTTCAACGTTAAAATCCCAATGCTTGCAATCAGAACTATTC 600

R 598 ACTGCAGGAGGGCAGAAATGG-AAGATAGAGCTCTATCCCAAGGGAGATG 646

||||||||||||||||||||| ||||||||||||||||||||||||||||

S 601 ACTGCAGGAGGGCAGAAATGGTAAGATAGAGCTCTATCCCAAGGGAGATG 650

R 647 GCGATGGAGAGAATACTCATGTTTCTGTTTACTTAAGTTTATTAGCTAAT 696

.|||||||||||||||||||||||||||||||||||||||||||||||||

S 651 ACGATGGAGAGAATACTCATGTTTCTGTTTACTTAAGTTTATTAGCTAAT 700

R 697 CCAGAAAAGCTATCTCCTGGTTCCCAATTACTTACAGAGTGTACTGTGCG 746

||||||||||||||||||||.|||||||||||||||||||||||||||||

S 701 CCAGAAAAGCTATCTCCTGGCTCCCAATTACTTACAGAGTGTACTGTGCG 750

R 747 CATCGTAGATCAACTGAACGGCAAGGATAAGAGTCGGGAAT-TGAATCAT 795

||||||||||||||||.|||||||||||||||||||||||| |||||.||

S 751 CATCGTAGATCAACTGGACGGCAAGGATAAGAGTCGGGAATGTGAATTAT 800

R 796 GCATGGTTCAGTGCCTCGAGTTCGTCTTGGGGTTGGCCTTGTTTCATTAA 845

||||||||||||||||||||||||.|||||||||||||||||||||||||

S 801 GCATGGTTCAGTGCCTCGAGTTCGACTTGGGGTTGGCCTTGTTTCATTAA 850

R 846 ATTGGACAGTTTCAAAATGTTAGACAACGGTTATTTGGTGAAGAATACTT 895

||||||||||||||||||||||||||||||||||||||||||||||||||

S 851 ATTGGACAGTTTCAAAATGTTAGACAACGGTTATTTGGTGAAGAATACTT 900

R 896 GCTTGGTCGAGGCTGAGGTCACTGTCCATGGAATTGCCAAAGCATTAGAG 945

|||||||||||||.||||||||||||||||||||||||||||||||||||

S 901 GCTTGGTCGAGGCCGAGGTCACTGTCCATGGAATTGCCAAAGCATTAGAG 950

R 946 CCAACTGACGATTAA 960

|||||||||||||||

S 951 CCAACTGACGATTAA 965

**Pa38 - Protein GFS12**

Aligned_sequences: 2

1: **Par.chr1R_long.6.117**

2: **Par.chr1S_long.6.40**

Matrix: EBLOSUM62

Gap_penalty: 10.0

Extend_penalty: 0.5

Length: 1667

Identity: 1621/1667 (97.2%)

Similarity: 1622/1667 (97.3%)

Gaps: 44/1667 ( 2.6%)

Score: 8518.5

R 1 MAHEMCFDCLHRRIQSDFSEKLVLVYGLSDSAFPFGSTAVVQLCNSSGET 50

||||||||||||||||||||||||||||||||||||||||||||||||||

S 1 MAHEMCFDCLHRRIQSDFSEKLVLVYGLSDSAFPFGSTAVVQLCNSSGET 50

R 51 ASAPQFLLSYLPSHEQDCLTKYVNEYIGDNAEGCSDSVIANTITSSEVTR 100

||||||||||||||||||||||||||||||||||||||||||||||||||

S 51 ASAPQFLLSYLPSHEQDCLTKYVNEYIGDNAEGCSDSVIANTITSSEVTR 100

R 101 DEEEVSSDVNNDQKPKLDSLLNMSPCLSNGGTKTFLQSSTCKHSSRFSCS 150

||||||||||||||||||||||||||||||||||||||||||||||||||

S 101 DEEEVSSDVNNDQKPKLDSLLNMSPCLSNGGTKTFLQSSTCKHSSRFSCS 150

R 151 RVISGLAPITHVGICSDSIFEELASEFLSRSLEDNILNSLSLLIEGKASG 200

||||||||||||||||||||||||||||||||||:|||||||||||||||

S 151 RVISGLAPITHVGICSDSIFEELASEFLSRSLEDDILNSLSLLIEGKASG 200

R 201 RDSVNFLNLLGVPSFDENQFPGSLRHPNIAPILGMVKASNYIDVVLPKTP 250

||||||||||||||||||||||||||||||||||||||||||||||||||

S 201 RDSVNFLNLLGVPSFDENQFPGSLRHPNIAPILGMVKASNYIDVVLPKTP 250

R 251 HTLENILHYSPDALKSDWHIRFLIYQLLSALAYIHGLGVSHGNICPSSVM 300

||||||||||||||||||||||||||||||||||||||||||||||||||

S 251 HTLENILHYSPDALKSDWHIRFLIYQLLSALAYIHGLGVSHGNICPSSVM 300

R 301 LTESCWSWLCICDKPGVGFNPSSRGNRCTTIIPEKVGCSIAGCPSQGLYA 350

||||||||||||||||||||||||||||||||||||||||||||||||||

S 301 LTESCWSWLCICDKPGVGFNPSSRGNRCTTIIPEKVGCSIAGCPSQGLYA 350

R 351 DLKLSPSIDWHRDFNQWWRGEISNFEYLLILNRLAGRRWGDHTFHTVMPW 400

||||||||||||||||||||||||||||||||||||||||||||||||||

S 351 DLKLSPSIDWHRDFNQWWRGEISNFEYLLILNRLAGRRWGDHTFHTVMPW 400

R 401 VIDFSMKPDENSDAGWRDLNKSKWRLAKGDEQLDFTYSTSEFPHHVSDEC 450

||||||||||||||||||||||||||||||||||||||||||||||||||

S 401 VIDFSMKPDENSDAGWRDLNKSKWRLAKGDEQLDFTYSTSEFPHHVSDEC 450

R 451 LSELAVCSYKARRLPLSVLRMAVRSVYEPNEYPSTMQRLYQWTPDECIPE 500

||||||||||||||||||||||||||||||||||||||||||||||||||

S 451 LSELAVCSYKARRLPLSVLRMAVRSVYEPNEYPSTMQRLYQWTPDECIPE 500

R 501 FYCDPQIFHSLHAGMTDLAVPSWACGPEEFIKLHRDALESDRVSCQLHHW 550

||||||||||||||||||||||||||||||||||||||||||||.|||||

S 501 FYCDPQIFHSLHAGMTDLAVPSWACGPEEFIKLHRDALESDRVSRQLHHW 550

R 551 IDITFGYKMVGQAAVAAKNVMLPSSEPMMPRSTGRRQLFTQPHPMRRGAI 600

||||||||||||||||||||||||||||||||||||||||||||||||||

S 551 IDITFGYKMVGQAAVAAKNVMLPSSEPMMPRSTGRRQLFTQPHPMRRGAI 600

R 601 PKPCDSTNGSSLYQGKMNELSSDSSVLFETAYLQELEDASAFCEHAMHLS 650

||||||||||||||||||||||||||||||||||||||||||||||||||

S 601 PKPCDSTNGSSLYQGKMNELSSDSSVLFETAYLQELEDASAFCEHAMHLS 650

R 651 ALYGYHLDSVKDIAPVEESSGEHVKKSVTLSDTKKNQWLRHIDTNYLLEH 700

||||||||||||||||||||||||||||||||||||||||||||||||||

S 651 ALYGYHLDSVKDIAPVEESSGEHVKKSVTLSDTKKNQWLRHIDTNYLLEH 700

R 701 VEVLDEGSSGYQELLLWRQKSSCSKMFAEEIARDVFSVGCLLAELHLRKP 750

||||||||||||||||||||||||||||||||||||||||||||||||||

S 701 VEVLDEGSSGYQELLLWRQKSSCSKMFAEEIARDVFSVGCLLAELHLRKP 750

R 751 LFDPTSLAVYLDSGLLPGLMHELPPHTRLLVEACIQKDCMRRPSAKCLLE 800

|||||||||||||

S 751 LFDPTSLAVYLDS------------------------------------- 763

R 801 SPYFPTTVKASYLFLAPLQLLAKGGSCLHYAANFAKQGVLKAMGTFAAEM 850

|||||||||||||||||||||||||||||||||||||||||||

S 764 -------VKASYLFLAPLQLLAKGGSCLHYAANFAKQGVLKAMGTFAAEM 806

R 851 CAPYCLSLVVTPLSDTEAEWAYTLLKEFIKSLTPKAVKRIVLPAIQRILQ 900

||||||||||||||||||||||||||||||||||||||||||||||||||

S 807 CAPYCLSLVVTPLSDTEAEWAYTLLKEFIKSLTPKAVKRIVLPAIQRILQ 856

R 901 ASYSHLKVSILQDSFVHEIWNQTGKQAYLETVHPLVILNLHAAAHKSSAA 950

||||||||||||||||||||||||||||||||||||||||||||||||||

S 857 ASYSHLKVSILQDSFVHEIWNQTGKQAYLETVHPLVILNLHAAAHKSSAA 906

R 951 AASVLLIGSSEELGIPITTHQTILPLIQCFGKGLSSDGIDVLVRIGGLLG 1000

||||||||||||||||||||||||||||||||||||||||||||||||||

S 907 AASVLLIGSSEELGIPITTHQTILPLIQCFGKGLSSDGIDVLVRIGGLLG 956

R 1001 ESFIVRQMLPLLKHVFHSCIDISHMNKPEPVHSWSAFALIDCLMTIDGLV 1050

||||||||||||||||||||||||||||||||||||||||||||||||||

S 957 ESFIVRQMLPLLKHVFHSCIDISHMNKPEPVHSWSAFALIDCLMTIDGLV 1006

R 1051 AFLPREVVAKELIEDKSCLHVLVLMQTSLEYRVLQVAATTLMAFCQRIGP 1100

||||||||||||||||||||||||||||||||||||||||||||||||||

S 1007 AFLPREVVAKELIEDKSCLHVLVLMQTSLEYRVLQVAATTLMAFCQRIGP 1056

R 1101 DLTALHVLPQLKELFDELAFSPKTANASTSFGRRLKGSKPKIDGALIESR 1150

||||||||||||||||||||||||||||||||||||||||||||||||||

S 1057 DLTALHVLPQLKELFDELAFSPKTANASTSFGRRLKGSKPKIDGALIESR 1106

R 1151 MDLVLLLYPSFASLLGIEKLRQCCATWLLLEQYLLQYHNWKWEHTGELSR 1200

||||||||||||||||||||||||||||||||||||||||||||||||||

S 1107 MDLVLLLYPSFASLLGIEKLRQCCATWLLLEQYLLQYHNWKWEHTGELSR 1156

R 1201 NGSDTILSKRNAFRKGSTSEYSPAKLLLNGVGWSIPQSQGSRSAKNLMPQ 1250

||||||||||||||||||||||||||||||||||||||||||||||||||

S 1157 NGSDTILSKRNAFRKGSTSEYSPAKLLLNGVGWSIPQSQGSRSAKNLMPQ 1206

R 1251 KRFFEMHQSPAEMHAATSNFKFEPWFWFPSPAASWDGPDFLGRAGGVKDE 1300

||||||||||||||||||||||||||||||||||||||||||||||||||

S 1207 KRFFEMHQSPAEMHAATSNFKFEPWFWFPSPAASWDGPDFLGRAGGVKDE 1256

R 1301 HPWKIRASVIYSVRAHPGALRYLAVCPDECTVFTAGIGAGFKGTVQKWEL 1350

||||||||||||||||||||||||||||||||||||||||||||||||||

S 1257 HPWKIRASVIYSVRAHPGALRYLAVCPDECTVFTAGIGAGFKGTVQKWEL 1306

R 1351 TRINCVSGYYGHEEVVNDICVLSSSGRVASCDGTIHVWNSRTGKLISVYS 1400

||||||||||||||||||||||||||||||||||||||||||||||||||

S 1307 TRINCVSGYYGHEEVVNDICVLSSSGRVASCDGTIHVWNSRTGKLISVYS 1356

R 1401 EPSVDSAHSASPPSSSSRVNVDQVNMLSSNTLSGGILTGAFDGSLYTCMH 1450

||||||||||||||||||||||||||||||||||||||||||||||||||

S 1357 EPSVDSAHSASPPSSSSRVNVDQVNMLSSNTLSGGILTGAFDGSLYTCMH 1406

R 1451 QTEFGEKLVVGTGNGSLRFIDVVRLQKLHLWRGDSTESGYPSLVSTICSC 1500

||||||||||||||||||||||||||||||||||||||||||||||||||

S 1407 QTEFGEKLVVGTGNGSLRFIDVVRLQKLHLWRGDSTESGYPSLVSTICSC 1456

R 1501 GSDKMQPDGASSPSWIAAGLSSGHCRLFDARSGNVIASWKAHDGYVTKLA 1550

||||||||||||||||||||||||||||||||||||||||||||||||||

S 1457 GSDKMQPDGASSPSWIAAGLSSGHCRLFDARSGNVIASWKAHDGYVTKLA 1506

R 1551 APEDHLLVSSSLDRTLRIWDLRRNWPSQPTILKGHTDGVSSFSVWGQDVI 1600

||||||||||||||||||||||||||||||||||||||||||||||||||

S 1507 APEDHLLVSSSLDRTLRIWDLRRNWPSQPTILKGHTDGVSSFSVWGQDVI 1556

R 1601 SIARNKIGLSSLSKSGDEDGQQAVTCQKLYMADHGARNFSVLSSISILPF 1650

||||||||||||||||||||||||||||||||||||||||||||||||||

S 1557 SIARNKIGLSSLSKSGDEDGQQAVTCQKLYMADHGARNFSVLSSISILPF 1606

R 1651 SRLFLVGTEDGYLRICC 1667

|||||||||||||||||

S 1607 SRLFLVGTEDGYLRICC 1623

Aligned_sequences: 2

1: **Par.chr1R_long.6.117**

2: **Par.chr1S_long.6.40**

Matrix: EDNAFULL

Gap_penalty: 10.0

Extend_penalty: 0.5

Length: 5004

Identity: 4866/5004 (97.2%)

Similarity: 4866/5004 (97.2%)

Gaps: 132/5004 ( 2.6%)

Score: 24230.5

Green color: START CODON

Red color: STOP CODON

R 1 ATGGCGCACGAAATGTGCTTCGACTGCCTCCACCGTCGGATTCAATCCGA 50

||||||||||||||||||||||||||||||||||||||||||||||||||

S 1 ATGGCGCACGAAATGTGCTTCGACTGCCTCCACCGTCGGATTCAATCCGA 50

R 51 CTTCTCCGAAAAGCTCGTTTTGGTCTACGGGCTCTCCGATTCCGCTTTCC 100

||||||||||||||||||||||||||||||||||||||||||||||||||

S 51 CTTCTCCGAAAAGCTCGTTTTGGTCTACGGGCTCTCCGATTCCGCTTTCC 100

R 101 CTTTCGGCTCCACTGCCGTCGTTCAGTTGTGTAATTCAAGTGGGGAAACT 150

||||||||||||||||||||||||||||||||||||||||||||||||||

S 101 CTTTCGGCTCCACTGCCGTCGTTCAGTTGTGTAATTCAAGTGGGGAAACT 150

R 151 GCTTCAGCTCCTCAGTTTTTGCTAAGCTATCTGCCCAGTCATGAACAAGA 200

||||||||||||||||||||||||||||||||||||||||||||||||||

S 151 GCTTCAGCTCCTCAGTTTTTGCTAAGCTATCTGCCCAGTCATGAACAAGA 200

R 201 TTGCTTGACCAAATATGTTAATGAATATATAGGTGATAATGCTGAAGGCT 250

||||||||||||||||||||||||||||||||||||||||||||||||||

S 201 TTGCTTGACCAAATATGTTAATGAATATATAGGTGATAATGCTGAAGGCT 250

R 251 GCAGTGATAGTGTGATTGCTAATACGATCACTTCCTCTGAAGTGACTCGA 300

||||||||||||||||||||||||||||||||||||||||||||||||||

S 251 GCAGTGATAGTGTGATTGCTAATACGATCACTTCCTCTGAAGTGACTCGA 300

R 301 GATGAAGAGGAAGTTAGTTCTGATGTTAACAATGACCAAAAACCAAAGTT 350

||||||||||||||||||||||||||||||||||||||||||||||||||

S 301 GATGAAGAGGAAGTTAGTTCTGATGTTAACAATGACCAAAAACCAAAGTT 350

R 351 GGATTCTTTATTGAACATGTCTCCGTGTTTATCAAATGGTGGCACAAAAA 400

||||||||||||||||||||||||||||||||||||||||||||||||||

S 351 GGATTCTTTATTGAACATGTCTCCGTGTTTATCAAATGGTGGCACAAAAA 400

R 401 CATTTCTACAGAGTTCTACCTGTAAACATTCCAGTAGGTTTTCTTGCTCG 450

||||||||||||||||||||||||||||||||||||||||||||||||||

S 401 CATTTCTACAGAGTTCTACCTGTAAACATTCCAGTAGGTTTTCTTGCTCG 450

R 451 AGGGTGATCAGCGGGTTGGCACCGATTACTCACGTTGGCATTTGTTCAGA 500

||||||||||||||||||||||||||||||||||||||||||||||||||

S 451 AGGGTGATCAGCGGGTTGGCACCGATTACTCACGTTGGCATTTGTTCAGA 500

R 501 CTCCATTTTCGAAGAGCTTGCTTCGGAATTTTTGTCCAGGTCTCTGGAAG 550

||||||||||||||||||||||||||||||||||||||||||||||||||

S 501 CTCCATTTTCGAAGAGCTTGCTTCGGAATTTTTGTCCAGGTCTCTGGAAG 550

R 551 ATAACATTTTGAACTCACTTAGTCTCTTGATTGAAGGGAAAGCTTCAGGA 600

||.|||||||||||||||||||||||||||||||||||||||||||||||

S 551 ATGACATTTTGAACTCACTTAGTCTCTTGATTGAAGGGAAAGCTTCAGGA 600

R 601 AGAGACAGTGTAAATTTTCTTAACTTACTTGGGGTACCGTCTTTTGATGA 650

||||||||||||||||||||||||||||||||||||||||||||||||||

S 601 AGAGACAGTGTAAATTTTCTTAACTTACTTGGGGTACCGTCTTTTGATGA 650

R 651 GAATCAGTTCCCTGGCTCACTGAGGCATCCAAATATTGCTCCCATACTTG 700

||||||||||||||||||||||||||||||||||||||||||||||||||

S 651 GAATCAGTTCCCTGGCTCACTGAGGCATCCAAATATTGCTCCCATACTTG 700

R 701 GGATGGTTAAGGCATCCAATTATATTGATGTAGTTCTTCCCAAAACTCCA 750

||||||||||||||||||||||||||||||||||||||||||||||||||

S 701 GGATGGTTAAGGCATCCAATTATATTGATGTAGTTCTTCCCAAAACTCCA 750

R 751 CATACCTTGGAAAACATTCTCCATTATAGTCCCGATGCCTTAAAGTCTGA 800

||||||||||||||||||||||||||||||||||||||||||||||||||

S 751 CATACCTTGGAAAACATTCTCCATTATAGTCCCGATGCCTTAAAGTCTGA 800

R 801 TTGGCATATAAGGTTTCTAATATACCAGTTACTCTCGGCTCTAGCTTATA 850

||||||||||||||||||||||||||||||||||||||||||||||||||

S 801 TTGGCATATAAGGTTTCTAATATACCAGTTACTCTCGGCTCTAGCTTATA 850

R 851 TTCATGGTTTAGGGGTTTCCCATGGCAACATATGCCCATCCAGTGTGATG 900

||||||||||||||||||||||||||||||||||||||||||||||||||

S 851 TTCATGGTTTAGGGGTTTCCCATGGCAACATATGCCCATCCAGTGTGATG 900

R 901 CTTACCGAATCATGCTGGTCTTGGCTGTGTATTTGTGATAAGCCCGGGGT 950

||||||||||||||||||||||||||||||||||||||||||||||||||

S 901 CTTACCGAATCATGCTGGTCTTGGCTGTGTATTTGTGATAAGCCCGGGGT 950

R 951 AGGATTTAATCCAAGTTCTAGAGGTAATAGATGCACAACTATTATACCTG 1000

||||||||||||||||||||||||||||||||||||||||||||||||||

S 951 AGGATTTAATCCAAGTTCTAGAGGTAATAGATGCACAACTATTATACCTG 1000

R 1001 AAAAGGTAGGTTGCTCTATAGCTGGTTGTCCTTCTCAAGGTCTTTATGCT 1050

||||||||||||||||||||||||||||||||||||||||||||||||||

S 1001 AAAAGGTAGGTTGCTCTATAGCTGGTTGTCCTTCTCAAGGTCTTTATGCT 1050

R 1051 GATTTGAAGCTTTCCCCATCTATTGACTGGCATCGTGACTTCAATCAATG 1100

||||||||||||||||||||||||||||||||||||||||||||||||||

S 1051 GATTTGAAGCTTTCCCCATCTATTGACTGGCATCGTGACTTCAATCAATG 1100

R 1101 GTGGAGGGGGGAGATTAGTAATTTTGAGTATCTGCTCATCTTGAATAGAT 1150

||||||||||||||||||||||||||||||||||||||||||||||||||

S 1101 GTGGAGGGGGGAGATTAGTAATTTTGAGTATCTGCTCATCTTGAATAGAT 1150

R 1151 TAGCTGGGCGAAGGTGGGGTGACCACACATTTCATACAGTGATGCCATGG 1200

||||||||||||||||||||||||||||||||||||||||||||||||||

S 1151 TAGCTGGGCGAAGGTGGGGTGACCACACATTTCATACAGTGATGCCATGG 1200

R 1201 GTAATAGATTTTAGCATGAAGCCTGATGAGAATTCAGATGCAGGGTGGCG 1250

||||||||||||||||||||||||||||||||||||||||||||||||||

S 1201 GTAATAGATTTTAGCATGAAGCCTGATGAGAATTCAGATGCAGGGTGGCG 1250

R 1251 GGATCTAAACAAGAGTAAATGGAGGTTGGCAAAAGGTGATGAACAGTTGG 1300

||||||||||||||||||||||||||||||||||||||||||||||||||

S 1251 GGATCTAAACAAGAGTAAATGGAGGTTGGCAAAAGGTGATGAACAGTTGG 1300

R 1301 ACTTCACCTATTCAACATCAGAGTTCCCACATCATGTATCTGATGAATGT 1350

||||||||||||||||||||||||||||||||||||||||||||||||||

S 1301 ACTTCACCTATTCAACATCAGAGTTCCCACATCATGTATCTGATGAATGT 1350

R 1351 CTTTCTGAATTGGCTGTCTGCAGTTATAAAGCAAGGAGGTTACCTTTGAG 1400

||||||||||||||||||||||||||||||||||||||||||||||||||

S 1351 CTTTCTGAATTGGCTGTCTGCAGTTATAAAGCAAGGAGGTTACCTTTGAG 1400

R 1401 TGTCCTACGTATGGCTGTTCGCTCAGTCTATGAACCTAATGAATATCCTT 1450

||||||||||||||||||||||||||||||||||||||||||||||||||

S 1401 TGTCCTACGTATGGCTGTTCGCTCAGTCTATGAACCTAATGAATATCCTT 1450

R 1451 CTACCATGCAAAGACTCTATCAATGGACCCCTGATGAGTGCATTCCAGAG 1500

||||||||||||||||||||||||||||||||||||||||||||||||||

S 1451 CTACCATGCAAAGACTCTATCAATGGACCCCTGATGAGTGCATTCCAGAG 1500

R 1501 TTTTACTGCGATCCCCAAATTTTTCATTCACTTCATGCTGGTATGACTGA 1550

||||||||||||||||||||||||||||||||||||||||||||||||||

S 1501 TTTTACTGCGATCCCCAAATTTTTCATTCACTTCATGCTGGTATGACTGA 1550

R 1551 CTTGGCTGTACCTTCATGGGCATGTGGTCCTGAGGAATTCATTAAATTGC 1600

||||||||||||||||||||||||||||||||||||||||||||||||||

S 1551 CTTGGCTGTACCTTCATGGGCATGTGGTCCTGAGGAATTCATTAAATTGC 1600

R 1601 ATCGTGATGCTTTAGAAAGCGATCGGGTTTCATGCCAACTCCATCATTGG 1650

||||||||||||||||||||||||||||||||.|||||||||||||||||

S 1601 ATCGTGATGCTTTAGAAAGCGATCGGGTTTCACGCCAACTCCATCATTGG 1650

R 1651 ATTGATATCACCTTTGGTTACAAGATGGTAGGTCAGGCAGCCGTTGCTGC 1700

||||||||||||||||||||||||||||||||||||||||||||||||||

S 1651 ATTGATATCACCTTTGGTTACAAGATGGTAGGTCAGGCAGCCGTTGCTGC 1700

R 1701 AAAGAATGTAATGCTTCCTTCATCAGAGCCCATGATGCCAAGGTCAACGG 1750

||||||||||||||||||||||||||||||||||||||||||||||||||

S 1701 AAAGAATGTAATGCTTCCTTCATCAGAGCCCATGATGCCAAGGTCAACGG 1750

R 1751 GACGCCGTCAGCTTTTTACTCAACCTCACCCTATGCGTCGAGGTGCAATA 1800

||||||||||||||||||||||||||||||||||||||||||||||||||

S 1751 GACGCCGTCAGCTTTTTACTCAACCTCACCCTATGCGTCGAGGTGCAATA 1800

R 1801 CCGAAACCATGTGATAGCACCAATGGGTCATCTTTGTATCAAGGGAAAAT 1850

||||||||||||||||||||||||||||||||||||||||||||||||||

S 1801 CCGAAACCATGTGATAGCACCAATGGGTCATCTTTGTATCAAGGGAAAAT 1850

R 1851 GAATGAATTAAGTAGTGACAGTTCTGTCCTGTTTGAAACTGCTTACTTGC 1900

||||||||||||||||||||||||||||||||||||||||||||||||||

S 1851 GAATGAATTAAGTAGTGACAGTTCTGTCCTGTTTGAAACTGCTTACTTGC 1900

R 1901 AAGAGTTAGAAGATGCATCTGCCTTTTGTGAACATGCTATGCATTTGAGT 1950

||||||||||||||||||||||||||||||||||||||||||||||||||

S 1901 AAGAGTTAGAAGATGCATCTGCCTTTTGTGAACATGCTATGCATTTGAGT 1950

R 1951 GCCCTCTATGGCTATCATCTAGACTCCGTGAAGGACATTGCTCCTGTAGA 2000

||||||||||||||||||||||||||||||||||||||||||||||||||

S 1951 GCCCTCTATGGCTATCATCTAGACTCCGTGAAGGACATTGCTCCTGTAGA 2000

R 2001 AGAGTCATCGGGCGAGCATGTTAAGAAAAGTGTAACTCTATCTGACACTA 2050

||||||||||||||||||||||||||||||||||||||||||||||||||

S 2001 AGAGTCATCGGGCGAGCATGTTAAGAAAAGTGTAACTCTATCTGACACTA 2050

R 2051 AGAAAAACCAGTGGTTGCGACATATTGATACAAATTATCTTCTTGAGCAT 2100

||||||||||||||||||||||||||||||||||||||||||||||||||

S 2051 AGAAAAACCAGTGGTTGCGACATATTGATACAAATTATCTTCTTGAGCAT 2100

R 2101 GTTGAGGTGTTGGATGAAGGTTCCAGTGGATATCAAGAGCTATTGCTTTG 2150

||||||||||||||||||||||||||||||||||||||||||||||||||

S 2101 GTTGAGGTGTTGGATGAAGGTTCCAGTGGATATCAAGAGCTATTGCTTTG 2150

R 2151 GAGACAGAAATCATCATGTTCAAAGATGTTTGCTGAAGAAATTGCAAGGG 2200

||||||||||||||||||||||||||||||||||||||||||||||||||

S 2151 GAGACAGAAATCATCATGTTCAAAGATGTTTGCTGAAGAAATTGCAAGGG 2200

R 2201 ACGTATTTTCTGTTGGTTGTCTCTTAGCAGAACTTCATTTGAGGAAGCCA 2250

||||||||||||||||||||||||||||||||||||||||||||||||||

S 2201 ACGTATTTTCTGTTGGTTGTCTCTTAGCAGAACTTCATTTGAGGAAGCCA 2250

R 2251 CTTTTTGATCCAACCTCATTGGCCGTGTACTTGGACAGTGGTTTATTACC 2300

|||||||||||||||||||||||||||||||||||||||

S 2251 CTTTTTGATCCAACCTCATTGGCCGTGTACTTGGACAGT----------- 2289

R 2301 TGGACTGATGCATGAACTTCCTCCTCACACCAGATTACTTGTCGAAGCCT 2350

S 2290 -------------------------------------------------- 2289

R 2351 GCATTCAAAAGGATTGCATGAGGAGACCATCGGCTAAGTGTCTTTTGGAA 2400

S 2290 -------------------------------------------------- 2289

R 2401 TCACCTTATTTTCCTACCACAGTCAAGGCTTCCTACTTGTTTCTTGCCCC 2450

|||||||||||||||||||||||||||||

S 2290 ---------------------GTCAAGGCTTCCTACTTGTTTCTTGCCCC 2318

R 2451 CCTCCAGCTTCTGGCTAAAGGTGGATCTTGCCTTCATTATGCTGCGAATT 2500

||||||||||||||||||||||||||||||||||||||||||||||||||

S 2319 CCTCCAGCTTCTGGCTAAAGGTGGATCTTGCCTTCATTATGCTGCGAATT 2368

R 2501 TTGCAAAGCAAGGAGTCCTGAAGGCAATGGGAACATTTGCTGCTGAAATG 2550

||||||||||||||||||||||||||||||||||||||||||||||||||

S 2369 TTGCAAAGCAAGGAGTCCTGAAGGCAATGGGAACATTTGCTGCTGAAATG 2418

R 2551 TGTGCTCCTTATTGCTTATCACTTGTGGTGACTCCTTTATCAGATACTGA 2600

||||||||||||||||||||||||||||||||||||||||||||||||||

S 2419 TGTGCTCCTTATTGCTTATCACTTGTGGTGACTCCTTTATCAGATACTGA 2468

R 2601 AGCTGAATGGGCCTATACATTACTGAAAGAATTTATCAAAAGTTTAACAC 2650

||||||||||||||||||||||||||||||||||||||||||||||||||

S 2469 AGCTGAATGGGCCTATACATTACTGAAAGAATTTATCAAAAGTTTAACAC 2518

R 2651 CTAAAGCAGTGAAACGAATAGTCTTGCCTGCCATCCAGAGGATTTTACAG 2700

||||||||||||||||||||||||||||||||||||||||||||||||||

S 2519 CTAAAGCAGTGAAACGAATAGTCTTGCCTGCCATCCAGAGGATTTTACAG 2568

R 2701 GCTAGCTATTCACACCTAAAGGTTTCTATTCTACAAGACTCTTTTGTTCA 2750

||||||||||||||||||||||||||||||||||||||||||||||||||

S 2569 GCTAGCTATTCACACCTAAAGGTTTCTATTCTACAAGACTCTTTTGTTCA 2618

R 2751 TGAGATATGGAATCAAACTGGCAAACAAGCATATCTAGAAACTGTACATC 2800

||||||||||||||||||||||||||||||||||||||||||||||||||

S 2619 TGAGATATGGAATCAAACTGGCAAACAAGCATATCTAGAAACTGTACATC 2668

R 2801 CTCTTGTGATTTTGAACTTGCATGCTGCTGCTCATAAGAGTTCAGCAGCT 2850

||||||||||||||||||||||||||||||||||||||||||||||||||

S 2669 CTCTTGTGATTTTGAACTTGCATGCTGCTGCTCATAAGAGTTCAGCAGCT 2718

R 2851 GCCGCTTCCGTGCTGCTGATTGGCTCAAGTGAAGAGCTTGGTATACCTAT 2900

||||||||||||||||||||||||||||||||||||||||||||||||||

S 2719 GCCGCTTCCGTGCTGCTGATTGGCTCAAGTGAAGAGCTTGGTATACCTAT 2768

R 2901 TACCACTCATCAGACAATCTTGCCTCTGATTCAATGCTTTGGGAAAGGTC 2950

||||||||||||||||||||||||||||||||||||||||||||||||||

S 2769 TACCACTCATCAGACAATCTTGCCTCTGATTCAATGCTTTGGGAAAGGTC 2818

R 2951 TCAGTAGTGATGGAATTGACGTGCTGGTTCGAATTGGTGGTCTATTGGGA 3000

||||||||||||||||||||||||||||||||||||||||||||||||||

S 2819 TCAGTAGTGATGGAATTGACGTGCTGGTTCGAATTGGTGGTCTATTGGGA 2868

R 3001 GAGAGCTTCATCGTCAGACAGATGCTACCATTGCTAAAACATGTATTTCA 3050

||||||||||||||||||||||||||||||||||||||||||||||||||

S 2869 GAGAGCTTCATCGTCAGACAGATGCTACCATTGCTAAAACATGTATTTCA 2918

R 3051 TTCCTGCATTGACATTTCACATATGAATAAACCTGAGCCTGTCCACAGCT 3100

||||||||||||||||||||||||||||||||||||||||||||||||||

S 2919 TTCCTGCATTGACATTTCACATATGAATAAACCTGAGCCTGTCCACAGCT 2968

R 3101 GGAGTGCTTTCGCTCTTATTGATTGTTTAATGACAATAGATGGCCTAGTT 3150

||||||||||||||||||||||||||||||||||||||||||||||||||

S 2969 GGAGTGCTTTCGCTCTTATTGATTGTTTAATGACAATAGATGGCCTAGTT 3018

R 3151 GCATTCTTGCCAAGGGAGGTGGTTGCAAAGGAGCTAATTGAAGATAAAAG 3200

||||||||||||||||||||||||||||||||||||||||||||||||||

S 3019 GCATTCTTGCCAAGGGAGGTGGTTGCAAAGGAGCTAATTGAAGATAAAAG 3068

R 3201 TTGCCTGCATGTTCTGGTTCTAATGCAGACAAGTTTAGAATATAGAGTGC 3250

||||||||||||||||||||||||||||||||||||||||||||||||||

S 3069 TTGCCTGCATGTTCTGGTTCTAATGCAGACAAGTTTAGAATATAGAGTGC 3118

R 3251 TTCAGGTTGCTGCTACAACTCTAATGGCATTTTGTCAGCGGATAGGACCG 3300

||||||||||||||||||||||||||||||||||||||||||||||||||

S 3119 TTCAGGTTGCTGCTACAACTCTAATGGCATTTTGTCAGCGGATAGGACCG 3168

R 3301 GATTTGACAGCATTGCATGTTTTGCCACAACTTAAAGAGCTATTTGATGA 3350

||||||||||||||||||||||||||||||||||||||||||||||||||

S 3169 GATTTGACAGCATTGCATGTTTTGCCACAACTTAAAGAGCTATTTGATGA 3218

R 3351 GCTTGCTTTCTCACCAAAAACTGCCAATGCTTCTACTTCCTTTGGCAGAA 3400

||||||||||||||||||||||||||||||||||||||||||||||||||

S 3219 GCTTGCTTTCTCACCAAAAACTGCCAATGCTTCTACTTCCTTTGGCAGAA 3268

R 3401 GGTTGAAGGGTTCAAAGCCAAAAATTGATGGGGCTCTGATTGAAAGTCGT 3450

||||||||||||||||||||||||||||||||||||||||||||||||||

S 3269 GGTTGAAGGGTTCAAAGCCAAAAATTGATGGGGCTCTGATTGAAAGTCGT 3318

R 3451 ATGGACCTTGTGTTGCTTCTGTATCCTTCTTTTGCATCCCTTCTTGGCAT 3500

||||||||||||||||||||||||||||||||||||||||||||||||||

S 3319 ATGGACCTTGTGTTGCTTCTGTATCCTTCTTTTGCATCCCTTCTTGGCAT 3368

R 3501 AGAGAAGCTTCGCCAGTGTTGTGCAACATGGTTATTACTTGAGCAATATC 3550

||||||||||||||||||||||||||||||||||||||||||||||||||

S 3369 AGAGAAGCTTCGCCAGTGTTGTGCAACATGGTTATTACTTGAGCAATATC 3418

R 3551 TCCTACAGTATCATAACTGGAAGTGGGAACACACAGGAGAATTGTCTCGA 3600

||||||||||||||||||||||||||||||||||||||||||||||||||

S 3419 TCCTACAGTATCATAACTGGAAGTGGGAACACACAGGAGAATTGTCTCGA 3468

R 3601 AATGGTTCAGACACTATACTCAGTAAAAGAAATGCATTCAGAAAGGGCTC 3650

||||||||||||||||||||||||||||||||||||||||||||||||||

S 3469 AATGGTTCAGACACTATACTCAGTAAAAGAAATGCATTCAGAAAGGGCTC 3518

R 3651 AACTTCTGAATACAGTCCTGCTAAGCTGTTGCTCAATGGGGTTGGATGGT 3700

||||||||||||||||||||||||||||||||||||||||||||||||||

S 3519 AACTTCTGAATACAGTCCTGCTAAGCTGTTGCTCAATGGGGTTGGATGGT 3568

R 3701 CAATTCCGCAATCACAAGGGTCTAGAAGTGCCAAAAACTTGATGCCTCAA 3750

||||||||||||||||||||||||||||||||||||||||||||||||||

S 3569 CAATTCCGCAATCACAAGGGTCTAGAAGTGCCAAAAACTTGATGCCTCAA 3618

R 3751 AAACGGTTCTTTGAAATGCATCAGAGTCCAGCTGAAATGCATGCAGCAAC 3800

||||||||||||||||||||||||||||||||||||||||||||||||||

S 3619 AAACGGTTCTTTGAAATGCATCAGAGTCCAGCTGAAATGCATGCAGCAAC 3668

R 3801 ATCAAATTTTAAATTCGAGCCCTGGTTTTGGTTCCCTAGTCCAGCTGCTA 3850

||||||||||||||||||||||||||||||||||||||||||||||||||

S 3669 ATCAAATTTTAAATTCGAGCCCTGGTTTTGGTTCCCTAGTCCAGCTGCTA 3718

R 3851 GCTGGGACGGGCCTGATTTTCTTGGGCGTGCTGGGGGTGTGAAAGATGAA 3900

||||||||||||||||||||||||||||||||||||||||||||||||||

S 3719 GCTGGGACGGGCCTGATTTTCTTGGGCGTGCTGGGGGTGTGAAAGATGAA 3768

R 3901 CATCCATGGAAGATCAGAGCATCTGTCATATACTCAGTCCGTGCGCATCC 3950

||||||||||||||||||||||||||||||||||||||||||||||||||

S 3769 CATCCATGGAAGATCAGAGCATCTGTCATATACTCAGTCCGTGCGCATCC 3818

R 3951 TGGGGCCTTACGGTATTTAGCCGTCTGTCCAGATGAATGTACGGTTTTTA 4000

||||||||||||||||||||||||||||||||||||||||||||||||||

S 3819 TGGGGCCTTACGGTATTTAGCCGTCTGTCCAGATGAATGTACGGTTTTTA 3868

R 4001 CTGCAGGGATTGGTGCAGGGTTCAAGGGAACTGTTCAGAAATGGGAGCTG 4050

||||||||||||||||||||||||||||||||||||||||||||||||||

S 3869 CTGCAGGGATTGGTGCAGGGTTCAAGGGAACTGTTCAGAAATGGGAGCTG 3918

R 4051 ACTAGAATTAATTGTGTATCGGGATACTATGGCCATGAGGAGGTTGTGAA 4100

||||||||||||||||||||.|||||||||||||||||||||||||||||

S 3919 ACTAGAATTAATTGTGTATCAGGATACTATGGCCATGAGGAGGTTGTGAA 3968

R 4101 CGATATTTGTGTCTTGTCGTCTAGTGGAAGAGTTGCGTCTTGTGATGGGA 4150

||||||||||||||||||.|||||||||||||||||||||||||||||||

S 3969 CGATATTTGTGTCTTGTCATCTAGTGGAAGAGTTGCGTCTTGTGATGGGA 4018

R 4151 CAATACATGTTTGGAATAGCCGAACTGGGAAATTAATTTCAGTATATTCT 4200

||||||||||||||||||||||||||||||||||||||||||||||||||

S 4019 CAATACATGTTTGGAATAGCCGAACTGGGAAATTAATTTCAGTATATTCT 4068

R 4201 GAACCATCTGTGGATTCTGCACATAGTGCAAGCCCTCCATCTTCTTCCTC 4250

||||||||||||||||||||||||||||||||||||||||||||||||||

S 4069 GAACCATCTGTGGATTCTGCACATAGTGCAAGCCCTCCATCTTCTTCCTC 4118

R 4251 CAGGGTCAATGTGGACCAGGTCAATATGCTCAGTTCCAATACACTGTCTG 4300

||||||||||||||||||||||||||||||||||||||||||||||||||

S 4119 CAGGGTCAATGTGGACCAGGTCAATATGCTCAGTTCCAATACACTGTCTG 4168

R 4301 GTGGAATATTGACTGGCGCATTTGATGGTAGCTTGTACACTTGTATGCAT 4350

||||||||||||||||||||||||||||||||||||||||||||||||||

S 4169 GTGGAATATTGACTGGCGCATTTGATGGTAGCTTGTACACTTGTATGCAT 4218

R 4351 CAGACAGAATTTGGTGAAAAGCTTGTAGTTGGCACTGGAAATGGTTCTCT 4400

||||||||||||||||||||||||||||||||||||||||||||||||||

S 4219 CAGACAGAATTTGGTGAAAAGCTTGTAGTTGGCACTGGAAATGGTTCTCT 4268

R 4401 CAGGTTCATTGATGTTGTCCGATTACAAAAGCTTCACCTTTGGAGGGGGG 4450

||||||||||||||||||||||||||||||||||||||||||||||||||

S 4269 CAGGTTCATTGATGTTGTCCGATTACAAAAGCTTCACCTTTGGAGGGGGG 4318

R 4451 ATTCTACGGAGTCTGGTTATCCTTCCCTTGTTTCTACCATATGCTCCTGT 4500

||||||||||||||||||||||||||||||||||||||||||||||||||

S 4319 ATTCTACGGAGTCTGGTTATCCTTCCCTTGTTTCTACCATATGCTCCTGT 4368

R 4501 GGGTCTGACAAAATGCAACCAGATGGAGCTTCTTCACCATCTTGGATTGC 4550

||||||||||||||||||||||||||||||||||||||||||||||||||

S 4369 GGGTCTGACAAAATGCAACCAGATGGAGCTTCTTCACCATCTTGGATTGC 4418

R 4551 AGCTGGATTGAGTTCTGGTCATTGTAGGTTATTTGATGCAAGGAGTGGCA 4600

||||||||||||||||||||||||||||||||||||||||||||||||||

S 4419 AGCTGGATTGAGTTCTGGTCATTGTAGGTTATTTGATGCAAGGAGTGGCA 4468

R 4601 ATGTTATTGCCTCTTGGAAGGCTCATGATGGATATGTGACAAAGTTGGCT 4650

||||||||||||||||||||||||||||||||||.|||||||||||||||

S 4469 ATGTTATTGCCTCTTGGAAGGCTCATGATGGATACGTGACAAAGTTGGCT 4518

R 4651 GCACCAGAGGACCATTTACTTGTGTCAAGCTCTCTTGACAGGACTTTAAG 4700

||||||||||||||||||||||||||||||||||||||||||||||||||

S 4519 GCACCAGAGGACCATTTACTTGTGTCAAGCTCTCTTGACAGGACTTTAAG 4568

R 4701 AATTTGGGACTTGAGAAGGAATTGGCCTTCTCAGCCCACAATTCTTAAGG 4750

||||||||||||||||||||||||||||||||||||||||||||||||||

S 4569 AATTTGGGACTTGAGAAGGAATTGGCCTTCTCAGCCCACAATTCTTAAGG 4618

R 4751 GTCATACGGATGGCGTATCTTCGTTTTCTGTGTGGGGCCAAGATGTTATT 4800

||||||||||||||||||||||||||||||||||||||||||||||||||

S 4619 GTCATACGGATGGCGTATCTTCGTTTTCTGTGTGGGGCCAAGATGTTATT 4668

R 4801 TCAATTGCCAGAAATAAAATTGGACTTTCTTCTTTATCCAAATCTGGTGA 4850

||||||||||||||||||||||||||||||||||||||||||||||||||

S 4669 TCAATTGCCAGAAATAAAATTGGACTTTCTTCTTTATCCAAATCTGGTGA 4718

R 4851 TGAGGATGGGCAGCAGGCTGTCACATGCCAAAAACTCTACATGGCTGATC 4900

|||||||||||||||||||||.||||||||||||||||||||||||||||

S 4719 TGAGGATGGGCAGCAGGCTGTTACATGCCAAAAACTCTACATGGCTGATC 4768

R 4901 ATGGAGCAAGAAACTTTTCAGTTTTGTCAAGCATAAGTATTTTACCCTTC 4950

||||||||||||||||||||||||||||||||||||||||||||||||||

S 4769 ATGGAGCAAGAAACTTTTCAGTTTTGTCAAGCATAAGTATTTTACCCTTC 4818

R 4951 TCAAGATTGTTTCTTGTGGGTACAGAAGATGGCTATCTGAGAATATGTTG 5000

||||||||||||||||||||||||||||||||||||||||||||||||||

S 4819 TCAAGATTGTTTCTTGTGGGTACAGAAGATGGCTATCTGAGAATATGTTG 4868

R 5001 CTGA 5004

||||

S 4869 CTGA 4872

**Pa43 - Mediator of RNA polymerase II transcription subunit 21-like**

Aligned_sequences: 2

1: **Par.chr1R_long.6.120**

2: **Par.chr1S_long.6.96**

Matrix: EBLOSUM62

Gap_penalty: 10.0

Extend_penalty: 0.5

Length: 167

Identity: 128/167 (76.6%)

Similarity: 128/167 (76.6%)

Gaps: 36/167 (21.6%)

Score: 602.5

R 1 MDAISQLQEKVNTIATIAFTTIGTLQRDAPPVRISPNYPESGSGPTPAPA 50

||||||||||||||||||||||||||||||||||||||||||||||||||

S 1 MDAISQLQEKVNTIATIAFTTIGTLQRDAPPVRISPNYPESGSGPTPAPA 50

R 51 PNPNPNPTPTPAADSDADFAKQPKLMSAELVKAAKQFDALVAALPLSEGG 100

|||| ||||||||||||||||||||||||||||||||||||||||||

S 51 PNPN----PTPAADSDADFAKQPKLMSAELVKAAKQFDALVAALPLSEGG 96

R 101 EEAQLKRIAQLEAENDAVGQQLEKQLEAAGDESFYLVTFHRIFSFLQF-- 148

|||||||||||||||||||||||||||||..| ||.

S 97 EEAQLKRIAQLEAENDAVGQQLEKQLEAAERE-------------LQEVR 133

R 149 ----------------- 148

S 134 ELFGQAADHCLNLKKPE 150

Aligned_sequences: 2

1: **Par.chr1R_long.6.120**

2: **Par.chr1S_long.6.96**

Matrix: EDNAFULL

Gap_penalty: 10.0

Extend_penalty: 0.5

Length: 733

Identity: 451/733 (61.5%)

Similarity: 451/733 (61.5%)

Gaps: 280/733 (38.2%)

Score: 2088.0

Green color: START CODON

Red color: STOP CODON

R 1 ATGGATGCAATCAGCCAATTACAAGAGAAAGTGAATACAATCGCGACAAT 50

||||||||||||||||||||||||||||||||||||||||||||||||||

S 1 ATGGATGCAATCAGCCAATTACAAGAGAAAGTGAATACAATCGCGACAAT 50

R 51 CGCGTTCACTACCATCGGAACGCTGCAGAGGGATGCGCCGCCGGTCCGAA 100

||||||||||||||||||||||||||||||||||||||||||||||||||

S 51 CGCGTTCACTACCATCGGAACGCTGCAGAGGGATGCGCCGCCGGTCCGAA 100

R 101 TCTCTCCGAATTACCCAGAATCCGGATCTGGGCCGACTCCGGCTCCGGCT 150

||||||||||||||||||||||||||||||||||||||||||||||||||

S 101 TCTCTCCGAATTACCCAGAATCCGGATCTGGGCCGACTCCGGCTCCGGCT 150

R 151 CCGAATCCGAACCCGAACCCGACCCCGACCCCGGCGGCGGACAGTGACGC 200

||||||||||.|||||||||||||||||||||||||||

S 151 ------------CCGAACCCGAACCCGACCCCGGCGGCGGACAGTGACGC 188

R 201 GGATTTCGCGAAGCAACCCAAGCTGATGAGTGCTGAGCTAGTGAAGGCAG 250

||||||||||||||||||||||||||||||||||||||||||||||||||

S 189 GGATTTCGCGAAGCAACCCAAGCTGATGAGTGCTGAGCTAGTGAAGGCAG 238

R 251 CTAAGCAGTTTGATGCATTGGTGGCGGCACTTCCGTTGTCTGAGGGGGGA 300

|||||||||||||.||||||||||||||||||||||||||||||||||||

S 239 CTAAGCAGTTTGACGCATTGGTGGCGGCACTTCCGTTGTCTGAGGGGGGA 288

R 301 GAGGAAGCTCAGCTGAAGAGGATTGCACAACTTGAGGCTGAAAATGATGC 350

||||||||||||||||||||||||||||||||||||||||||||||||||

S 289 GAGGAAGCTCAGCTGAAGAGGATTGCACAACTTGAGGCTGAAAATGATGC 338

R 351 TGTGGGCCAACAACTTGAGAAGCAACTGGAAGCTGCAGGTGATGAGTCTT 400

||||||||||||||||||||||||||||||||||

S 339 TGTGGGCCAACAACTTGAGAAGCAACTGGAAGCT---------------- 372

R 401 TTTATTTGGTCACATTTCACAGAATTTTCAGTTTTCTTCAGTTTTAATGA 450

S 373 -------------------------------------------------- 372

R 451 TATTATTAGTTGATTGTTTTTAGGGTCTACAAAATTGTCATTTAGAACTT 500

S 373 -------------------------------------------------- 372

R 501 GAACTTTGGGTTTATAGTCTCGGATTGGTTAATCGCTTCGGTTTTAGGTT 550

S 373 -------------------------------------------------- 372

R 551 TTAGGTCTTAGAACCTAAAATCGAAACTCGACGATTCTATACCCTAAGTA 600

S 373 -------------------------------------------------- 372

R 601 ATCAACCAGGGTTAGATATTTGAATCACTGTGTCAATGTGGTTTTGATGG 650

S 373 -------------------------------------------------- 372

R 651 TGGCAGAGAGAGAATTGCAAGAGGTCAGAGAGTTGTTTGGACAAGCAGCA 700

||||||||||||||||||||||||||||||||||||||||||||||||

S 373 --GCAGAGAGAGAATTGCAAGAGGTCAGAGAGTTGTTTGGACAAGCAGCA 420

R 701 GATCACTGTTTGAACTTGAAGAAACCAGAATGA 733

|||||||||||||||||||||||||||||||||

S 421 GATCACTGTTTGAACTTGAAGAAACCAGAATGA 453

**Pa44 - Methyltransferase-like protein 13**

Aligned_sequences: 2

1: **Par.chr1R_long.6.106**

2: **Par.chr1S_long.6.112**

Matrix: EBLOSUM62

Gap_penalty: 10.0

Extend_penalty: 0.5

Length: 258

Identity: 247/258 (95.7%)

Similarity: 247/258 (95.7%)

Gaps: 11/258 ( 4.3%)

Score: 1314.0

R 1 MTMGTTTTQAYGEPWYWDNRYANESGPFDWYQKYQSLAPLINLYVPRHSN 50

||||||||||||||||||||||||||||||||||||||||||||||||||

S 1 MTMGTTTTQAYGEPWYWDNRYANESGPFDWYQKYQSLAPLINLYVPRHSN 50

R 51 QHHRILVVGCGNSAFSEGMADDGYDDVVSIDISSVVIQAMQDKYSDRPHL 100

||||||||||||||||||||||||||||||||||||||||||||||||||

S 51 QHHRILVVGCGNSAFSEGMADDGYDDVVSIDISSVVIQAMQDKYSDRPHL 100

R 101 KYLQMDVRDMSAFQTDSFDAVVDKGTLDSLLCGSNSRQNAAEMLDEVWRV 150

||||||||||||||||||||||||||||||||||||||||||||||||||

S 101 KYLQMDVRDMSAFQTDSFDAVVDKGTLDSLLCGSNSRQNAAEMLDEVWRV 150

R 151 LKDKGVYILITYGAPLYRLHLLRESCSWTIKLHVIAVDFLSVTTYAEKLA 200

||||||||||||||||||||||||||||||||||| ||||

S 151 LKDKGVYILITYGAPLYRLHLLRESCSWTIKLHVI-----------EKLA 189

R 201 CEDKSEPPIWELTNPIPLNDDGSSAEELLGNNPDVHYIYVCAKDNSLKPG 250

||||||||||||||||||||||||||||||||||||||||||||||||||

S 190 CEDKSEPPIWELTNPIPLNDDGSSAEELLGNNPDVHYIYVCAKDNSLKPG 239

R 251 LKRETSVD 258

||||||||

S 240 LKRETSVD 247

Aligned_sequences: 2

1: **Par.chr1R_long.6.106**

2: **Par.chr1S_long.6.112**

Matrix: EDNAFULL

Gap_penalty: 10.0

Extend_penalty: 0.5

Length: 777

Identity: 744/777 (95.8%)

Similarity: 744/777 (95.8%)

Gaps: 33/777 ( 4.2%)

Score: 3694.0

Green color: START CODON

Red color: STOP CODON

R 1 ATGACGATGGGGACGACGACGACGCAGGCGTACGGTGAGCCGTGGTACTG 50

||||||||||||||||||||||||||||||||||||||||||||||||||

S 1 ATGACGATGGGGACGACGACGACGCAGGCGTACGGTGAGCCGTGGTACTG 50

R 51 GGACAACCGCTACGCCAACGAATCAGGGCCGTTCGATTGGTACCAGAAGT 100

||||||||||||||||||||||||||||||||||||||||||||||||||

S 51 GGACAACCGCTACGCCAACGAATCAGGGCCGTTCGATTGGTACCAGAAGT 100

R 101 ACCAATCTTTGGCGCCGCTCATCAATCTCTACGTCCCCCGCCACTCCAAC 150

||||||||||||||||||||||||||||||||||||||||||||||||||

S 101 ACCAATCTTTGGCGCCGCTCATCAATCTCTACGTCCCCCGCCACTCCAAC 150

R 151 CAGCACCATCGCATCCTCGTCGTCGGTTGCGGCAACTCAGCGTTCAGCGA 200

||||||||||||||||||||||||||||||||||||||||||||||||||

S 151 CAGCACCATCGCATCCTCGTCGTCGGTTGCGGCAACTCAGCGTTCAGCGA 200

R 201 AGGGATGGCTGATGATGGATACGATGATGTGGTTAGTATTGACATTTCCT 250

||||||||||||||||||||||||||||||||||||||||||||||||||

S 201 AGGGATGGCTGATGATGGATACGATGATGTGGTTAGTATTGACATTTCCT 250

R 251 CTGTGGTCATCCAAGCTATGCAGGACAAGTACTCTGACCGTCCACACCTC 300

||||||||||||||||||||||||||||||||||||||||||||||||||

S 251 CTGTGGTCATCCAAGCTATGCAGGACAAGTACTCTGACCGTCCACACCTC 300

R 301 AAATATTTGCAAATGGATGTTCGAGATATGAGTGCTTTCCAAACTGATTC 350

||||||||||||||||||||||||||||||||||||||||||||||||||

S 301 AAATATTTGCAAATGGATGTTCGAGATATGAGTGCTTTCCAAACTGATTC 350

R 351 CTTTGATGCTGTTGTTGACAAAGGAACTCTAGACTCTCTGTTGTGTGGAA 400

||||||||||||||||||||||||||||||||||||||||||||||||||

S 351 CTTTGATGCTGTTGTTGACAAAGGAACTCTAGACTCTCTGTTGTGTGGAA 400

R 401 GTAATTCGCGACAAAATGCTGCTGAAATGCTTGACGAAGTTTGGAGGGTC 450

||||||||||||||||||||||||||||||||||||||||||||||||||

S 401 GTAATTCGCGACAAAATGCTGCTGAAATGCTTGACGAAGTTTGGAGGGTC 450

R 451 CTCAAGGATAAAGGAGTCTATATTCTGATCACGTATGGAGCTCCATTGTA 500

||||||||||||||||||||||||||||||||||||||||||||||||||

S 451 CTCAAGGATAAAGGAGTCTATATTCTGATCACGTATGGAGCTCCATTGTA 500

R 501 TCGTTTGCATTTGTTGAGAGAGTCATGCTCGTGGACGATAAAACTCCATG 550

||||||||||||||||||||||||||||||||||||||||||||||||||

S 501 TCGTTTGCATTTGTTGAGAGAGTCATGCTCGTGGACGATAAAACTCCATG 550

R 551 TGATAGCTGTTGATTTTCTATCTGTCACCACGTATGCAGAGAAACTTGCC 600

|||| |||||||||||||

S 551 TGAT---------------------------------AGAGAAACTTGCC 567

R 601 TGCGAAGATAAATCAGAACCTCCAATATGGGAGCTGACAAATCCTATTCC 650

||||||||||||||||||||||||||||||||||||||||||||||||||

S 568 TGCGAAGATAAATCAGAACCTCCAATATGGGAGCTGACAAATCCTATTCC 617

R 651 ATTGAATGACGATGGAAGCTCAGCGGAGGAATTGCTCGGAAACAACCCTG 700

||||||||||||||||||||||||||||||||||||||||||||||||||

S 618 ATTGAATGACGATGGAAGCTCAGCGGAGGAATTGCTCGGAAACAACCCTG 667

R 701 ATGTGCATTATATTTACGTTTGTGCAAAGGATAATTCTTTAAAGCCAGGC 750

||||||||||||||||||||||||||||||||||||||||||||||||||

S 668 ATGTGCATTATATTTACGTTTGTGCAAAGGATAATTCTTTAAAGCCAGGC 717

R 751 CTTAAGCGTGAAACATCAGTTGATTGA 777

|||||||||||||||||||||||||||

S 718 CTTAAGCGTGAAACATCAGTTGATTGA 744

**Pa46 - Kinesin-like protein KIN-12C**

Aligned_sequences: 2

1: **Par.chr1R_long.6.121**

2: **Par.chr1S_long.6.131**

Matrix: EBLOSUM62

Gap_penalty: 10.0

Extend_penalty: 0.5

Length: 2319

Identity: 539/2319 (23.2%)

Similarity: 543/2319 (23.4%)

Gaps: 1766/2319 (76.2%)

Score: 2747.0

R 1 MSKESSTVRFPARNVSKNSQSEPNENEFEASSNQIHFPPPRTPLNSIADP 50

||||||||||||||||||||||||||||||||||||||||||||||||||

S 1 MSKESSTVRFPARNVSKNSQSEPNENEFEASSNQIHFPPPRTPLNSIADP 50

R 51 AQLQKELHELDFDSQPKFEAIRSGRYSLSDRKLEAPDRAGNGGLSYGTPR 100

||||||||||||||||||||||||||||||||||||||||||||||||||

S 51 AQLQKELHELDFDSQPKFEAIRSGRYSLSDRKLEAPDRAGNGGLSYGTPR 100

R 101 VSGRGGKAHSEPNSAQSTPARNGSRASIGGTFCTGSKAPQYNGGRAGSCS 150

||||||||||||||||||||||||||||||||||||||||||||||||||

S 101 VSGRGGKAHSEPNSAQSTPARNGSRASIGGTFCTGSKAPQYNGGRAGSCS 150

R 151 RISREISVVNSEVLTQVPHFELAEDSSFWTDHNVQVLIRIRPLSNIERAS 200

||||||||||||||||||||||||||||||||||||||||||||||||||

S 151 RISREISVVNSEVLTQVPHFELAEDSSFWTDHNVQVLIRIRPLSNIERAS 200

R 201 QGHGGCLKQESAKTLVWHGHPETRFTFDHIACETISQEKLFKVAGLPMVE 250

||||||||||||||||||||||||||||||||||||||||||||||||||

S 201 QGHGGCLKQESAKTLVWHGHPETRFTFDHIACETISQEKLFKVAGLPMVE 250

R 251 NCLSGYNSCMFAYGQTGSGKTYTMMGEIYEVEGQLNEDCGITPRIFEYLF 300

||||||||||||||||||||||||||||||||||||||||||||||||||

S 251 NCLSGYNSCMFAYGQTGSGKTYTMMGEIYEVEGQLNEDCGITPRIFEYLF 300

R 301 KRIRVEEESRTEEQLKYSCKCSFLEIYNEQITDLLEPSSTNLQLREDLKK 350

||||||||||||||||||||||||||||||||||||||||||||||||||

S 301 KRIRVEEESRTEEQLKYSCKCSFLEIYNEQITDLLEPSSTNLQLREDLKK 350

R 351 GVYVENLTEYNVRDVNDVVKLLLQVGASNRKMAATHMNSESSRSHSVFTC 400

||||||||||||||||||||||||||||||||||||||||||||||||||

S 351 GVYVENLTEYNVRDVNDVVKLLLQVGASNRKMAATHMNSESSRSHSVFTC 400

R 401 IIESRWEKDSMTHFRFARLNLVDLAGSERQKSSGAEGDRLKEAANINKSL 450

||||||||||||||||||||||||||||||||||||||||||||||||||

S 401 IIESRWEKDSMTHFRFARLNLVDLAGSERQKSSGAEGDRLKEAANINKSL 450

R 451 STLGLVIMSLVDLAHGKHRHVPYRDSRLTFLLQDSLGGNSKTTIIANVSP 500

||||||||||||||||||||||||||||||||||||||||||||||||||

S 451 STLGLVIMSLVDLAHGKHRHVPYRDSRLTFLLQDSLGGNSKTTIIANVSP 500

R 501 SICSANETLSTLKFAQRAKLIQNNAKVNEDASDGRTKAFFEPLQGQLSFL 550

|||||||||||||||||||||||||||||||| |...|..:.:| ||.:

S 501 SICSANETLSTLKFAQRAKLIQNNAKVNEDAS-GDITALQQQIQ-QLKY- 547

R 551 MKHHNISWSSSSGVPSIEEPRLNNLPEEYNDSLEDKMPTDNLKLPSIRNK 600

.:..||

S 548 ----------RASCPS---------------------------------- 553

R 601 KMKCMDTILVGALRREKMADSAVQKLVAEIEDMNRLVCQSEEDAGHAKMM 650

S 554 -------------------------------------------------- 553

R 651 LRFREEKIKRLELLTDGMLSAEKYLMEENKALLEEIQLLQARFESNPELT 700

S 554 -------------------------------------------------- 553

R 701 RYSVENCRLLEQLKLYQKFYEHGERETLLAEVSELRNQLLDILQGKLPFL 750

S 554 -------------------------------------------------- 553

R 751 TENENQVGKHFLNSDTIKDLEDCRNMNSKLIREVDELQLELQKYMNSSQA 800

S 554 -------------------------------------------------- 553

R 801 ASGSVRDSFSKDTEEFRQSDKYSMVETLSMGSDSGDETASYSQEECCRGM 850

S 554 -------------------------------------------------- 553

R 851 YISSNNGKIEIQSEVKHERRYLKSGDLHKENKCIMEISEDVERKALQAKL 900

S 554 -------------------------------------------------- 553

R 901 DKMVKDLEEVRLLNSHFQEDRLLQLSHQKQTEIVCEQVEMETANTILHLQ 950

S 554 -------------------------------------------------- 553

R 951 EEVAALQFELDERLHCMIQENKVLKNTIAAKEDEIRSLSVEWEKATFELT 1000

S 554 -------------------------------------------------- 553

R 1001 RFLLDGSRSLKNASSQIESIACSFPQANVCISEDVQRAAKVCMEKEETIE 1050

S 554 -------------------------------------------------- 553

R 1051 LLQKSLEDAQKMVTEMGEKLSSLKGAAIALSELQHLDNDETKEEISFCMR 1100

S 554 -------------------------------------------------- 553

R 1101 LDEQTNMVEMLERKLIFKEIQIKEAENCANAAFLVIKWLTDQKATDKTER 1150

S 554 -------------------------------------------------- 553

R 1151 NIPISILGTPAGMASQKSSDTKVNALGQEDVITELELARLRILEYENAIE 1200

S 554 -------------------------------------------------- 553

R 1201 AFYADTEMHIVALETNISEVSDEYKELVQNLVSELHEMRKKYMELREHSE 1250

S 554 -------------------------------------------------- 553

R 1251 VSQFCTVESLSLEAHKYLKSKDIYHMILEIKNELTVANGRLKITEDFIYT 1300

S 554 -------------------------------------------------- 553

R 1301 KVNVYDCPSADKSLEDEDEWSTDSTTSSCDSSTESFASVNKLWALEGQTG 1350

S 554 -------------------------------------------------- 553

R 1351 DLKVKEGSVLQSADQDPEESKWVLKTFTDSKGATFCLKKELEMALDAFNK 1400

S 554 -------------------------------------------------- 553

R 1401 LYVRLATLISKLDIGGCSQPAGLKQLVPLFESGTESSYGCHATKKVVSDE 1450

S 554 -------------------------------------------------- 553

R 1451 KSDFASSFLTKFEEAHATIKEADVMLNALMEANENAKELTGLWKQTGEEL 1500

S 554 -------------------------------------------------- 553

R 1501 MLEKASFIEEVEHLKNSVRLKERENELLQDQSRYNLVEIAKSLSLLEECF 1550

S 554 -------------------------------------------------- 553

R 1551 MQLKSEVEDRFKVLYADTFSMGREIHCFISKSRSLLEEICAETLEKKFAI 1600

S 554 -------------------------------------------------- 553

R 1601 FVLHQCLTGELIHKIPCFNVGSGFRSSQQQEGLSITNKQQKMWSNCEDDI 1650

S 554 -------------------------------------------------- 553

R 1651 ALTSNISKDDNDQSGVTNLKAGELSLSRDSLMHENLSLKEELQRKDALLE 1700

S 554 -------------------------------------------------- 553

R 1701 GLHFDFRMLQESASNTMDIKDETEKLIKSLSQIQNELKIKTCQLDDMLFQ 1750

S 554 -------------------------------------------------- 553

R 1751 HKKLEDHLTDTERALLLSNSDLEQAKDTINTLSEQNFELKVLLNDLYRKN 1800

S 554 -------------------------------------------------- 553

R 1801 SEANEQLEEQKEVVKGLEKEILHLTSSMETKLLCQVEGIEDELRRVISER 1850

S 554 -------------------------------------------------- 553

R 1851 DGLLEEVASLNDKLEMAYAISDEHEAISIEARQESEASKMYAEQKEEEVK 1900

S 554 -------------------------------------------------- 553

R 1901 ILERSVEELECTINVLEKKVYEMNDEVERHRLIRDALELELQALRHRLLT 1950

S 554 -------------------------------------------------- 553

R 1951 VENFSENVDSENMNSEQAENLISRQLQSRLLELHEAHNKIKLLEEERAEQ 2000

S 554 -------------------------------------------------- 553

R 2001 DKEIKQCKEYISELVLHAEAQTSQYQQKYKTLEAMVCEVKADKTDSASTA 2050

S 554 -------------------------------------------------- 553

R 2051 AALEKSERSSIRTRGSSSPFRCISSLVQQMNTEKDQELSIARHRIEELEA 2100

S 554 -------------------------------------------------- 553

R 2101 LAASRQKEVCLLNTRLAAAESMTHDVIRDLLGVKLDMTNYANLIEQYQVQ 2150

S 554 -------------------------------------------------- 553

R 2151 KLVEEAHQQTEEFQEKEQEILNLRKQITDLMEERQSCISEINKKEGDIVA 2200

S 554 -------------------------------------------------- 553

R 2201 AQMTLQQLQDRDQLLSAQNEMLKADKTNLKRRVAELDEMVKTILGTPTIH 2250

S 554 -------------------------------------------------- 553

R 2251 QPIQHPHTSKPKACFLFQNNSSLKLHEIDFTKRLEQSEKHLSRVNGELAQ 2300

S 554 -------------------------------------------------- 553

R 2301 YFKSAGGGGGHPRDKRVSR 2319

S 554 ------------------- 553

Aligned_sequences: 2

1: **Par.chr1R_long.6.121**

2: **Par.chr1S_long.6.131**

Matrix: EDNAFULL

Gap_penalty: 10.0

Extend_penalty: 0.5

Length: 6975

Identity: 6934/6975 (99.4%)

Similarity: 6934/6975 (99.4%)

Gaps: 18/6975 ( 0.3%)

Score: 34521.5

Green color: START CODON

Red color: STOP CODON

R 1 ATGTCGAAAGAGAGCTCAACTGTTCGATTCCCGGCTCGAAACGTTTCGAA 50

||||||||||||||||||||||||||||||||||||||||||||||||||

S 1 ATGTCGAAAGAGAGCTCAACTGTTCGATTCCCGGCTCGAAACGTTTCGAA 50

R 51 GAACTCACAATCTGAACCGAACGAGAACGAGTTCGAAGCCTCGTCGAACC 100

||||||||||||||||||||||||||||||||||||||||||||||||||

S 51 GAACTCACAATCTGAACCGAACGAGAACGAGTTCGAAGCCTCGTCGAACC 100

R 101 AAATCCATTTTCCTCCTCCTAGAACACCTCTAAACAGCATAGCAGATCCA 150

||||||||||||||||||||||||||||||||||||||||||||||||||

S 101 AAATCCATTTTCCTCCTCCTAGAACACCTCTAAACAGCATAGCAGATCCA 150

R 151 GCTCAGCTGCAAAAGGAGCTTCACGAACTCGATTTCGATTCACAACCGAA 200

||||||||||||||||||||||||||||||||||||||||||||||||||

S 151 GCTCAGCTGCAAAAGGAGCTTCACGAACTCGATTTCGATTCACAACCGAA 200

R 201 GTTTGAAGCAATTCGATCTGGTCGGTATTCTTTATCGGATAGAAAACTTG 250

||||||||||||||||||||||||||||||||||||||||||||||||||

S 201 GTTTGAAGCAATTCGATCTGGTCGGTATTCTTTATCGGATAGAAAACTTG 250

R 251 AAGCCCCGGACAGAGCTGGAAATGGCGGTCTCAGCTATGGAACTCCTAGG 300

||||||||||||||||||||||||||||||||||||||||||||||||||

S 251 AAGCCCCGGACAGAGCTGGAAATGGCGGTCTCAGCTATGGAACTCCTAGG 300

R 301 GTTTCAGGTCGCGGAGGGAAGGCGCACTCGGAGCCAAACTCAGCGCAAAG 350

||||||||||||||||||||||||||||||||||||||||||||||||||

S 301 GTTTCAGGTCGCGGAGGGAAGGCGCACTCGGAGCCAAACTCAGCGCAAAG 350

R 351 CACTCCGGCGAGGAACGGCTCTAGAGCTTCGATTGGTGGAACATTTTGTA 400

|||||||||||||||||||||.||||||||||||||||||||||||||||

S 351 CACTCCGGCGAGGAACGGCTCGAGAGCTTCGATTGGTGGAACATTTTGTA 400

R 401 CAGGAAGCAAAGCTCCACAGTATAATGGAGGCAGAGCAGGGAGTTGTTCT 450

||||||||||||||||||||||||||||||||||||||||||||||||||

S 401 CAGGAAGCAAAGCTCCACAGTATAATGGAGGCAGAGCAGGGAGTTGTTCT 450

R 451 AGAATCTCCCGGGAGATTTCGGTGGTGAATTCCGAGGTTTTAACTCAGGT 500

||||||||||||||||||||||||||||||||||||||||||||||||||

S 451 AGAATCTCCCGGGAGATTTCGGTGGTGAATTCCGAGGTTTTAACTCAGGT 500

R 501 TCCACATTTCGAGCTTGCCGAAGATTCATCGTTCTGGACTGATCACAATG 550

||||||||||||||||||||||||||||||||||||||||||||||||||

S 501 TCCACATTTCGAGCTTGCCGAAGATTCATCGTTCTGGACTGATCACAATG 550

R 551 TGCAGGTGCTGATTCGAATTCGTCCATTGAGTAATATAGAGAGGGCTTCG 600

||||||||||||||||||||||||||||||||||||||||||||||||||

S 551 TGCAGGTGCTGATTCGAATTCGTCCATTGAGTAATATAGAGAGGGCTTCG 600

R 601 CAAGGGCACGGTGGGTGTTTGAAGCAGGAAAGCGCCAAGACTTTGGTGTG 650

||||||||||||||||||||||||||||||||||||||||||||||||||

S 601 CAAGGGCACGGTGGGTGTTTGAAGCAGGAAAGCGCCAAGACTTTGGTGTG 650

R 651 GCATGGTCATCCTGAAACCAGATTCACATTTGATCATATTGCATGCGAGA 700

||||||||||||||||||||||||||||||||||||||||||||||||||

S 651 GCATGGTCATCCTGAAACCAGATTCACATTTGATCATATTGCATGCGAGA 700

R 701 CAATATCACAGGAAAAGCTGTTCAAAGTTGCTGGGTTGCCCATGGTAGAG 750

||||||||||||||||||||||||||||||||||||||||||||||||||

S 701 CAATATCACAGGAAAAGCTGTTCAAAGTTGCTGGGTTGCCCATGGTAGAG 750

R 751 AATTGCTTGTCTGGTTATAATAGCTGCATGTTTGCTTACGGTCAGACGGG 800

||||||||||||||||||||||||||||||||||||||||||||||||||

S 751 AATTGCTTGTCTGGTTATAATAGCTGCATGTTTGCTTACGGTCAGACGGG 800

R 801 CAGTGGCAAAACGTACACAATGATGGGTGAAATATATGAGGTTGAAGGGC 850

||||||||||||||||||||||||||||||||||||||||||||||||||

S 801 CAGTGGCAAAACGTACACAATGATGGGTGAAATATATGAGGTTGAAGGGC 850

R 851 AGCTCAATGAAGATTGTGGGATTACTCCACGCATTTTCGAATATTTGTTT 900

||||||||||||||||||||||||||||||||||||||||||||||||||

S 851 AGCTCAATGAAGATTGTGGGATTACTCCACGCATTTTCGAATATTTGTTT 900

R 901 AAGAGGATCAGAGTGGAAGAGGAGAGCAGGACGGAGGAACAGTTGAAGTA 950

||||||||||||||||||||||||||||||||||||||||||||||||||

S 901 AAGAGGATCAGAGTGGAAGAGGAGAGCAGGACGGAGGAACAGTTGAAGTA 950

R 951 CAGCTGCAAATGTTCCTTTCTTGAGATTTACAATGAGCAGATAACAGATC 1000

||||||||||||||||||||||||||||||||||||||||||||||||||

S 951 CAGCTGCAAATGTTCCTTTCTTGAGATTTACAATGAGCAGATAACAGATC 1000

R 1001 TCTTGGAGCCTTCATCAACTAATCTACAACTCAGGGAGGACCTGAAGAAA 1050

||||||||||||||||||||||||||||||||||||||||||||||||||

S 1001 TCTTGGAGCCTTCATCAACTAATCTACAACTCAGGGAGGACCTGAAGAAA 1050

R 1051 GGGGTATATGTTGAAAACCTTACAGAATATAATGTGAGGGATGTTAATGA 1100

||||||||||||||||||||||||||||||||||||||||||||||||||

S 1051 GGGGTATATGTTGAAAACCTTACAGAATATAATGTGAGGGATGTTAATGA 1100

R 1101 TGTTGTCAAGCTTCTGTTACAGGTGGGTGCTTCAAACAGAAAAATGGCGG 1150

||||||||||||||||||||||||||||||||||||||||||||||||||

S 1101 TGTTGTCAAGCTTCTGTTACAGGTGGGTGCTTCAAACAGAAAAATGGCGG 1150

R 1151 CAACACACATGAACAGTGAGAGCAGCCGGTCCCACAGTGTTTTCACTTGT 1200

||||||||||||||||||||||||||||||||||||||||||||||||||

S 1151 CAACACACATGAACAGTGAGAGCAGCCGGTCCCACAGTGTTTTCACTTGT 1200

R 1201 ATCATTGAAAGCCGTTGGGAAAAAGATTCCATGACCCATTTTAGGTTTGC 1250

||||||||||||||||||||||||||||||||||||||||||||||||||

S 1201 ATCATTGAAAGCCGTTGGGAAAAAGATTCCATGACCCATTTTAGGTTTGC 1250

R 1251 AAGGTTGAACTTAGTAGATTTAGCTGGTTCAGAAAGGCAGAAAAGCTCTG 1300

||||||||||||||||||||||||||||||||||||||||||||||||||

S 1251 AAGGTTGAACTTAGTAGATTTAGCTGGTTCAGAAAGGCAGAAAAGCTCTG 1300

R 1301 GTGCAGAGGGAGATCGTTTGAAAGAAGCAGCAAATATTAACAAATCATTA 1350

||||||||||||||||||||||||||||||||||||||||||||||||||

S 1301 GTGCAGAGGGAGATCGTTTGAAAGAAGCAGCAAATATTAACAAATCATTA 1350

R 1351 TCAACTCTTGGGTTGGTGATAATGTCTTTAGTTGATTTAGCACATGGGAA 1400

||||||||||||||||||||||||||||||||||||||||||||||||||

S 1351 TCAACTCTTGGGTTGGTGATAATGTCTTTAGTTGATTTAGCACATGGGAA 1400

R 1401 ACATAGACATGTTCCTTATAGAGATTCAAGACTTACGTTTCTGCTTCAGG 1450

||||||||||||||||||||||||||||||||||||||||||||||||||

S 1401 ACATAGACATGTTCCTTATAGAGATTCAAGACTTACGTTTCTGCTTCAGG 1450

R 1451 ATTCTCTGGGTGGGAACTCAAAAACAACTATAATTGCCAATGTCAGCCCA 1500

||||||||||||||||||||||||||||||||||||||||||||||||||

S 1451 ATTCTCTGGGTGGGAACTCAAAAACAACTATAATTGCCAATGTCAGCCCA 1500

R 1501 TCTATTTGCTCTGCAAATGAGACACTCAGCACTTTGAAGTTTGCCCAGCG 1550

||||||||||||||||||||||||||||||||||||||||||||||||||

S 1501 TCTATTTGCTCTGCAAATGAGACACTCAGCACTTTGAAGTTTGCCCAGCG 1550

R 1551 TGCCAAACTTATCCAGAACAATGCTAAAGTGAATGAAGATGCTTCGGATG 1600

|||||||||||||||||||||||||||||||||||||||||||||||.||

S 1551 TGCCAAACTTATCCAGAACAATGCTAAAGTGAATGAAGATGCTTCGGGTG 1600

R 1601 GTAGAACTAAAGCATTT---------TTCGAAC-CCT----TACAGGGCC 1636

..|.|||| |||.|| .||.||| .|| |||||||||

S 1601 ACATAACT---GCACTTCAGCAACAAATCCAACAGCTAAAGTACAGGGCC 1647

R 1637 AGTTGTCCTTCCTAATGAAGCACCATAATATCTCATGGTCTTCATCAAGT 1686

||||||||||||||||||||||||||||||||||||||||||||||||||

S 1648 AGTTGTCCTTCCTAATGAAGCACCATAATATCTCATGGTCTTCATCAAGT 1697

R 1687 GGCGTGCCAAGTATTGAAGAACCTAGACTCAATAATTTGCCTGAGGAATA 1736

||||||||.|||||||||||||||||||||||||||||||||||||||||

S 1698 GGCGTGCCGAGTATTGAAGAACCTAGACTCAATAATTTGCCTGAGGAATA 1747

R 1737 CAATGACTCTCTGGAAGATAAAATGCCAACTGATAATCTTAAGTTACCAA 1786

||||||||||||||||||||||||||||||||||||||||||||||||||

S 1748 CAATGACTCTCTGGAAGATAAAATGCCAACTGATAATCTTAAGTTACCAA 1797

R 1787 GTATCAGAAACAAGAAGATGAAATGCATGGATACCATCTTAGTTGGTGCT 1836

||||||||||||||||||||||||||||||||||||||||||||||||||

S 1798 GTATCAGAAACAAGAAGATGAAATGCATGGATACCATCTTAGTTGGTGCT 1847

R 1837 TTAAGGAGGGAAAAGATGGCAGACAGTGCAGTCCAGAAGTTGGTGGCTGA 1886

||||||||||||||||||||||||||||||||||||||||||||||||||

S 1848 TTAAGGAGGGAAAAGATGGCAGACAGTGCAGTCCAGAAGTTGGTGGCTGA 1897

R 1887 AATTGAAGACATGAACCGCTTGGTTTGCCAAAGTGAGGAGGATGCTGGAC 1936

||||||||||||||||||||||||||||||||||||||||||||||||||

S 1898 AATTGAAGACATGAACCGCTTGGTTTGCCAAAGTGAGGAGGATGCTGGAC 1947

R 1937 ATGCTAAAATGATGCTAAGGTTTCGTGAGGAAAAAATTAAACGACTTGAA 1986

||||||||||||||||||||||||||||||||||||||||||||||||||

S 1948 ATGCTAAAATGATGCTAAGGTTTCGTGAGGAAAAAATTAAACGACTTGAA 1997

R 1987 TTGTTGACGGATGGAATGCTGTCGGCTGAGAAGTATCTCATGGAGGAGAA 2036

||||||||||||||||||||||||||||||||||||||||||||||||||

S 1998 TTGTTGACGGATGGAATGCTGTCGGCTGAGAAGTATCTCATGGAGGAGAA 2047

R 2037 CAAGGCTTTACTTGAAGAGATTCAGCTGCTGCAAGCAAGATTTGAAAGCA 2086

||||||||||||||||||||||||||||||||||||||||||||||||||

S 2048 CAAGGCTTTACTTGAAGAGATTCAGCTGCTGCAAGCAAGATTTGAAAGCA 2097

R 2087 ATCCAGAATTGACCAGATATTCTGTGGAGAACTGTAGACTTCTCGAGCAA 2136

||||||||||||||||||||||||||||||||||||||||||||||||||

S 2098 ATCCAGAATTGACCAGATATTCTGTGGAGAACTGTAGACTTCTCGAGCAA 2147

R 2137 CTTAAATTGTACCAAAAGTTTTATGAACATGGAGAGAGAGAAACATTGCT 2186

||||||||||||||||||||||||||||||||||||||||||||||||||

S 2148 CTTAAATTGTACCAAAAGTTTTATGAACATGGAGAGAGAGAAACATTGCT 2197

R 2187 AGCTGAAGTATCAGAACTACGCAATCAGCTTCTGGATATACTTCAAGGAA 2236

||||||||||||||||||||||||||||||||||||||||||||||||||

S 2198 AGCTGAAGTATCAGAACTACGCAATCAGCTTCTGGATATACTTCAAGGAA 2247

R 2237 AACTTCCATTCTTAACAGAAAATGAAAATCAGGTCGGAAAGCACTTTTTG 2286

||||||||||||||||||||||||||||||||||||||||||||||||||

S 2248 AACTTCCATTCTTAACAGAAAATGAAAATCAGGTCGGAAAGCACTTTTTG 2297

R 2287 AATAGTGATACCATAAAGGATTTGGAAGATTGCAGGAACATGAATTCTAA 2336

||||||||||||||||||||||||||||||||||||||||||||||||||

S 2298 AATAGTGATACCATAAAGGATTTGGAAGATTGCAGGAACATGAATTCTAA 2347

R 2337 ATTGATCAGGGAAGTAGATGAACTACAACTAGAATTGCAAAAGTATATGA 2386

||||||||||||||||||||||||||||||||||||||||||||||||||

S 2348 ATTGATCAGGGAAGTAGATGAACTACAACTAGAATTGCAAAAGTATATGA 2397

R 2387 ACTCCAGTCAAGCTGCCTCCGGTTCTGTAAGAGATTCTTTCTCCAAGGAT 2436

||||||||||||||||||||||||||||||||||||||||||||||||||

S 2398 ACTCCAGTCAAGCTGCCTCCGGTTCTGTAAGAGATTCTTTCTCCAAGGAT 2447

R 2437 ACTGAGGAATTCAGGCAATCAGATAAGTATTCGATGGTTGAAACCCTATC 2486

||||||||||||||||||||||||||||||||||||||||||||||||||

S 2448 ACTGAGGAATTCAGGCAATCAGATAAGTATTCGATGGTTGAAACCCTATC 2497

R 2487 TATGGGGAGTGACTCTGGAGATGAAACGGCATCTTACTCCCAGGAGGAAT 2536

||||||||||||||||||||||||||||||||||||||||||||||||||

S 2498 TATGGGGAGTGACTCTGGAGATGAAACGGCATCTTACTCCCAGGAGGAAT 2547

R 2537 GCTGCAGAGGCATGTACATATCAAGCAACAACGGCAAAATAGAAATCCAA 2586

|||||||||||||||||||||||||||||||.||||||||||||||||||

S 2548 GCTGCAGAGGCATGTACATATCAAGCAACAATGGCAAAATAGAAATCCAA 2597

R 2587 TCGGAGGTGAAACATGAACGTCGTTATCTGAAATCAGGTGATTTACATAA 2636

||||||||||||||||||||||||||||||||||||||||||||||||||

S 2598 TCGGAGGTGAAACATGAACGTCGTTATCTGAAATCAGGTGATTTACATAA 2647

R 2637 GGAAAACAAATGTATTATGGAGATCAGTGAAGACGTTGAGAGGAAGGCTT 2686

||||||||||||||||||||||||||||||||||||||||||||||||||

S 2648 GGAAAACAAATGTATTATGGAGATCAGTGAAGACGTTGAGAGGAAGGCTT 2697

R 2687 TGCAAGCTAAATTGGACAAAATGGTTAAGGACCTGGAGGAGGTCAGATTA 2736

||||||||||||||||||||||||||||||||||||||||||||||||||

S 2698 TGCAAGCTAAATTGGACAAAATGGTTAAGGACCTGGAGGAGGTCAGATTA 2747

R 2737 CTCAATAGCCACTTTCAGGAGGATCGGCTATTACAGTTGTCTCACCAGAA 2786

||||||||||||||||||||||||||||||||||||||||||||||||||

S 2748 CTCAATAGCCACTTTCAGGAGGATCGGCTATTACAGTTGTCTCACCAGAA 2797

R 2787 ACAGACTGAAATAGTCTGTGAACAGGTTGAGATGGAGACAGCAAACACAA 2836

||||||||||||||||||||||||||||||||||||||||||||||||||

S 2798 ACAGACTGAAATAGTCTGTGAACAGGTTGAGATGGAGACAGCAAACACAA 2847

R 2837 TTCTTCATTTACAGGAAGAGGTTGCTGCCCTTCAGTTTGAACTTGATGAG 2886

||||||||||||||||||||||||||||||||||||||||||||||||||

S 2848 TTCTTCATTTACAGGAAGAGGTTGCTGCCCTTCAGTTTGAACTTGATGAG 2897

R 2887 AGATTACACTGCATGATTCAGGAAAATAAGGTACTGAAAAACACCATTGC 2936

||||||||||||||||||||||||||||||||||||||||||||||||||

S 2898 AGATTACACTGCATGATTCAGGAAAATAAGGTACTGAAAAACACCATTGC 2947

R 2937 AGCTAAAGAGGATGAGATAAGGTCACTGAGTGTGGAGTGGGAAAAGGCAA 2986

||||||||||||||||||||||||||||||||||||||||||||||||||

S 2948 AGCTAAAGAGGATGAGATAAGGTCACTGAGTGTGGAGTGGGAAAAGGCAA 2997

R 2987 CCTTTGAACTAACAAGATTCCTCCTAGATGGTTCTAGATCCCTCAAAAAT 3036

||||||||||||||||||||||||||||||||||||||||||||||||||

S 2998 CCTTTGAACTAACAAGATTCCTCCTAGATGGTTCTAGATCCCTCAAAAAT 3047

R 3037 GCCTCCAGCCAAATAGAAAGTATTGCTTGTTCATTTCCTCAAGCTAATGT 3086

||||||||||||||||||||||||||||||||||||||||||||||||||

S 3048 GCCTCCAGCCAAATAGAAAGTATTGCTTGTTCATTTCCTCAAGCTAATGT 3097

R 3087 TTGTATTAGTGAAGATGTCCAGAGGGCTGCCAAAGTTTGCATGGAAAAGG 3136

||||||||||||||||||||||||||||||||||||||||||||||||||

S 3098 TTGTATTAGTGAAGATGTCCAGAGGGCTGCCAAAGTTTGCATGGAAAAGG 3147

R 3137 AAGAAACAATTGAACTACTACAAAAGAGTTTGGAAGATGCGCAAAAGATG 3186

||||||||||||||||||||||||||||||||||||||||||||||||||

S 3148 AAGAAACAATTGAACTACTACAAAAGAGTTTGGAAGATGCGCAAAAGATG 3197

R 3187 GTAACAGAAATGGGAGAGAAGTTAAGTTCCTTGAAGGGAGCAGCAATTGC 3236

||||||||||||||||||||||||||||||||||||||||||||||||||

S 3198 GTAACAGAAATGGGAGAGAAGTTAAGTTCCTTGAAGGGAGCAGCAATTGC 3247

R 3237 TTTAAGTGAACTCCAACATCTGGATAATGATGAAACCAAAGAGGAAATTT 3286

||||||||||||||||||||||||||||||||||||||||||||||||||

S 3248 TTTAAGTGAACTCCAACATCTGGATAATGATGAAACCAAAGAGGAAATTT 3297

R 3287 CCTTCTGCATGCGATTGGATGAGCAGACCAACATGGTAGAGATGCTAGAG 3336

||||||||||||||||||||||||||||||||||||||||||||||||||

S 3298 CCTTCTGCATGCGATTGGATGAGCAGACCAACATGGTAGAGATGCTAGAG 3347

R 3337 AGGAAACTTATATTCAAGGAAATTCAGATCAAAGAAGCAGAAAATTGTGC 3386

||||||||||||||||||||||||||||||||||||||||||||||||||

S 3348 AGGAAACTTATATTCAAGGAAATTCAGATCAAAGAAGCAGAAAATTGTGC 3397

R 3387 CAATGCTGCATTCCTGGTAATAAAATGGCTTACGGATCAGAAGGCAACAG 3436

||||||||||||||||||||||||||||||||||||||||||||||||||

S 3398 CAATGCTGCATTCCTGGTAATAAAATGGCTTACGGATCAGAAGGCAACAG 3447

R 3437 ACAAGACAGAGAGAAACATTCCCATCTCAATACTAGGTACACCAGCCGGA 3486

||||||||||||||||||||||||||||||||||||||||||||||||||

S 3448 ACAAGACAGAGAGAAACATTCCCATCTCAATACTAGGTACACCAGCCGGA 3497

R 3487 ATGGCCAGCCAGAAAAGTTCTGACACAAAAGTAAATGCTTTAGGACAAGA 3536

||||||||||||||||||||||||||||||||||||||||||||||||||

S 3498 ATGGCCAGCCAGAAAAGTTCTGACACAAAAGTAAATGCTTTAGGACAAGA 3547

R 3537 AGATGTCATTACTGAACTTGAGTTGGCCAGGTTAAGAATATTGGAGTATG 3586

||||||||||||||||||||||||||||||||||||||||||||||||||

S 3548 AGATGTCATTACTGAACTTGAGTTGGCCAGGTTAAGAATATTGGAGTATG 3597

R 3587 AGAATGCTATCGAAGCATTTTATGCAGATACAGAAATGCACATAGTGGCC 3636

||||||||||||||||||||||||||||||||||||||||||||||||||

S 3598 AGAATGCTATCGAAGCATTTTATGCAGATACAGAAATGCACATAGTGGCC 3647

R 3637 CTTGAGACCAACATCAGTGAAGTTTCTGATGAATACAAGGAGTTGGTTCA 3686

||||||||||||||||||||||||||||||||||||||||||||||||||

S 3648 CTTGAGACCAACATCAGTGAAGTTTCTGATGAATACAAGGAGTTGGTTCA 3697

R 3687 AAACTTGGTAAGTGAACTTCATGAAATGAGGAAGAAATATATGGAGTTAA 3736

||||||||||||||||||||||||||||||||||||||||||||||||||

S 3698 AAACTTGGTAAGTGAACTTCATGAAATGAGGAAGAAATATATGGAGTTAA 3747

R 3737 GAGAGCATTCCGAAGTTTCTCAGTTTTGTACAGTTGAGTCCCTATCATTA 3786

|||||||||||.||||||||||||||||||||||||||||||||||||||

S 3748 GAGAGCATTCCAAAGTTTCTCAGTTTTGTACAGTTGAGTCCCTATCATTA 3797

R 3787 GAAGCACACAAGTATCTGAAGTCTAAAGATATTTATCACATGATTCTTGA 3836

||||||||||||||||||||||||||||||||||||||||||||||||||

S 3798 GAAGCACACAAGTATCTGAAGTCTAAAGATATTTATCACATGATTCTTGA 3847

R 3837 AATAAAAAATGAGCTCACTGTAGCAAATGGTAGATTGAAAATTACTGAAG 3886

||||||||||||||||||||||||||||||||||||||||||||||||||

S 3848 AATAAAAAATGAGCTCACTGTAGCAAATGGTAGATTGAAAATTACTGAAG 3897

R 3887 ATTTCATTTACACAAAAGTAAATGTGTATGATTGCCCTTCAGCAGACAAA 3936

||||||||||||||||||||||||||||||||||||||||||||||||||

S 3898 ATTTCATTTACACAAAAGTAAATGTGTATGATTGCCCTTCAGCAGACAAA 3947

R 3937 AGTTTAGAAGATGAAGATGAATGGAGTACTGATAGTACTACGTCAAGCTG 3986

||||||||||||||||||||||||||||||||||||||||||||||||||

S 3948 AGTTTAGAAGATGAAGATGAATGGAGTACTGATAGTACTACGTCAAGCTG 3997

R 3987 TGATTCTTCAACCGAAAGTTTTGCTTCTGTAAACAAATTGTGGGCACTAG 4036

||||||||||||||||||||||||||||||||||||||||||||||||||

S 3998 TGATTCTTCAACCGAAAGTTTTGCTTCTGTAAACAAATTGTGGGCACTAG 4047

R 4037 AAGGGCAAACGGGGGACCTGAAAGTTAAAGAAGGCTCAGTACTTCAGTCT 4086

||||||||||||||||||||||||||||||||||||||||||||||||||

S 4048 AAGGGCAAACGGGGGACCTGAAAGTTAAAGAAGGCTCAGTACTTCAGTCT 4097

R 4087 GCTGATCAAGATCCAGAAGAGTCAAAGTGGGTCTTAAAAACTTTCACGGA 4136

||||||||||||||||||||||||||||||||||||||||||||||||||

S 4098 GCTGATCAAGATCCAGAAGAGTCAAAGTGGGTCTTAAAAACTTTCACGGA 4147

R 4137 CTCTAAAGGAGCAACATTCTGCCTGAAAAAGGAATTAGAGATGGCACTAG 4186

||||||||||||||||||||||||||||||||||||||||||||||||||

S 4148 CTCTAAAGGAGCAACATTCTGCCTGAAAAAGGAATTAGAGATGGCACTAG 4197

R 4187 ATGCTTTCAACAAACTATATGTTAGGCTAGCCACACTCATTAGCAAGTTG 4236

||||||||||||||||||||||||||||||||||||||||||||.|||||

S 4198 ATGCTTTCAACAAACTATATGTTAGGCTAGCCACACTCATTAGCGAGTTG 4247

R 4237 GACATTGGAGGTTGTTCTCAACCAGCAGGGCTGAAACAACTTGTTCCATT 4286

||||||||||||||||||||||||||.|||||||||||||||||||||||

S 4248 GACATTGGAGGTTGTTCTCAACCAGCTGGGCTGAAACAACTTGTTCCATT 4297

R 4287 GTTTGAGTCAGGGACGGAGAGTTCTTATGGTTGTCATGCTACAAAAAAGG 4336

||||||||||||||||||||||||||||||||||||||||||||||||||

S 4298 GTTTGAGTCAGGGACGGAGAGTTCTTATGGTTGTCATGCTACAAAAAAGG 4347

R 4337 TAGTTTCTGATGAGAAGAGTGATTTTGCTAGTAGCTTCTTAACCAAATTT 4386

||||||||||||||||||||||||||||||||||||||||||||||||||

S 4348 TAGTTTCTGATGAGAAGAGTGATTTTGCTAGTAGCTTCTTAACCAAATTT 4397

R 4387 GAAGAAGCGCATGCAACAATAAAAGAAGCTGATGTTATGTTAAATGCCTT 4436

||||||||||||||||||||||||||||||||||||||||||||||||||

S 4398 GAAGAAGCGCATGCAACAATAAAAGAAGCTGATGTTATGTTAAATGCCTT 4447

R 4437 GATGGAAGCAAATGAAAATGCAAAAGAATTGACTGGTCTGTGGAAACAAA 4486

||||||||||||||||||||||||||||||||||||||||||||||||||

S 4448 GATGGAAGCAAATGAAAATGCAAAAGAATTGACTGGTCTGTGGAAACAAA 4497

R 4487 CAGGTGAGGAACTGATGTTAGAGAAAGCAAGCTTTATTGAAGAAGTTGAA 4536

||||||||||||||||||||||||||||||||||||||||||||||||||

S 4498 CAGGTGAGGAACTGATGTTAGAGAAAGCAAGCTTTATTGAAGAAGTTGAA 4547

R 4537 CATCTCAAAAATTCAGTACGTTTGAAAGAAAGAGAGAATGAACTACTGCA 4586

|||||.||||||||||||||||||||||||||||||||||||||||||||

S 4548 CATCTAAAAAATTCAGTACGTTTGAAAGAAAGAGAGAATGAACTACTGCA 4597

R 4587 GGATCAATCTCGTTATAATTTGGTGGAGATAGCAAAATCATTGTCTTTGC 4636

||||||||||||||||||||||||||||||||||||||||||||||||||

S 4598 GGATCAATCTCGTTATAATTTGGTGGAGATAGCAAAATCATTGTCTTTGC 4647

R 4637 TTGAAGAGTGTTTTATGCAATTAAAAAGTGAAGTGGAGGACAGGTTCAAA 4686

||||||||||||||||||||||||||||||||||||||||||||||||||

S 4648 TTGAAGAGTGTTTTATGCAATTAAAAAGTGAAGTGGAGGACAGGTTCAAA 4697

R 4687 GTATTATATGCTGACACTTTTTCTATGGGAAGGGAGATACACTGCTTCAT 4736

||||||||||||||||||||||||||||||||||||||||||||||||||

S 4698 GTATTATATGCTGACACTTTTTCTATGGGAAGGGAGATACACTGCTTCAT 4747

R 4737 TAGCAAATCAAGATCATTACTAGAAGAAATATGCGCTGAGACATTGGAGA 4786

||||||||||||||||||||||||||||||||||||||||||||||||||

S 4748 TAGCAAATCAAGATCATTACTAGAAGAAATATGCGCTGAGACATTGGAGA 4797

R 4787 AAAAATTTGCCATATTTGTCCTTCATCAGTGTCTCACAGGAGAGCTGATC 4836

||||||||||||||||||||||||||||||||||||||||||||||||||

S 4798 AAAAATTTGCCATATTTGTCCTTCATCAGTGTCTCACAGGAGAGCTGATC 4847

R 4837 CACAAAATACCATGCTTTAACGTTGGAAGTGGTTTCCGCTCCAGCCAACA 4886

||||||||||||||||||||||||||||||||||||||||||||||||||

S 4848 CACAAAATACCATGCTTTAACGTTGGAAGTGGTTTCCGCTCCAGCCAACA 4897

R 4887 GCAAGAAGGCCTTTCAATTACAAACAAACAACAAAAAATGTGGTCAAATT 4936

||||||||||||||||||||||||||||||||||||||||||||||||||

S 4898 GCAAGAAGGCCTTTCAATTACAAACAAACAACAAAAAATGTGGTCAAATT 4947

R 4937 GTGAGGATGACATTGCACTTACTAGTAACATCTCCAAAGACGATAATGAT 4986

||||||||||||||||||||||||||||||||||||||||||||||||||

S 4948 GTGAGGATGACATTGCACTTACTAGTAACATCTCCAAAGACGATAATGAT 4997

R 4987 CAAAGTGGAGTCACAAATTTGAAAGCTGGTGAGCTCAGTTTGTCCCGTGA 5036

||||||||||||||||||||||||||||||||||||||||||||||||||

S 4998 CAAAGTGGAGTCACAAATTTGAAAGCTGGTGAGCTCAGTTTGTCCCGTGA 5047

R 5037 TAGTTTGATGCACGAGAATTTATCACTGAAGGAAGAACTGCAACGGAAAG 5086

||.|||||||||||||||||||||||||||||||||||||||||||||||

S 5048 TATTTTGATGCACGAGAATTTATCACTGAAGGAAGAACTGCAACGGAAAG 5097

R 5087 ATGCTCTACTGGAGGGCTTGCATTTTGATTTTAGAATGTTGCAGGAATCA 5136

||||||||||||||||||||||||||||||||||||||||||||||||||

S 5098 ATGCTCTACTGGAGGGCTTGCATTTTGATTTTAGAATGTTGCAGGAATCA 5147

R 5137 GCATCCAACACAATGGATATAAAGGATGAAACTGAAAAGCTGATAAAATC 5186

||||||||||||||||||||||||||||||||||||||||||||||||||

S 5148 GCATCCAACACAATGGATATAAAGGATGAAACTGAAAAGCTGATAAAATC 5197

R 5187 TTTGAGCCAAATTCAGAATGAACTAAAAATAAAAACATGCCAGCTTGATG 5236

||||||||||||||||||||||||||||||||||||||||||||||||||

S 5198 TTTGAGCCAAATTCAGAATGAACTAAAAATAAAAACATGCCAGCTTGATG 5247

R 5237 ACATGCTGTTTCAACATAAAAAGCTCGAGGATCATCTTACTGATACTGAA 5286

||||||||||||||||||||||||||||||||||||||||||||||||||

S 5248 ACATGCTGTTTCAACATAAAAAGCTCGAGGATCATCTTACTGATACTGAA 5297

R 5287 AGGGCTCTGCTTTTATCAAATTCTGATCTTGAGCAGGCCAAAGATACAAT 5336

||||||||||||||||||||||||.|||||||||.|||||||||||||||

S 5298 AGGGCTCTGCTTTTATCAAATTCTAATCTTGAGCGGGCCAAAGATACAAT 5347

R 5337 CAATACTCTTTCAGAGCAAAATTTTGAGTTGAAAGTGCTTCTAAATGATC 5386

||||||||||||||||||||||||||||||||||||||||||||||||||

S 5348 CAATACTCTTTCAGAGCAAAATTTTGAGTTGAAAGTGCTTCTAAATGATC 5397

R 5387 TCTATCGCAAAAATTCTGAAGCCAATGAACAACTGGAAGAGCAGAAGGAA 5436

||||||||||||||||||||||||||||||||||||||||||||||||||

S 5398 TCTATCGCAAAAATTCTGAAGCCAATGAACAACTGGAAGAGCAGAAGGAA 5447

R 5437 GTGGTGAAAGGTTTGGAAAAAGAAATTCTTCATTTGACTTCTTCAATGGA 5486

||||||||||||||||||||||||||||||||||||||||||||||||||

S 5448 GTGGTGAAAGGTTTGGAAAAAGAAATTCTTCATTTGACTTCTTCAATGGA 5497

R 5487 AACAAAATTACTCTGTCAAGTTGAAGGTATCGAGGATGAGTTGAGAAGGG 5536

||||||||||||||||||||||||||||||||||||||||||||||||||

S 5498 AACAAAATTACTCTGTCAAGTTGAAGGTATCGAGGATGAGTTGAGAAGGG 5547

R 5537 TCATCAGCGAGAGAGACGGACTTCTTGAAGAAGTTGCGTCCCTGAACGAT 5586

||||||||||||||||||||||||||||||||||||||||||||||||||

S 5548 TCATCAGCGAGAGAGACGGACTTCTTGAAGAAGTTGCGTCCCTGAACGAT 5597

R 5587 AAACTTGAGATGGCATATGCAATATCTGATGAACATGAGGCTATCTCTAT 5636

||||||||||||||||||||||||||||||||||||||||||||||||||

S 5598 AAACTTGAGATGGCATATGCAATATCTGATGAACATGAGGCTATCTCTAT 5647

R 5637 TGAAGCTCGCCAGGAATCTGAGGCAAGTAAGATGTATGCCGAACAAAAGG 5686

||||||||||||||||||||||||||||||||||||||||||||||||||

S 5648 TGAAGCTCGCCAGGAATCTGAGGCAAGTAAGATGTATGCCGAACAAAAGG 5697

R 5687 AAGAGGAGGTTAAAATATTAGAACGCTCTGTTGAGGAGCTGGAATGTACC 5736

||||||||||||||||||||||||||||||||||||||||||||||||||

S 5698 AAGAGGAGGTTAAAATATTAGAACGCTCTGTTGAGGAGCTGGAATGTACC 5747

R 5737 ATCAATGTACTGGAAAAGAAGGTATATGAGATGAATGACGAGGTAGAAAG 5786

||||||||||||||||||||||||||||||||||||||||||||||||||

S 5748 ATCAATGTACTGGAAAAGAAGGTATATGAGATGAATGACGAGGTAGAAAG 5797

R 5787 GCATCGGTTGATCAGAGATGCACTAGAGCTGGAGCTACAAGCTTTAAGAC 5836

||||||||||||||||||||||||||||||||||||||||||||||||||

S 5798 GCATCGGTTGATCAGAGATGCACTAGAGCTGGAGCTACAAGCTTTAAGAC 5847

R 5837 ACAGACTGTTAACAGTTGAAAATTTCAGTGAAAATGTGGACTCAGAAAAC 5886

||||||||||||||||||||||||||||||||||||||||||||||||||

S 5848 ACAGACTGTTAACAGTTGAAAATTTCAGTGAAAATGTGGACTCAGAAAAC 5897

R 5887 ATGAATTCTGAACAAGCTGAAAATCTGATTTCTAGGCAACTGCAGAGTAG 5936

||||||||||||||||||||||||||||||||||||||||||||||||||

S 5898 ATGAATTCTGAACAAGCTGAAAATCTGATTTCTAGGCAACTGCAGAGTAG 5947

R 5937 GCTACTGGAACTTCATGAGGCTCATAATAAGATAAAGCTTCTTGAAGAGG 5986

||||||||||||||||||||||||||||||||||||||||||||||||||

S 5948 GCTACTGGAACTTCATGAGGCTCATAATAAGATAAAGCTTCTTGAAGAGG 5997

R 5987 AAAGAGCAGAACAGGATAAAGAGATCAAACAATGCAAAGAATACATCTCT 6036

|||||.||||||||||||||||||||||||||||||||||||||||||||

S 5998 AAAGAACAGAACAGGATAAAGAGATCAAACAATGCAAAGAATACATCTCT 6047

R 6037 GAACTCGTGTTGCATGCTGAAGCCCAGACGTCACAGTACCAACAGAAGTA 6086

||||||||||||||||||||||||||||||||||||||||||||||||||

S 6048 GAACTCGTGTTGCATGCTGAAGCCCAGACGTCACAGTACCAACAGAAGTA 6097

R 6087 CAAGACTCTTGAGGCCATGGTTTGTGAAGTAAAAGCAGATAAGACAGATT 6136

||||||||||||||||||||||||||||||||||||||||||||||||||

S 6098 CAAGACTCTTGAGGCCATGGTTTGTGAAGTAAAAGCAGATAAGACAGATT 6147

R 6137 CAGCATCAACAGCTGCGGCATTAGAAAAATCTGAGAGGAGCTCAATCAGG 6186

||||||||||||||||||||||||||||||||||||||||||||||||||

S 6148 CAGCATCAACAGCTGCGGCATTAGAAAAATCTGAGAGGAGCTCAATCAGG 6197

R 6187 ACAAGGGGCTCCAGCTCACCATTCAGATGCATTTCAAGTTTGGTTCAGCA 6236

||||||||||||||||||||||||||||||||||||||||||||||||||

S 6198 ACAAGGGGCTCCAGCTCACCATTCAGATGCATTTCAAGTTTGGTTCAGCA 6247

R 6237 GATGAATACGGAGAAGGATCAGGAATTGTCTATAGCAAGGCATCGTATTG 6286

||||||||||||||||||||||||||||||||||||||||||||||||||

S 6248 GATGAATACGGAGAAGGATCAGGAATTGTCTATAGCAAGGCATCGTATTG 6297

R 6287 AGGAACTAGAGGCACTTGCAGCCAGTCGGCAGAAGGA-GGTATGTTTGCT 6335

||||||||||||||||||||||||||||||||||||| ||||||||||||

S 6298 AGGAACTAGAGGCACTTGCAGCCAGTCGGCAGAAGGAGGGTATGTTTGCT 6347

R 6336 GAACACTAGGCTGGCAGCAGCAGAAAGCATGACACATGATGTCATTCGGG 6385

|||||||||||||||.||||||||||||||||||||||||||||||||||

S 6348 GAACACTAGGCTGGCTGCAGCAGAAAGCATGACACATGATGTCATTCGGG 6397

R 6386 ATTTACTTGGTGTCAAATTGGACATGACCAACTATGCAAACTTGATAGAG 6435

||||||||||||||||||||||||||||||||||||||||||||||||||

S 6398 ATTTACTTGGTGTCAAATTGGACATGACCAACTATGCAAACTTGATAGAG 6447

R 6436 CAGTACCAGGTTCAAAAGTTAGTAGAAGAGGCTCATCAGCAAACAGAAGA 6485

||||||||||||||||||||||||||||||||||||||||||||||||||

S 6448 CAGTACCAGGTTCAAAAGTTAGTAGAAGAGGCTCATCAGCAAACAGAAGA 6497

R 6486 ATTCCAAGAAAAGGAGCAAGAAATTCTCAACTTAAGGAAGCAGATAACTG 6535

||||||||||||||||||||||||||||||||||||||||||||||||||

S 6498 ATTCCAAGAAAAGGAGCAAGAAATTCTCAACTTAAGGAAGCAGATAACTG 6547

R 6536 ATCTAATGGAGGAAAGACAGAGTTGCATATCCGAAATAAATAAAAAGGAA 6585

||||||||||||||||||||||||||||||||||||||||||||||||||

S 6548 ATCTAATGGAGGAAAGACAGAGTTGCATATCCGAAATAAATAAAAAGGAA 6597

R 6586 GGGGATATAGTGGCAGCCCAGATGACTCTGCAACAACTCCAAGACAGGGA 6635

||||||||.||.||||||||||||||||||||||||||||||||||||||

S 6598 GGGGATATGGTAGCAGCCCAGATGACTCTGCAACAACTCCAAGACAGGGA 6647

R 6636 TCAGTTGCTTTCTGCACAGAACGAGATGTTGAAGGCGGATAAGACCAATT 6685

||||||||||||||||||||||||||||||||||||||||||||||||||

S 6648 TCAGTTGCTTTCTGCACAGAACGAGATGTTGAAGGCGGATAAGACCAATT 6697

R 6686 TAAAGAGGAGAGTGGCGGAACTGGATGAAATGGTAAAAACAATTCTTGGA 6735

||||||||||||||||||||||||||||||||||||||||||||||||||

S 6698 TAAAGAGGAGAGTGGCGGAACTGGATGAAATGGTAAAAACAATTCTTGGA 6747

R 6736 ACACCAACTATACACCAGCCAATTCAACATCCACATACATCCAAGCCCAA 6785

||||||||||||||||||||||||||||||||||||||||||||||||||

S 6748 ACACCAACTATACACCAGCCAATTCAACATCCACATACATCCAAGCCCAA 6797

R 6786 GGCATGTTTTCTGTTTCAGAATAATAGCTCGTTGAAATTGCATGAAATTG 6835

||||||||||||||||||||||||||||||||||||||||||||||||||

S 6798 GGCATGTTTTCTGTTTCAGAATAATAGCTCGTTGAAATTGCATGAAATTG 6847

R 6836 ATTTCACCAAGAGGCTGGAACAGTCTGAGAAGCATCTTTCTCGAGTGAAT 6885

|||||||||||||||||||||||||||||||||||||.||||||||||||

S 6848 ATTTCACCAAGAGGCTGGAACAGTCTGAGAAGCATCTATCTCGAGTGAAT 6897

R 6886 GGTGAACTTGCTCAGTACTTCAAATCTGCAGGTGGCGGTGGCGGTCATCC 6935

||||||||||||||||||||||||||||||||||||||||||||||||||

S 6898 GGTGAACTTGCTCAGTACTTCAAATCTGCAGGTGGCGGTGGCGGTCATCC 6947

R 6936 ACGTGACAAACGCGTGTCGCGTTAG 6960

|||||||||||||||||||||||||

S 6948 ACGTGACAAACGCGTGTCGCGTTAG 6972
